# Targeting Hsp90α to inhibit HMGB1‐mediated renal inflammation and fibrosis

**DOI:** 10.1111/cpr.13774

**Published:** 2024-11-20

**Authors:** Huizhi Wei, Jinhong Ren, Xiue Feng, Chengxiao Zhao, Yuanlin Zhang, Hongxia Yuan, Fan Yang, Qingshan Li

**Affiliations:** ^1^ School of Pharmacy, Medicinal Basic Research Innovation Center of Chronic Kidney Disease, Ministry of Education Shanxi Medical University Taiyuan China; ^2^ Shanxi Key Laboratory of Innovative Drug for the Treatment of Serious Diseases Basing on the Chronic Inflammation Shanxi University of Chinese Medicine Taiyuan China

## Abstract

Renal fibrosis, a terminal manifestation of chronic kidney disease, is characterized by uncontrolled inflammatory responses, increased oxidative stress, tubular cell death, and imbalanced deposition of extracellular matrix. 5,2′‐Dibromo‐2,4′,5′‐trihydroxydiphenylmethanone (LM49), a polyphenol derivative synthesized by our group with excellent anti‐inflammatory pharmacological properties, has been identified as a small‐molecule inducer of extracellular matrix degradation. Nonetheless, the protective effects and mechanisms of LM49 on renal fibrosis remain unknown. Here, we report LM49 could effectively alleviate renal fibrosis and improve filtration function. Furthermore, LM49 significantly inhibited macrophage infiltration, pro‐inflammatory cytokine production and oxidative stress. Interestingly, in HK‐2 cells induced by tumour necrosis factor alpha under oxygen–glucose‐serum deprivation conditions, LM49 treatment similarly yielded a reduced inflammatory response, elevated cellular viability and suppressed cell necrosis and epithelial‐to‐mesenchymal transition. Notably, LM49 prominently suppressed the high‐mobility group box 1 (HMGB1) expression, nucleocytoplasmic translocation and activation. Mechanistically, drug affinity responsive target stability and cellular thermal shift assay confirmed that LM49 could interact with the target heat shock protein 90 alpha family class A member 1 (Hsp90α), disrupting the direct binding of Hsp90α to HMGB1 and inhibiting the nuclear export of HMGB1, thereby suppressing the inflammatory response, cell necrosis and fibrogenesis. Furthermore, molecular docking and molecular dynamic simulation revealed that LM49 occupied the N‐terminal ATP pocket of Hsp90α. Collectively, our findings show that LM49 treatment can ameliorate renal fibrosis through inhibition of HMGB1‐mediated inflammation and necrosis via binding to Hsp90α, providing strong evidence for its anti‐inflammatory and anti‐fibrotic actions.

## Graphical abstract

The polyphenol derivative 5,2′‐dibromo‐2,4′,5′‐trihydroxydiphenylmethanone (LM49) synthesized by our group can effectively ameliorate unilateral ureteral obstruction‐ and folic acid‐induced renal fibrosis, which is connected to the suppression of high‐mobility group box 1 (HMGB1)‐induced inflammation and tubular epithelial cell necrosis. LM49 targets the N‐terminal ATP pocket of heat shock protein 90 alpha family class A member 1 and reduces the interaction of heat shock protein 90 alpha family class A member 1 with HMGB1, thereby further blocking HMGB1 nuclear–cytoplasmic translocation and activation, which provides theoretical evidence supporting the application of LM49 in the prevention and treatment of chronic kidney disease.

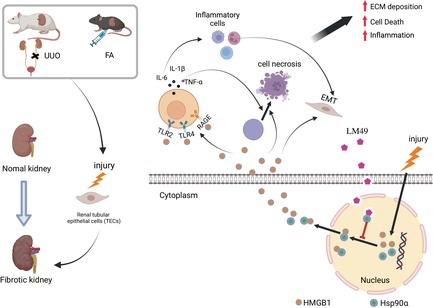

## 1 INTRODUCTION

Renal fibrosis is an inevitable outcome of advancing chronic kidney disease (CKD).^1^ Despite the considerable impact on health and survival, there are limited therapies to effectively halt or reverse the renal fibrogenesis and CKD progression.^2^ Excessive and persistent inflammation and parenchymal cell death have been recognized as the primary pathological mechanisms in the progression of renal fibrosis.^1^, ^3^, ^4^

Tubular epithelial cells (TECs), a major kidney cell type that responds to injuries, have been highlighted as initiators of kidney fibrosis.^1^, ^5^ Consequently, damaged TECs secrete paracrine signals that contain pro‐inflammatory and profibrotic factors into the renal interstitium. This leads to a remodelling of the microenvironment, fostering the progression of both inflammation and fibrosis.^6^ High‐mobility group box 1 (HMGB1), a non‐histone nuclear protein, serves as an intracellular DNA chaperone responsible for repairing and maintaining the stability of chromatin structure. Upon exposure to various stimuli, HMGB1 is released from necrotic cells into the extracellular fluid. Here, it functions as a danger‐associated molecular pattern (DAMP) triggering sterile inflammation, immune cell recruitment and migration, which amplify the inflammatory response and in turn exacerbate further cell death.^7^, ^8^ This autoamplification loop is considered an important factor in the cell death‐inflammation cycle, which is referred to as necroinflammation^9^, ^10^, ^11^ and greatly contributes to the renal fibrosis progression.^12^, ^13^, ^14^ A recent study revealed that in a clinical trial with 110 CKD patients, serum HMGB1 levels were significantly elevated and showed correlation with a decline in glomerular filtration rate, along with markers of inflammation and malnutrition.^15^ In addition, HMGB1 could mediate interleukin (IL)‐1β maturation and release through activating ROS/autophagy/inflammasome pathway. In turn, NLRP3 inflammasome also controlled the release of HMGB1 in asthma.^16^ In another model of rats with adenine‐induced CKD^17^ suggested that HMGB1 may be a potentially meaningful biomarker for CKD with a significantly elevated levels in rat kidneys. These findings imply that HMGB1 may play a crucial role in renal fibrogenesis and that targeting HMGB1 release from activated TECs is emerging as a promising and innovative strategy for treating renal fibrosis.

Heat shock protein 90 alpha family class A member 1 (Hsp90α) is a stress‐responsive protein that serves as a molecular chaperone for misfolded proteins, maintaining cellular balance. This is attributed to attributing to its important role in regulating the stability and functionality of transcription factors, protein kinases and cell signalling proteins in both normal and stressful environments.^18^, ^19^ It has reported that the enhanced interaction between CDC37 and Hsp90 could additionally activate NF‐κB signalling pathway, triggering oxidative stress, inflammatory responses, and endothelial adhesion. These processes ultimately contribute to endothelial dysfunction and atherosclerosis progression.^20^ Encouragingly, Hsp90α has emerged as a promising target for drug development aimed at treating a range of diseases, including cancer and disorders linked to protein misfolding such as renal fibrosis, pulmonary fibrosis and other inflammatory diseases.^21^, ^22^, ^23^, ^24^, ^25^ More importantly, recent evidence has pointed out that Hsp90α participates in the control of HMGB1 localization and secretion.^26^ However, the involvement of Hsp90α‐triggered HMGB1 regulation in the pathogenesis of renal fibrosis remains to be elucidated.

Polyphenolic compounds are valuable resources in the creation of therapeutic treatments for a variety of complex diseases, including renal fibrosis.^27^, ^28^, ^29^, ^30^ In this context, 5,2′‐dibromo‐2,4′,5′‐trihydroxydiphenylmethanone (LM49), a polyphenol derivative derived from marine plants and synthesized by our team, holds significant potential. In our previous studies, LM49 exhibited excellent anti‐inflammatory^31^ and immunomodulatory activities.^32^, ^33^ Moreover, LM49 has been found to demonstrate substantial therapeutic effects in acute pyelonephritis^33^ and attenuate extracellular matrix (ECM) degradation in rats with diabetic nephropathy.^34^ However, the exact molecular mechanisms responsible for the anti‐inflammatory properties and anti‐fibrotic effects of LM49 in renal fibrosis remain largely elusive.

In this investigation, we revealed that LM49 can effectively ameliorate renal fibrosis and filtration function in kidneys with unilateral ureteral obstruction (UUO)‐ and folic acid (FA)‐induced fibrosis, which is related to the suppression of HMGB1‐induced inflammation and TECs necrosis. We innovatively find that LM49 directly targets to Hsp90α N‐terminal ATP pocket and reduces the interaction of Hsp90α with HMGB1, thereby further blocking HMGB1 nuclear–cytoplasmic translocation and activation. These findings highlight the pivotal roles of Hsp90α and HMGB1 in UUO‐ and FA‐related renal fibrosis and suggest the potential therapeutic value of LM49 in CKD treatment in the clinical.

## 2 METHODS

### 2.1 Animal studies of fibrosis

Male Sprague–Dawley rats weighting 200 ± 20 g (6–8 weeks old) and male C57BL/6J mice weighting 20 ± 2 g (6–8 weeks old) were acquired from SPF Biotechnology Co., Ltd. (Beijing, China). Renal fibrosis model was created using UUO in Sprague–Dawley rats or through FA (250 mg/kg) induction in C57BL/6J mice, as previously reported.^1^ These models have been extensively utilized for investigating the pathophysiology of advancing CKD and the mechanisms involved in progression of renal fibrosis.^1^, ^35^, ^36^, ^37^ Sham‐operated rats underwent the same procedure in the absence of ligation, while mice injected with sodium bicarbonate were used as the vehicle control (saline).

### 2.2 LM49 treatment in vivo

In UUO‐induced model, a total of 56 rats were randomly divided into seven groups, eight rats per group: sham, sham + LM49 (45 mg/kg)‐treated, UUO, UUO + low‐dose LM49 (22.5 mg/kg)‐treated, UUO + medium‐dose LM49 (45 mg/kg)‐treated, UUO + high‐dose LM49 (90 mg/kg)‐treated and UUO + losartan (10 mg/kg)‐treated. For in vivo experiments, LM49 or losartan was dissolved in 0.5% CMC‐Na solution. After UUO, the rats were treated with LM49 by oral administration once a day for 21 days beginning immediately after UUO. The rats in the sham group were given an equivalent volume of 0.5% CMC‐Na solution. Twenty‐one days after surgery, the rats were euthanised under anaesthesia.

Similarly, in FA‐induced model, 56 mice were randomly assigned to seven groups, each consisting of eight mice: normal control, LM49 (65 mg/kg)‐treated, FA‐treated, FA + low‐dose LM49 (32.5 mg/kg)‐treated, FA + medium‐dose LM49 (65 mg/kg)‐treated, FA + high‐dose LM49 (130 mg/kg)‐treated and FA + losartan (15 mg/kg)‐treated. Mice were orally administration with different concentrations of LM49 (32.5, 65 and 130 mg/kg) or losartan once daily on the seventh day after FA injection. Mice in the control group were given saline at an equivalent volume and frequency to those receiving LM49. Twenty‐eight days after FA injection, mice were euthanised under anaesthesia.

### 2.3 ETHICS STATEMENT

All animal experiments were carried out in compliance with the ethical policies and procedures approved by the Institutional Animal Care and with the permission of the Ethics Committee of Shanxi University of Chinese Medicine (Permit No. 2022DW171).

### 2.4 Histological analysis

In brief summary, the renal tissues were preserved in 4% paraformaldehyde, embedded in paraffin, and sectioned into 4 μm slices, then followed by staining with haematoxylin–eosin (HE). The tubules injury index and tubulointerstitial inflammation index were evaluated as reported in previous study.^1^ To assess the area of fibrosis and interstitial collagen, Masson's Trichrome and picrosirius red staining were utilized. Image acquisition was conducted using a Hamamatsu Nano Zoomer 2.0 RS scanner. The fibrotic area quantification was counted by Image Pro‐Plus software (Media Cybernetics, USA).

### 2.5 ELISA assay

Serum creatinine (Scr) and urea nitrogen concentrations were determined respectively, following the instructions supplied by the manufacturer (Nanjing Jiancheng Bioengineering Institute, China).

### 2.6 Immunohistochemistry and immunofluorescence staining

To sum up, the tissue sections were exposed to primary antibodies and left overnight at 4°C for incubation, followed by incubation with the corresponding secondary antibodies. Finally, immunohistochemistry (IHC) image was captured using an ECLIPSE Ti2 microscope (Nikon, Japan).

For tissue immunofluorescence staining, the sections were treated with primary antibodies overnight at 4°C. After the application of fluorescently labelled secondary antibodies, slides were counterstained with DAPI and subsequently examined applying an FV3000 confocal scanning microscope, which was used to capture images of the samples. For cells immunofluorescence staining, 96‐well plates were used with three wells per group. Following incubation with corresponding administrations, cells were treated with paraformaldehyde for 1 h at 4°C, punched with 0.25% triton (dissolved in PBS) at room temperature (RT) for 30 min. Following this, the cells were treated with 1% BSA (dissolved in PBS) for 1 h at RT, then incubated with primary antibodies overnight at 4°C. Subsequently, Alexa Fluor 488 or 647‐labelled secondary antibodies were added and incubated for 1 h at RT. Images were captured for each well using the ImageXpress Micro four High Content Imaging System, as in previous studies, with nine sites at 488 and 647 nm. To quantify co‐localization, the Image J analysis software was used to analyse the images from each experiment.

### 2.7 Transmission electron microscopy

The kidneys were fixed in a solution of 2.5% glutaraldehyde for 24 h and then embedded in resin following standard protocols. The embedded samples were examined using a transmission electron microscope (TEM) operating at 80 kV (Hitachi, Japan).

### 2.8 Quantitative real‐time PCR

Total RNA was acquired from kidneys or cells utilizing Trizol Reagent (Takara, Japan). Subsequently, according to the instructions, cDNA was synthesized with a reverse transcription kit (Takara, Japan). SYBR Premix Ex Taq (Takara, Japan) and 7900HT Real‐Time PCR system (Applied Biosystems, USA) were used to quantified the gene expression levels, which were then calculated with the equation 2^−ΔΔCt^ method. The primers sequences used are listed in Table S1.

### 2.9 Cell culture, transfection and treatment

Human proximal TECs (HK‐2) and rat renal TECs (NRK‐52E) were sourced from the Cell Bank of the Chinese Academy of Sciences in Shanghai, China. HK‐2 cells and NRK‐52E cells were grown in DMEM/F12 and high‐glucose DMEM medium containing 10% foetal bovine serum (Gibco, USA) and 1% penicillin (Beyotime, China), respectively.

To mimic the renal microenvironment of ischemia, hypoxia, and inflammatory factor infiltration in vivo as reported previously,^38^ HK‐2 and NRK‐52E cells were treated with 20 ng/mL tumour necrosis factor alpha for 12 h under the conditions of oxygen–glucose‐serum deprivation (TNF‐α/OGSD). In contrast, cells in control group were cultured in normal culture conditions. Upon reaching about 80% confluence, cells were pre‐treated with different concentrations of LM49 for 24 h, HMGB1 inhibitor glycyrrhetinic acid (GA) 20 μg/mL for 24 h or Hsp90α inhibitor geldanamycin 0.5 μM for 12 h, followed by incubation with recombinant TNF‐α (20 ng/mL) under the conditions of OGSD for the indicated time.

The plasmid pcDNA 3.1‐HMGB1 and pcDNA 3.1‐Hsp90α were generated by inserting the human HMGB1 or Hsp90α coding sequence into the pcDNA3.1 vector. Cells in six‐well plates were transfected with pcDNA3.1 vector control, pcDNA 3.1‐HMGB1 or pcDNA 3.1‐Hsp90α by using Lipofectamine 2000 (Invitrogen, USA). The plasmids were procured from GenePharma (China). Hsp90α siRNA (5′‐CUUCACAGACUUGUCGUUCUU‐3′) were purchased from GenePharma (Shanghai, China). Cells were transfected with 20 or 150 p.m. of targeted siRNA in 96‐well or six‐well plates, respectively, using GP‐transfect‐Mate (GenePharma, Shanghai, China). The knockdown effect of siRNA was evaluated by Immunoblotting or qRT‐PCR.

### 2.10 Flow cytometry

Kidneys were removed and minced into fragments after perfusion with 1× PBS, then digested in DMEM/F12 containing 1 mg/mL collagenase (Gibco, USA) and 0.1 mg/mL DNase (Roche, Basel, Switzerland) at 37°C. Single‐cell suspension was obtained through 40 μm mesh. Following antibodies were used from BioLegend for FACS analysis: Zombie APC‐A750 staining to determine renal viable cells; PE/Cy7‐conjugated anti‐mouse CD11b Ab; PE‐conjugated anti‐rats CD68 Ab; PE‐conjugated anti‐mouse F4/80 Ab; FITC‐conjugated anti‐mouse CD45 Ab.

### 2.11 Co‐immunoprecipitation

The lysate obtained by lysis and centrifugation of cells and tissues was measured using BCA to obtain protein concentration information. The lysates, containing 1 mg of protein, were incubated with control agarose resin for 3 h at 4°C. Subsequently, they were subjected to overnight incubation at 4°C with anti‐Hsp90α, anti‐HMGB1, or anti‐IgG. The co‐precipitations were then exposed to protein A + G agarose beads for an additional 4 h. After washing the beads five times with immunoprecipitation (IP) lysis/wash buffer, they were boiled in loading buffer. Subsequently, Western blotting was conducted using the specified antibodies.

### 2.12 Western blotting

Kidneys or cells were collected in RIPA buffer supplemented with a Complete Protease Inhibitor. After centrifuging for 15 min (12,000 rpm), lysates were quantified by the BCA protein assay (Boster, China). After resolving by SDS–PAGE, total proteins were transferred onto PVDF membrane. Subsequently, specific primary antibodies and secondary antibodies were successively applied to the membrane. Images were detected, captured and photographed by Amersham Imager 600 (Cytiva, United States).

### 2.13 RNA‐seq analysis

Total RNA was obtained from the rats or mice kidneys. RNA integrity, purity and concentration were analysed. Short fragments were obtained by randomly broken after centralized mRNA using magnetic beads with oligo (dT). The isolated mRNA was broken into small fragments and converted into cDNA through reverse transcription. The cDNA fragments were then ligated with sequencing adapters and underwent PCR amplification. The resulting cDNA libraries were sequenced using high‐throughput sequencing technology. The initial sequencing data underwent processing to eliminate adapter sequences and low‐quality reads. Afterward, the high‐quality reads were mapped to the reference genome using alignment software. Differential gene expression analysis was conducted to determine genes differentially expressed between experimental conditions. Functional enrichment analysis was conducted to investigate the biological pathways linked to the differentially expressed genes (DEGs).

### 2.14 Reagents and antibodies

LM49 was synthesized as previously described.^39^ ELISA kits for Scr (C011‐2‐1), blood urea nitrogen (C013‐2‐1), malondialdehyde (A003‐1‐2) and superoxide dismutase (A001‐3‐2) were sourced from Jiancheng Bioengineering Research Institute in Nanjing, China. Lactate dehydrogenase (LDH) Cytotoxicity Assay Kit (C0017), propidium iodide (PI)/Hoechst apoptosis, necrosis assay kit (C1056) and CCK‐8 assay kit (C0038) were acquired from Beyotime Biotechnology in Shanghai, China. Recombinant TNF‐α was bought from R&D Systems (210‐TA‐020) located in Minneapolis, MN, USA. GA (HY‐N0184) and geldanamycin (HY‐15230) were obtained from MCE (Beijing, China). Antibodies against collagen type I (COL1; ab270946), FN (ab2413), HSP90 (ab13495), F4/80 (ab16911) and CD68 (ab283654) were sourced from Abcam (Cambridge, MA, USA). Anti‐E‐cadherin (14472), anti‐alpha‐smooth muscle actin (α‐SMA) (19245) and anti‐rabbit IgG (7074) were acquired from Cell Signalling Technology (Beverly, MA, USA). Anti‐HMGB1 (PA5‐32249) was sourced from Invitrogen (Carlsbad, CA, USA). Antibodies against GAPDH (60004‐1‐Ig) and H3 (17168‐1‐AP) were gathered from Proteintech (Wuhan, China). The Lipofectamine 2000 reagent (11668500) was gathered from Invitrogen (Carlsbad, CA, USA). HMGB1 ELISA kit (EEL102) was obtained from Invitrogen (Carlsbad, CA, USA). IP was performed following manufacturers protocols of a commercial Pierce Classic IP Kit (26146, Thermo Fisher).

### 2.15 Statistics

The mean ± standard deviation (SD) of all data was presented from 3 independent experiments. Statistical analysis in multiple groups involved conducting one‐way ANOVA followed by the Tukey's test. In two groups, an unpaired two‐tailed t‐test was performed. Statistical analyses were conducted utilizing GraphPad Prism 8.0 software from San Diego, USA. A two‐tailed *p*‐value of <0.05 was regarded as statistically significant.

## 3 RESULTS

### 3.1 LM49 ameliorates UUO‐ and FA‐induced renal fibrosis and filtration function

**FIGURE 1:**
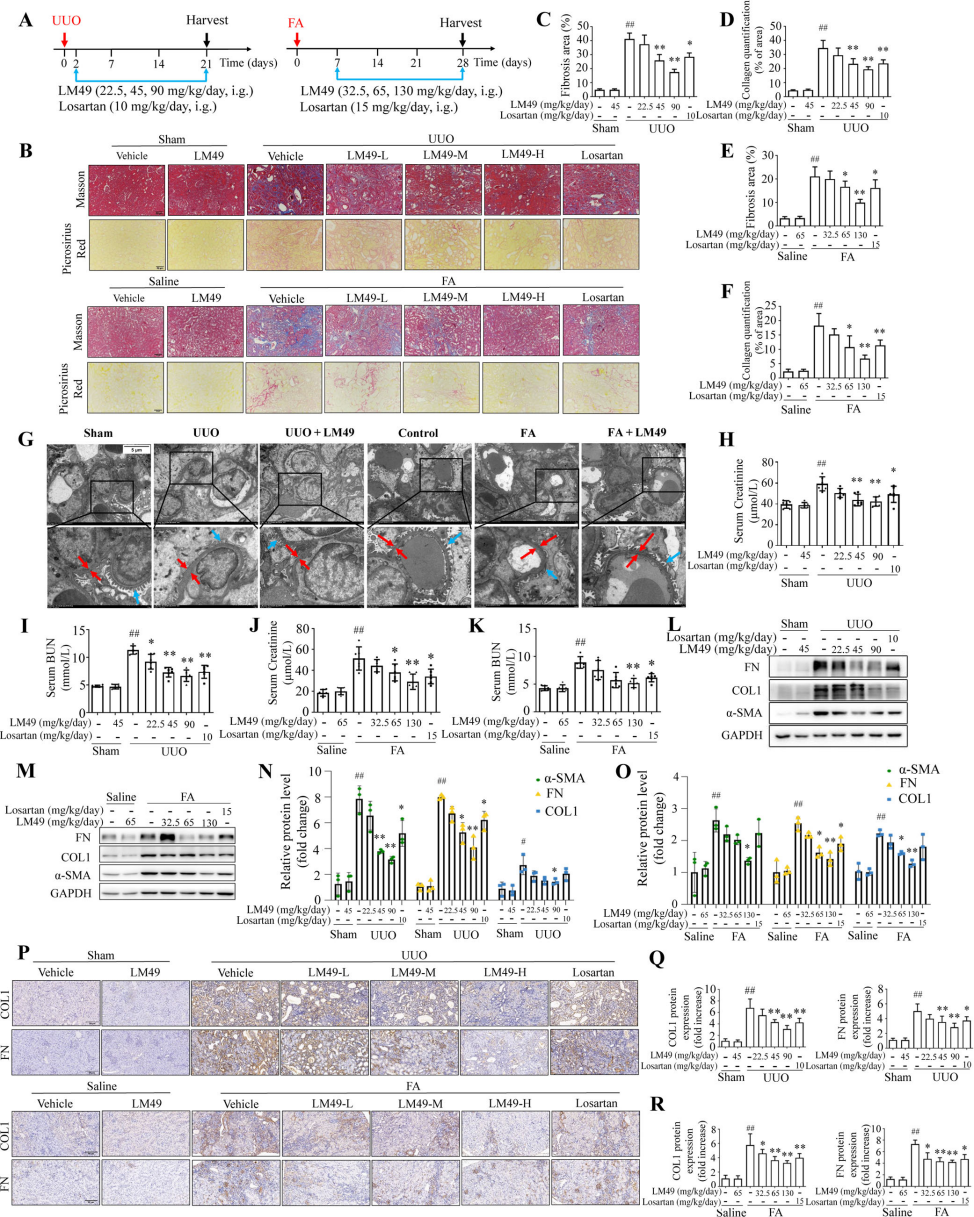
5,2′‐Dibromo‐2,4′,5′‐trihydroxydiphenylmethanone (LM49) ameliorates unilateral ureteral obstruction (UUO)‐ and folic acid (FA)‐induced renal fibrosis and filtration function. (A) The scheme of LM49 treatment in UUO‐induced rats and FA‐induced mice were illustrated. (B) Representative kidney cross‐sections stained with Masson's trichrome (Scale bar, 50 μm) or picrosirius red (Scale bar, 50 μm), ×200. (C, D) Statistical results for fibrosis area ([C] Masson's trichrome) and interstitial collagen quantification ([D] picrosirius red) in the kidneys of UUO rats in (D) analysed by Image Pro‐Plus software (mean ± SD, *n* = 3 per group, ^##^ *p* < 0.01 vs. sham group; *p < 0.05, **p < 0.01 vs. UUO group). (E, F) Statistical results for fibrosis area ([E] Masson's trichrome) and interstitial collagen quantification ([F] picrosirius red) in the kidneys of FA mice in (B) analysed by Image Pro‐Plus software (mean ± SD, *n* = 3, ^##^ *p* < 0.01 vs. control group; *p < 0.05, **p < 0.01 vs. FA group). (G) Representative transmission electron microscope (TEM) images of glomerular basement membrane (red arrow) and the foot process (blue arrow) in kidney glomerulus, *n* = 3. Scale bar, 5 μm. (H, I) Effect of LM49 or losartan on UUO‐induced serum creatinine (Scr) (H) or serum blood urea nitrogen (BUN) (I) levels (mean ± SD, *n* = 6 per group, ^##^ *p* < 0.01 vs. sham group; *p < 0.05, **p < 0.01 vs. UUO group). (J, K) Effect of LM49 or losartan on FA‐induced Scr (J) or serum BUN (K) levels (mean ± SD, *n* = 6 per group, ^##^ *p* < 0.01 vs. control group; *p < 0.05, **p < 0.01 vs. FA group). (L–O) Western blot (L, M) and quantitative analysis (N, O) for alpha‐smooth muscle actin (α‐SMA), fibronectin and COL1 expression levels of kidney tissue lysates in UUO rats or FA mice (mean ± SD, *n* = 3, ^#^ *p* < 0.05, ^##^ *p* < 0.01 vs. sham or control group; *p < 0.05, **p < 0.01 vs. UUO or FA group). (P) Representative kidney cross‐sections stained with immunohistochemical staining with COL1 or FN (Scale bar, 50 μm), ×200. (Q, R) Quantification of immunohistochemical staining of COL1 or FN in the kidneys from UUO rats (Q) or FA mice (R) (mean ± SD, *n* = 3, ^##^ *p* < 0.01 vs. sham or control group; *p < 0.05, **p < 0.01 vs. UUO or FA group).

To evaluate the pharmacological activities of LM49 in the pathogenesis of renal fibrosis, we first established two fibrotic kidney models using UUO in rats and FA in mice. LM49 was administered to rats or mice at doses equivalent to those used in previous trials of diabetic nephropathy therapy^34^ (Figure 1A). Losartan at a dose equivalent to clinical usage was used as a positive control (Figure 1A).

Masson's trichrome and picrosirius red staining results exhibited obvious pathological changes in UUO rats and FA mice, including tubular vacuolization, tubular atrophy and collagen fibre deposition (Figure 1B), indicating that fibrotic kidney models were successfully established. Notably, after treatment with LM49 or losartan, the degree of pathological changes improved notably compared to that in the model group (Figure 1B–F). Moreover, this improvement was more significant after high‐dose LM49 treatment (Figure 1B–F). Similarly, high‐dose LM49 intervention protected the integrity of the renal tubules (Figure 1B). In addition, TEM analysis showed thickening of the glomerular basement membrane (red arrow), and foot process fusion in the glomerulus (blue arrow) in fibrotic kidneys (Figure 1G). However, compared with their untreated counterparts, LM49 treatment mitigated these histopathological alterations (Figure 1G). Furthermore, increased levels of the kidney injury markers Scr and blood urea nitrogen were also observed in UUO rats and FA mice, and these increases were inhibited to varying degrees by LM49 or losartan treatment compared to those model group (Figure 1H–K), indicating the normalization of renal filtration functions. We subsequently investigated the impact of LM49 on the expression levels of fibronectin (FN), α‐SMA and COL1 as the markers of fibrosis in fibrotic kidneys by immunoblot analysis. As shown in Figure 1L–O, higher protein expression levels of FN, α‐SMA and COL1 were detected in the fibrotic kidneys, whereas these changes were mitigated in a dose‐dependent manner following LM49 treatment. Subsequent IHC staining confirmed the fibrosis‐associated changes in the expression of FN and COL1 in UUO and FA groups, which were ameliorated by LM49 in a dose‐dependent manner (Figure 1P–R). Collectively, these results indicated that LM49 ameliorated renal damage, loss of filtration function and fibrogenesis in UUO‐ and FA‐induced fibrotic kidneys.

### 3.2 LM49 treatment effectively attenuates UUO‐ and FA‐induced inflammation in fibrotic kidneys

**FIGURE 2:**
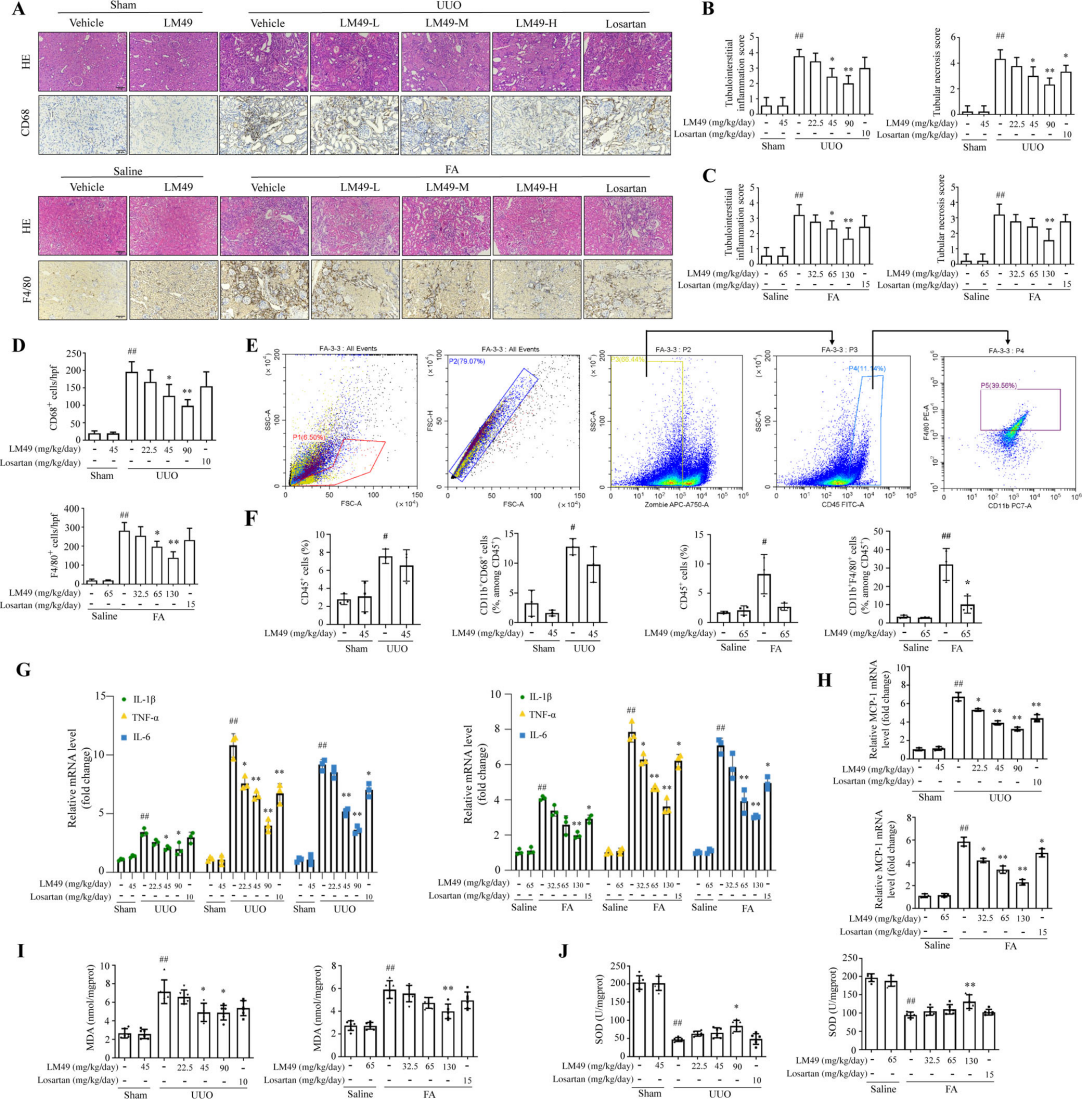
5,2′‐Dibromo‐2,4′,5′‐trihydroxydiphenylmethanone (LM49) treatment effectively attenuates unilateral ureteral obstruction (UUO)‐ and folic acid (FA)‐induced inflammation in fibrotic kidneys. (A) Representative kidney cross‐sections stained with haematoxylin and eosin (HE) in UUO rats or FA mice (Scale bar, 50 μm), ×200, immunohistochemical staining with CD68 in UUO rats or F4/80 in FA mice (Scale bar, 50 μm), ×200. (B) Morphometric analysis assessing tubulointerstitial inflammation index and tubular necrosis scores in UUO‐induced kidneys (mean ± SD, *n* = 3, ^##^ *p* < 0.01 vs. sham group; *p < 0.05, **p < 0.01 vs. UUO group). (C) Morphometric analysis assessing tubulointerstitial inflammation index and tubular necrosis scores in FA‐induced kidneys (mean ± SD, *n* = 3, ^##^ *p* < 0.01 vs. control group; *p < 0.05, **p < 0.01 vs. FA group). (D) Quantification of immunohistochemical staining of macrophages biomarkers CD68 in kidneys from UUO rats or F4/80 in kidneys from FA mice (mean ± SD, *n* = 3, ^##^ *p* < 0.01 vs. sham or control group; *p < 0.05, **p < 0.01 vs. UUO or FA group). (E) Representative images of schematic diagram of the loop gate for detecting the number of total leukocytes and macrophages in the kidney using flow cytometry. (F) Quantitative results of the effect of LM49 on the number of total leukocytes and macrophages in kidneys of UUO rats or FA mice (mean ± SD, *n* = 3, ^#^ *p* < 0.05 vs. sham or control group; *p < 0.05 vs. UUO or FA group). (G) mRNA expression levels of proinflammatory factor interleukin‐1β (IL‐1β), tumour necrosis factor‐α (TNF‐α), and IL‐6 in fibrotic kidneys (mean ± SD, *n* = 3, ^##^ *p* < 0.01 vs. sham or control group; *p < 0.05, **p < 0.01 vs. UUO or FA group). (H) mRNA expression levels of chemokines monocyte chemoattractant protein‐1 (MCP‐1) in fibrotic kidneys (mean ± SD, *n* = 3, ^##^ *p* < 0.01 vs. sham or control group; *p < 0.05, **p < 0.01 vs. UUO or FA group). (I, J) malondialdehyde (MDA) levels and superoxide dismutase (SOD) levels of kidneys from each group in UUO rats or FA mice (mean ± SD, *n* = 6 per group, ^##^ *p* < 0.01 vs. sham or control group; *p < 0.05, **p < 0.01 vs. UUO or FA group).

Inflammation plays a pivotal role in the advancement of fibrosis in CKD,^4^ and LM49 has shown excellent anti‐inflammatory activity in several animal disease models in our previous studies.^32^, ^33^ These findings encouraged us to assess the effect of LM49 on the inflammatory response in fibrotic kidneys. The HE staining results showed that kidneys treated with UUO or FA exhibited severe interstitial inflammation, whereas LM49 treatment significantly improved the pathological features induced in the fibrotic kidneys (Figure 2A–C). Additionally, the expression levels of the macrophage markers CD68 in rats and F4/80 in mice were determined by conducting IHC staining on the kidneys of each group (Figure 2A). We found that the proportion of CD68‐positive or F4/80‐positive areas in the total area was significantly elevated in fibrotic kidneys following UUO or FA injury but was inhibited by LM49 administration in a dose‐dependent manner (Figure 2A,D). This was further confirmed by flow cytometry (Figure 2E,F), which showed increased infiltration of total number of leukocytes and macrophages in fibrotic kidneys, which was reversed by LM49 treatment (Figure 2F), suggesting that LM49 treatment inhibited inflammatory cell infiltration in fibrotic kidneys. Moreover, we measured the mRNA levels of inflammatory activation biomarkers IL‐1β, IL‐6 and TNF‐α. Consistently, the data revealed that UUO‐ or FA‐induced increased expression levels of IL‐1β, TNF‐α and IL‐6, which were reversed by LM49 in a dose‐dependent manner (Figure 2G). In line with this, the increased mRNA levels of monocyte chemoattractant protein‐1 (MCP‐1) in fibrotic kidneys were also reversed by LM49 intervention (Figure 2H), suggesting that LM49 attenuated the migration and infiltration of inflammatory cytokines in fibrotic tissues. Disruption of redox homeostasis in the tissue microenvironment is a pivotal factor that triggers inflammation and stimulates signalling cascades in the innate immune system, resulting in tissue dysfunction.^40^, ^41^ As expected, markedly elevated malondialdehyde levels and reduced superoxide dismutase levels were observed in UUO‐ or FA‐induced kidneys compared with the sham or control groups (Figure 2I,J). Nevertheless, LM49 treatment significantly reversed these alterations (Figure 2I,J), indicating that LM49 protects fibrotic kidneys against oxidative stress‐induced kidney inflammation and dysfunction. Collectively, these data provide evidence that LM49 suppresses the inflammatory response and activation in UUO‐ and FA‐induced renal fibrosis.

### 3.3 LM49 treatment diminishes TECs necroinflammation and inhibits the nucleocytoplasmic translocation and release of HMGB1 in fibrotic kidneys

**FIGURE 3:**
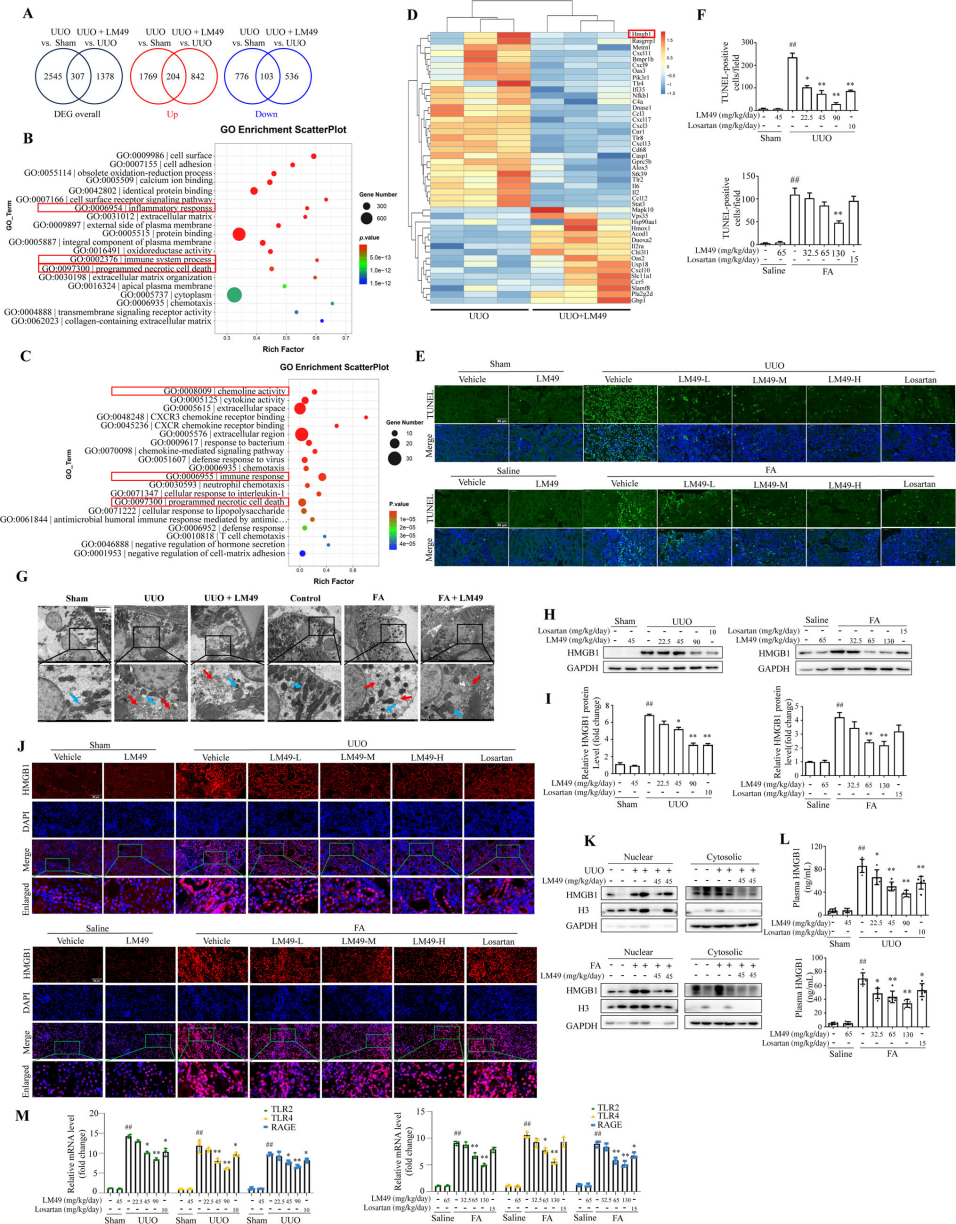
5,2′‐Dibromo‐2,4′,5′‐trihydroxydiphenylmethanone (LM49) treatment diminishes tubular epithelial cells necroinflammation and inhibits the nucleocytoplasmic translocation and release of high‐mobility group box 1 (HMGB1) in fibrotic kidneys. (A) Venn diagram suggesting the number of differentially expressed genes, the number of upregulated genes and downregulated genes in kidneys of rats (sham rats, LM49‐treated unilateral ureteral obstruction [UUO] rats) compared with UUO rats. (B) Gene Ontology (GO) analysis of the differential genes in kidneys from UUO rats versus sham rats. (C) GO analysis of the differential genes in kidneys from UUO rats versus LM49‐treated UUO rats. (D) Heat map of differentially expressed genes related to inflammation and necrosis in UUO rats versus LM49‐treated UUO rats. (E) Representative images of TUNEL staining of kidney sections. TUNEL signal (green) and DAPI (blue) were shown. (F) Quantitative analysis of TUNEL‐positive cells in fibrotic kidneys of UUO rats and FA mice, mean ± SD, *n* = 3, ^##^ *p* < 0.01 versus sham or control group; *p < 0.05, **p < 0.01 versus UUO or FA group. (G) Representative electron micrographs of tubular necrotic cells (red arrow) and mitochondrial damage (blue arrow) in proximal tubular cells. *n* = 3. Scale bar = 5 μm. (H, I) Western blotting (H) and quantitative results (I) of HMGB1 in kidneys of UUO rats or FA mice (mean ± SD, *n* = 3, ^##^ *p* < 0.01 vs. sham or control group; *p < 0.05, **p < 0.01 vs. UUO or FA group). (J) Immunofluorescence staining of HMGB1 (red) in kidneys of UUO rats or FA mice, Scale bars, 50 μm, ×400. (K) Western blotting of the nuclear and cytoplasmic HMGB1 levels in fibrotic kidneys of UUO rats or FA mice, *n* = 4 per group. (L) HMGB1 levels in plasmas of UUO rats or FA mice were measured using ELISA (mean ± SD, *n* = 6, ^##^ *p* < 0.01 vs. sham or control group; *p < 0.05, **p < 0.01 vs. UUO or FA group). (M) mRNA expression levels of Toll‐like receptor (TLR)‐2, TLR‐4 and receptor for advanced glycation end products (RAGE) in kidneys of UUO rats or FA mice.

Newly emerging evidence indicates the involvement of necroinflammation in the development of obstructive nephropathy,^9^, ^42^, ^43^ acute kidney injury^43^ and the progression of acute kidney injury to CKD.^12^ Moreover, based on the anti‐inflammatory activity and protective effects of LM49 on renal tubular integrity, we investigated whether necrosis of TECs in fibrotic kidneys could be suppressed by LM49. We first sequenced the mRNA of kidney tissues from sham, UUO and LM49 (45 mg/kg/day)‐treated UUO rats or control, FA and LM49 (65 mg/kg/day)‐treated FA mice. We found 204 upregulated genes and 103 downregulated genes in UUO + LM49 (45 mg/kg/day)‐treated rats and sham rats compared to UUO rats (Figure 3A), and 154 upregulated genes and 72 downregulated genes in FA + LM49 (65 mg/kg/day)‐treated mice and control mice compared to those in FA‐treated mice (Figure S1A). Moreover, Gene Ontology analysis demonstrated that the DEGs prominently altered in UUO rats or FA mice were more enriched in the immune system process, regulation of inflammatory response and immune response compared with those in sham rats or control mice (Figure 3B and Figure S1B). The DEGs that were altered upon administration of LM49 in UUO rats or FA mice were also related to the immune response, chemokine activity, inflammatory response and regulation of acute inflammatory response (Figure 3C and Figure S1C). These findings were consistent with the reduced inflammatory cells infiltration of LM49 in fibrotic kidneys (Figure 2A–C). More importantly, programmed necrotic cell death was also observed in UUO rats compared with sham rats (Figure 3B) and after LM49 treatment (Figure 3C), as well as the cellular response to tumour necrosis factor and negative regulation of the necroptotic process in FA mice compared with control mice and LM49 administration groups (Figure S1B,C), indicating that LM49 may be actively involved in tissue inflammation clearance and the alleviation of cell necrosis. Subsequently, the DEGs related to inflammation and necrosis were further demonstrated using a heat map (Figure 3D and Figure S1D). To test this, we first performed TUNEL staining to confirm a significantly increased percentage of TUNEL staining‐positive TECs in fibrotic kidneys (Figure 3E,F) compared to their sham or control littermates. As expected, the percentage of TUNEL staining‐positive TECs remarkably diminished in a dose‐dependent manner after LM49 intervention (Figure 3E,F). This was consistent with the noticeable relief of renal TEC necrosis, tubular atrophy, and vacuolization after LM49 treatment demonstrated using HE staining (Figure 2A–C). TEM analysis further revealed the morphology of TECs and a large number of small vesicles, cell membrane rupture, cell swelling or deformation were observed in fibrotic kidneys, whereas these changes were markedly ameliorated after LM49 intervention (Figure 3G, red arrow). Moreover, there were clearly impaired mitochondrial cristae in fibrotic kidney TECs, which was reversed by LM49 treatment (Figure 3G, blue arrow). These data virtually indicate that LM49 plays a notable role in protecting TECs from inflammatory injury and necrosis in fibrotic kidneys.

Interestingly, we found that HMGB1, a key DAMP for amplifying both inflammation and necrosis,^15^ was elevated in both animal fibrosis models, whereas it decreased after LM49 treatment (Figure 3D and Figure S1D), suggesting that it may serve as a collective target gene for LM49 to mediate necroinflammation. Next, we determined the expression levels of HMGB1 in fibrotic kidneys with and without LM49 intervention. As shown in Figure 3H,I, there were apparently increased expression levels of HMGB1 in fibrotic kidneys, whereas LM49 treatment reversed these changes, especially in the high‐dose LM49 treatment groups. To provide additional evidence of noticeable changes in the localization of HMGB1, immunofluorescence staining revealed that in sham‐operated or control kidneys, HMGB1 was mainly situated in the nuclei of TECs. Conversely, a significant amount of HMGB1 was observed to migrate from the nucleus to the cytoplasm of TECs in fibrotic kidneys (Figure 3J). Intriguingly, following LM49 treatment, the translocation of nuclear HMGB1 to the cytoplasm was significantly reduced (Figure 3J). The translocation was further detected using immunoblotting. Similarly, compared with the sham or control group, HMGB1 was markedly increased in the nucleus and cytoplasm of fibrotic kidneys, whereas LM49 treatment significantly reduced the nuclear export of HMGB1 (Figure 3K). Next, we assessed the degree of HMGB1 release into the serum circulation of UUO rats and FA mice using ELISA. The data showed that the levels of HMGB1 were low in the sham rats or control mice, whereas serum levels of HMGB1 were notably elevated in UUO rats and FA mice (Figure 3L). However, treatment with LM49 decreased the levels of HMGB1 in the serum (Figure 3L), indicating that LM49 could reduce HMGB1 release. Furthermore, we assessed the mRNA expression of several key HMGB1 receptors in the kidneys, and the results showed that compared with sham rats or control mice, there was a strong upregulation of Toll‐like receptor (TLR)‐2, TLR‐4 and the receptor for advanced glycation end products in fibrotic kidneys (Figure 3M). However, the LM49 treatment significantly mitigated this increase (Figure 3M). Taken together, these data indicate that LM49 treatment can diminish TECs necrosis and inflammation and inhibit HMGB1 nucleocytoplasmic translocation and release.

### 3.4 LM49 protects TECs treated with TNF‐α/OGSD against necroinflammation and epithelial‐to‐mesenchymal transition in vitro

**FIGURE 4:**
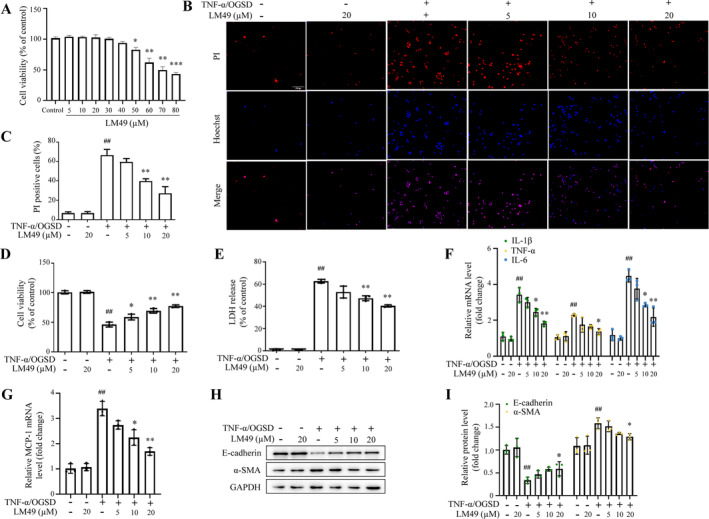
5,2′‐Dibromo‐2,4′,5′‐trihydroxydiphenylmethanone (LM49) protects tubular epithelial cells treated with tumour necrosis factor alpha for 12 h under oxygen–glucose‐serum deprivation conditions (TNF‐α/OGSD) against necroinflammation and epithelial‐to‐mesenchymal transition in vitro. (A) Effect of LM49 alone on HK‐2 cells viability (mean ± SD, *n* = 3, *p < 0.05, **p < 0.01 vs. control). (B, C) Representative images (B) and quantification (C) of Hoechst/propidium iodide (PI) staining, Scale bars, 100 μm (mean ± SD, *n* = 3, ^##^ *p* < 0.01 vs. control; **p < 0.01 vs. model). (D) HK‐2 cells viability treated with LM49 in the absence or presence of 20 ng/mL TNF‐α/OGSD (mean ± SD, *n* = 3, ^##^ *p* < 0.01 vs. control; *p < 0.05, **p < 0.01 vs. model). (E) Lactate dehydrogenase (LDH) release of HK‐2 cells treated with LM49 in the absence or presence of TNF‐α/OGSD for 12 h (mean ± SD, *n* = 3, ^##^ *p* < 0.01 vs. control; **p < 0.01 vs. model). (F, G) mRNA expression levels of proinflammatory factor interleukin (IL)‐1β, TNF‐α and IL‐6 (F) and chemokines monocyte chemoattractant protein‐1 (MCP‐1) (G) in HK‐2 cells treated with LM49 in the absence or presence of TNF‐α/OGSD for 12 h (mean ± SD, *n* = 3, ^##^ *p* < 0.01 vs. control; *p < 0.05, **p < 0.01 vs. model). (H, I) Western blotting (H) and quantitative results (I) of E‐cadherin and alpha‐smooth muscle actin (α‐SMA) in the HK‐2 cells treated with LM49 in the absence or presence of TNF‐α/OGSD for 12 h (mean ± SD, *n* = 3, ^##^ *p* < 0.01 vs. control; *p < 0.05 vs. model).

Previous studies have shown that ischemia, hypoxia and TNF‐α together contribute to pathophysiological changes in the kidneys following ureteral obstruction and FA treatment in vivo.^42^, ^44^, ^45^, ^46^, ^47^ Thus, using human renal epithelial cell line HK‐2 cells, we constructed an in vitro model stimulated by TNF‐α treatment under the conditions of OGSD, which as reported in previous studies, mimics the in vivo environment.^38^, ^48^, ^49^ To avoid toxic effects of LM49 on HK‐2 cells, we first determined that LM49 at concentrations below 40 μM had no significant effects on HK‐2 cells viability (Figure 4A). Next, we found that in HK‐2 cells treated with TNF‐α/OGSD there were a remarkable rise in the percentage of Hoechst and PI staining double positive HK‐2 cells, a decrease in cell viability (assayed using CCK‐8), and an elevation in LDH release. These changes were effectively reversed by LM49 intervention (Figure 4B–E), suggesting LM49 could reduce cell membrane disruption and protect against HK‐2 cells necrosis. Additionally, qRT‐PCR results showed that mRNA levels of the chemokines MCP‐1 and inflammatory factors IL‐1β, IL‐6 and TNF‐α were obviously elevated in the HK‐2 cells upon TNF‐α/OGSD treatment, whereas these increases were inhibited by LM49 treatment (Figure 4F,G). Moreover, the elevated protein levels of mesenchymal cell markers α‐SMA, and reduced levels of epithelial cell marker E‐cadherin were noted in the HK‐2 cells treated with TNF‐α/OGSD, indicating the occurrence of epithelial‐to‐mesenchymal transition (EMT), which was partially reversed by LM49 treatment (Figure 4H,I).

To look paralleling evidence, we assessed the role of LM49 across different cellular contexts through additional cell line rat renal TEC NRK‐52E. We noticed that LM49 had no significant effect on the viability of NRK‐52E cells within the concentration range of 0–60 μM (Figure S2A). Consistently, LM49 treatment decreased the percentage of Hoechst and PI staining double positive NRK‐52E cells and LDH release upon TNF‐α/OGSD (Figure S2B,C,E), but increased NRK‐52E cells viability (Figure S2D). Additionally, qRT‐PCR results showed that MCP‐1, IL‐1β, IL‐6 and TNF‐α were similarly elevated in the NRK‐52E cells upon TNF‐α/OGSD treatment, whereas these increases were inhibited by LM49 intervention (Figure S2F,G). Furthermore, in NRK‐52E cell line, TNF‐α/OGSD treatment induced the pathogenesis of EMT, which was also partially inhibited by LM49 treatment (Figure S2H,I). Taken together, these results suggest that LM49 can protect renal TECs from inflammation and necrosis and inhibits EMT in vitro.

### 3.5 LM49 protects TECs and inhibits EMT through blocking HMGB1 in vitro

**FIGURE 5:**
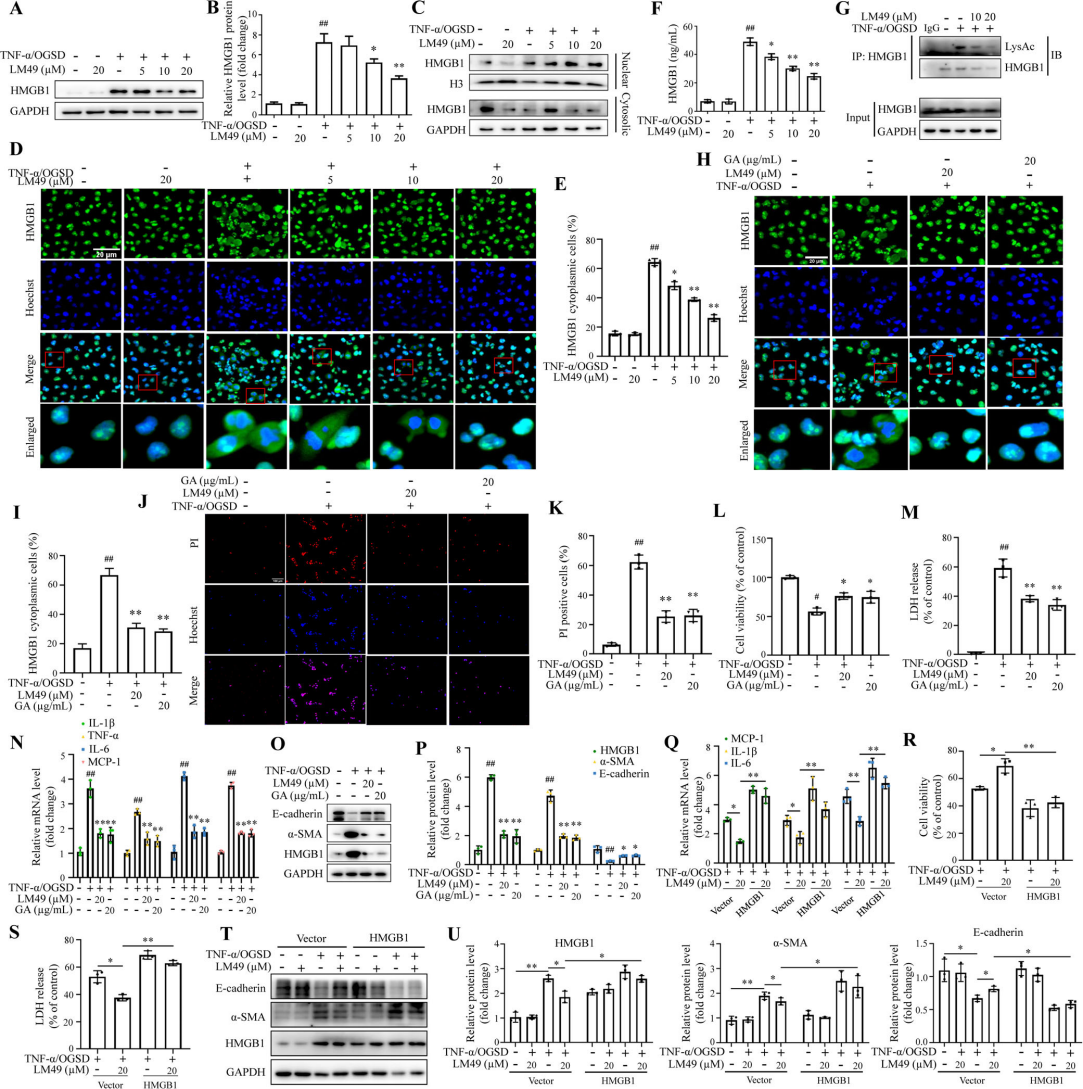
5,2′‐Dibromo‐2,4′,5′‐trihydroxydiphenylmethanone (LM49) protects tubular epithelial cells (TECs) and inhibits epithelial‐to‐mesenchymal transition through blocking high‐mobility group box 1 (HMGB1) in vitro. (A, B) Western blotting (A) and quantitative results (B) of HMGB1 in HK‐2 cells treated with LM49 in the absence or presence of tumour necrosis factor alpha for 12 h under the conditions of oxygen–glucose‐serum deprivation (TNF‐α/OGSD) for 12 h (mean ± SD, *n* = 3, ^##^ *p* < 0.01 vs. control; *p < 0.05, **p < 0.01 vs. model). (C) Western blotting results of the nuclear and cytoplasmic HMGB1 levels in HK‐2 cells treated with LM49 in the absence or presence of TNF‐α/OGSD for 12 h, *n* = 4. (D, E) Representative fluorescence images (D) and quantitative results (E) of HMGB1 in HK‐2 cells treated with LM49 in the absence or presence of TNF‐α/OGSD for 12 h. Scale bar, 20 μm, ×200. (F) ELISA analysed the levels of HMGB1 in the extracellular supernatants of HK‐2 cells treated with LM49 in the absence or presence of TNF‐α/OGSD for 12 h. *n* = 3. (G) Co‐immunoprecipitation analysis of the acetylated HMGB1 levels in HK‐2 cells treated with LM49 in the absence or presence of TNF‐α/OGSD for 12 h. (H, I) Representative immunofluorescence images (H) and quantitative results (I) of HMGB1 in HK‐2 cells treated with LM49 or HMGB1 inhibitor Glycyrrhetinic acid (GA) in the absence or presence of TNF‐α/OGSD for 12 h (mean ± SD, *n* = 3, ^##^ *p* < 0.01 vs. Control; **p < 0.01 vs. model), Scale bar, 20 μm, ×200. (J, K) Representative images (J) and quantification (K) of Hoechst/propidium iodide (PI) staining in HK‐2 cells treated with LM49 or GA in the absence or presence of TNF‐α/OGSD for 12 h (mean ± SD, *n* = 3, ^##^ *p* < 0.01 vs. control; **p < 0.01 vs. model), Scale bar, 100 μm, ×200. (L) HK‐2 cells viability in HK‐2 cells treated with LM49 or GA in the absence or presence of TNF‐α/OGSD for 12 h (mean ± SD, *n* = 3, ^#^ *p* < 0.05 vs. control; *p < 0.05 vs. model). (M) Lactate dehydrogenase (LDH) release in HK‐2 cells treated with LM49 or GA in the absence or presence of TNF‐α/OGSD for 12 h (mean ± SD, *n* = 3, ^##^ *p* < 0.01 vs. control; **p < 0.01 vs. model). (N) mRNA expression levels of IL‐1β, TNF‐α, IL‐6, and monocyte chemoattractant protein‐1 (MCP‐1) in HK‐2 cells treated with LM49 or GA in the absence or presence of TNF‐α/OGSD for 12 h (mean ± SD, *n* = 3, ^##^ *p* < 0.01 vs. control; **p < 0.01 vs. model). (O, P) Western blotting (O) and quantitative results (P) of E‐cadherin, alpha‐smooth muscle actin (α‐SMA) and HMGB1 in HK‐2 cells treated with LM49 or GA in the absence or presence of TNF‐α/OGSD for 12 h (mean ± SD, *n* = 3, ^##^ *p* < 0.01 vs. control; *p < 0.05, **p < 0.01 vs. model). (Q) mRNA expression levels of monocyte chemoattractant protein‐1 (MCP‐1), interleukin (IL)‐1β, and IL‐6 in DMSO‐ or LM49‐treated HK‐2 cells transfected with pcDNA 3.1 or pcDNA 3.1‐HMGB1 in the absence or presence of TNF‐α/OGSD for 12 h (mean ± SD, *n* = 3, *p < 0.05, **p < 0.01). (R) Cell viability in DMSO‐ or LM49‐treated HK‐2 cells transfected with pcDNA 3.1 or pcDNA 3.1‐HMGB1 in the absence or presence of TNF‐α/OGSD for 12 h (mean ± SD, *n* = 3, *p < 0.05, **p < 0.01). (S) Lactate dehydrogenase (LDH) release in DMSO‐ or LM49‐treated HK‐2 cells transfected with pcDNA 3.1 or pcDNA 3.1‐HMGB1 in the absence or presence of TNF‐α/OGSD for 12 h (mean ± SD, *n* = 3, *p < 0.05, **p < 0.01). (T, U) Western blotting (T) and quantitative results (U) of HMGB1, E‐cadherin and α‐SMA in DMSO‐ or LM49‐treated HK‐2 cells transfected with pcDNA 3.1 or pcDNA 3.1‐HMGB1 in the absence or presence of TNF‐α/OGSD for 12 h (mean ± SD, *n* = 3, *p < 0.05, **p < 0.01).

Next, we sought to investigate whether the protective effect of LM49 on TECs upon TNF‐α/OGSD is related to inhibiting HMGB1. First, we measured HMGB1 expression in vitro. In HK‐2 and NRK‐52E cell lines, the TNF‐α/OGSD treatment induced the overall HMGB1 expression rose, and the trend was dose‐dependently reversed by LM49 intervention (Figure 5A,B and Figure S3A,B). Ulteriorly, the expressions of HMGB1 in the nucleus and cytoplasm in the cells exhibited that the translocation following TNF‐α/OGSD treatment was significantly inhibited by LM49 treatment (Figure 5C and Figure S3C). Additionally, immunofluorescence staining revealed that HMGB1 was predominantly located in the nuclei of TECs in the control group, when treated with TNF‐α/OGSD, HMGB1 was remarkably expressed in the cytoplasm of cells, whereas LM49 blocked the nuclear export of HMGB1 in a dose‐dependent manner (Figure 5D,E and Figure S3E,F). Next, we quantified HMGB1 protein levels in the cell supernatant using ELISA. The findings indicated a significant increase in HMGB1 levels in HK‐2 cells induced by TNF‐α/OGSD, which were notably reduced following LM49 treatment (Figure 5F). In addition, co‐IP results further revealed that treatment with LM49 could inhibit the TNF‐α/OGSD‐induced elevated levels of acetylated HMGB1 (Figure 5G and Figure S3D), indicating that LM49 had a critical role for inhibiting the activation of HMGB1 in vitro.

Next, we evaluated the function of HMGB1 in TNF‐α/OGSD‐treated TECs. GA, a specific inhibitor of HMGB1, was administered to the cells. As expected, LM49 or GA treatment evidently decreased the expression of HMGB1 in the TNF‐α/OGSD‐induced HK‐2 cells (Figure 5O,P). Immunofluorescence staining showed that LM49 and GA inhibited the nucleocytoplasmic translocation of HMGB1 in the TNF‐α/OGSD‐induced HK‐2 cells (Figure 5H,I). The results obtained from Hoechst/PI staining, CCK‐8, and LDH assays demonstrated that GA reduced the percentage of PI positive HK‐2 cells, the decrease in cell viability, and the increase in LDH release induced by TNF‐α/OGSD, consistent with results observed using LM49 (Figure 5J–M). Moreover, the increased mRNA levels of chemokines MCP‐1 and inflammatory factors IL‐1β, TNF‐α and IL‐6 in the TNF‐α/OGSD‐induced HK‐2 cells were also downregulated by GA or LM49 intervention (Figure 5N). Furthermore, GA also suppressed the EMT process of HK‐2 cells. The elevated expression of α‐SMA and induced expression of E‐cadherin in the TNF‐α/OGSD‐induced HK‐2 cells were both reversed by GA or LM49 treatment (Figure 5O,P). To further verify the impact of HMGB1 inhibition in HK‐2 cells, we overexpressed HMGB1 in HK‐2 cells. Functionally, we found that HMGB1 overexpression abrogated the improved the mRNA expression levels of MCP‐1, IL‐1β and IL‐6 in the TNF‐α/OGSD‐induced HK‐2 cells treated using LM49 (Figure 5Q). Similarly, HMGB1 overexpression also deteriorated the cell viability and LDH release in TNF‐α/OGSD‐induced HK‐2 cells treated with LM49 (Figure 5R,S). Importantly, HMGB1 overexpression also restored the increased EMT levels, relative to that of TNF‐α/OGSD‐induced HK‐2 cells treated with LM49 only (Figure 5T,U). Taken together, these data indicate that LM49 protects HK‐2 cells and inhibits EMT by inhibiting HMGB1 upregulation.

### 3.6 LM49 inhibits the nucleocytoplasmic translocation of HMGB1 via targeting Hsp90α

**FIGURE 6:**
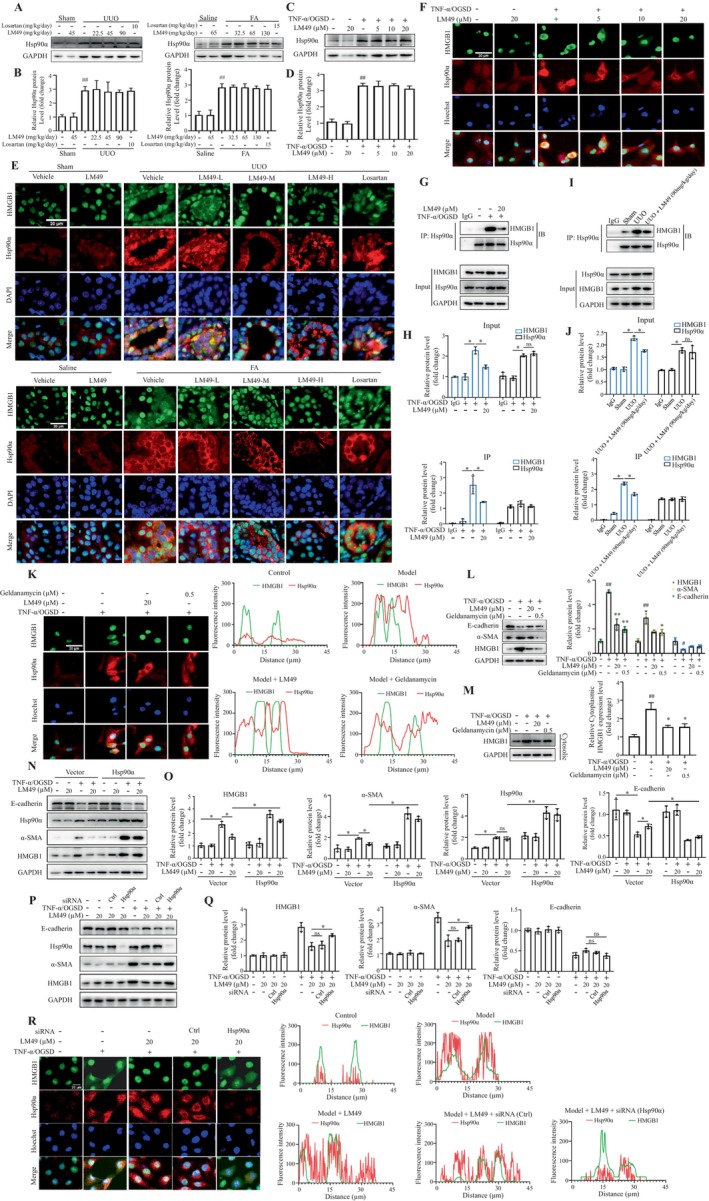
5,2′‐Dibromo‐2,4′,5′‐trihydroxydiphenylmethanone (LM49) inhibits the nucleocytoplasmic translocation of high‐mobility group box 1 (HMGB1) via targeting heat shock protein 90 alpha family class A member 1 (Hsp90α). (A) Western blotting of Hsp90α in kidneys of unilateral ureteral obstruction (UUO) rats or folic acid (FA) mice. (B) Quantitative results of Hsp90α intensity in (A) (mean ± SD, *n* = 3, ^##^ *p* < 0.01 vs. sham rats or control mice). (C) Western blotting of Hsp90α in HK‐2 cells treated with LM49 in the absence or presence of tumour necrosis factor alpha for 12 h under the conditions of oxygen–glucose‐serum deprivation (TNF‐α/OGSD) for 12 h. (D) Quantitative results of Hsp90α intensity in (C) (mean ± SD, *n* = 3, ^##^ *p* < 0.01 vs. control cell group). (E) Representative image of immunofluorescence staining double‐positive cells of Hsp90α (red) and HMGB1 (green) in the kidneys of UUO rats or FA mice. Scale bars, 50 μm. (F) Representative images of immunofluorescence staining double‐positive cells of Hsp90α (red) and HMGB1(green) in HK‐2 cells treated with LM49 in the absence or presence of TNF‐α/OGSD for 12 h. Scale bars, 20 μm. (G, H) Co‐immunoprecipitation of Hsp90α with HMGB1 (G) and quantitative results (H) of Hsp90α and HMGB1 intensity in HK‐2 cells treated with TNF‐α/OGSD in the absence or presence of LM49 for 12 h. (I, J) Co‐immunoprecipitation of Hsp90α with HMGB1 (I) and quantitative results of Hsp90α and HMGB1 intensity (J) in kidney tissues of sham, UUO and UUO + LM49 (90 mg/kg/day). Three independent experiments were performed. (K) Representative immunofluorescence images and fluorescence intensity of Hsp90α (red) and HMGB1 (green) in HK‐2 cells treated with LM49 or Hsp90α inhibitor geldanamycin in the absence or presence of TNF‐α/OGSD for 12 h, Image J software was used for statistics. Scale bars = 20 μm. *n* = 3 samples per group. (L) Western blotting and quantitative results of alpha‐smooth muscle actin (α‐SMA), E‐cadherin and HMGB1 in HK‐2 cells treated with LM49 or geldanamycin in the absence or presence of TNF‐α/OGSD for 12 h (mean ± SD, *n* = 3, ^##^ *p* < 0.01 vs. control; *p < 0.05, **p < 0.01 vs. model). (M) Western blotting and quantitative results of cytoplasmic HMGB1 in HK‐2 cells treated with LM49 or geldanamycin in the absence or presence of TNF‐α/OGSD for 12 h (mean ± SD, *n* = 3, ^##^ *p* < 0.01 vs. control; *p < 0.05 vs. model). (N, O) Immunoblot analysis (N) and quantitative results (O) of the protein expression levels of HMGB1, α‐SMA and E‐cadherin in DMSO‐ or LM49‐treated HK‐2 cells transfected with pcDNA 3.1 or pcDNA 3.1‐ Hsp90α in the absence or presence of TNF‐α/OGSD (mean ± SD, *n* = 3, *p < 0.05, **p < 0.01). (P, Q) Immunoblot analysis (P) and quantitative results (Q) of the protein expression levels of HMGB1, α‐SMA and E‐cadherin in DMSO‐ or LM49‐treated HK‐2 cells transfected with ctrl siRNA or Hsp90α siRNA in the absence or presence of TNF‐α/OGSD (mean ± SD, *n* = 3, *p < 0.05). (R) Representative fluorescence images and fluorescence intensity of Hsp90α (red) and HMGB1 (green) in HK‐2 cells treated with ctrl siRNA or Hsp90α siRNA. Scale bar, 20 μm, ×200.

To delve deeper into the potential target of LM49, the structural data file of LM49 was submitted to Pharmmapper using the ‘All Targets’ Model. Subsequently, the top 300 potential targets were listed based on the normalized fit score in descending order (Table S2). Combined with the scoring results of the molecular docking, we ultimately noticed the target protein Hsp90α, which has been reported to be involved in fibrogenesis in various tissues.^22^, ^25^, ^26^, ^50^, ^51^, ^52^ Most importantly, a recent study reported that Hsp90α could regulate the localization of HMGB1.^26^ Hence, to understand whether and how the mechanism pattern between Hsp90α and HMGB1 is involved in UUO‐ and FA‐induced fibrogenesis, we first characterized the expression of Hsp90α in vivo and in vitro. The immunoblotting data revealed that Hsp90α was significantly increased in fibrotic kidneys (Figure 6A,B). Surprisingly, there was no substantial difference in the levels of Hsp90α expression after LM49 intervention (Figure 6A,B). In line with in vivo results, LM49 did not significantly influence the total protein expression levels of Hsp90α in the TNF‐α/OGSD‐induced HK‐2 cells (Figure 6C,D). To elucidate the potential role of Hsp90α in the pathogenesis of renal fibrosis, co‐immunofluorescence with Hsp90α (red) and HMGB1 (green) revealed that little Hsp90α was detected in the kidneys of sham rats or control mice, and HMGB1 was mainly located in TECs nuclei (Figure 6E). However, after induction by UUO or FA, Hsp90α was highly expressed and distributed in both the nucleus and cytoplasm (Figure 6E). Meanwhile, HMGB1 was transferred extensively from the nucleus to the cytoplasm, presenting as significantly increased areas of co‐location of Hsp90α and HMGB1 in the cytoplasm (Figure 6E), which indicated that the increase in the binding of both, followed by translocation from nucleus to cytoplasm occurred in fibrotic kidneys. Notably, LM49 administration significantly inhibited the binding of Hsp90α and HMGB1 and subsequent diffusion of HMGB1 to the cytoplasm (Figure 6E). To obtain parallel evidence, we performed co‐immunofluorescence analysis in HK‐2 cells. Compared to control cells, Hsp90α was markedly increased after treatment with TNF‐α/OGSD (Figure 6F). Meanwhile, we found that in TNF‐α/OGSD‐induced HK‐2 cells, HMGB1 bound to Hsp90α and translocated to the cytoplasm and both processes were markedly inhibited by LM49 intervention (Figure 6F). To further investigate the effects of LM49 on the binding of Hsp90α and HMGB1, co‐IP assay confirmed the endogenous binding of HMGB1 to Hsp90α was barely detected in the control HK‐2 cells and the kidney tissues of sham operated rats, whereas the interaction between HMGB1 and Hsp90α was predominantly noticed in the TNF‐α/OGSD‐induced HK‐2 cells and the kidney tissues of UUO rats (Figure 6G–J). As expected, administration of LM49 significantly reduced the binding of Hsp90α and HMGB1 (Figure 6G–J).

To further determine whether LM49 hindered the movement of HMGB1 from the nucleus to the cytoplasm facilitated by Hsp90α, the Hsp90α inhibitor geldanamycin was used. Geldanamycin treatment decreased the total protein expression and prevented the translocation of HMGB1 from the nucleus to the cytoplasm, as demonstrated by co‐immunofluorescence and immunoblotting analyses, respectively, consistent with results observed for LM49 (Figure 6K–M). Meanwhile, geldanamycin decreased α‐SMA levels and increased E‐cadherin levels in TNF‐α/OGSD‐induced HK‐2 cells (Figure 6L). To further validate the involvement of Hsp90α in LM49‐induced reduction and relocation of HMGB1 protein, we overexpressed Hsp90α in HK‐2 cells. Upon transfection with the pcDNA3.1 vector control, LM49 treatment led to a decrease in HMGB1 expression and α‐SMA levels, along with an increase in the levels of E‐cadherin induced by TNF‐α/OGSD. However, these effects were antagonized by the overexpression of Hsp90α, which reverted back to those observed with TNF‐α/OGSD treatment only (Figure 6N,O). To further elucidate LM49 suppresses HMGB1 expression and its nucleocytoplasmic translocation in an Hsp90α‐dependent manner, we next transduced with small interfering RNA for the knockdown of Hsp90α in the LM49‐treated HK‐2 cells. Functionally, we found that Hsp90α silencing abrogated the reduced expression of HMGB1 and EMT in the LM49‐treated HK‐2 cells (Figure 6P–R). In addition, Hsp90α silencing also partially restored the nucleocytoplasmic translocation levels of HMGB1 in the LM49‐treated HK‐2 cells (Figure 6R). Taken together, our results suggested that LM49 reduced HMGB1 translocation from the nucleus to the cytoplasm in an Hsp90α‐dependent manner.

### 3.7 LM49 bound directly to Hsp90α

**FIGURE 7:**
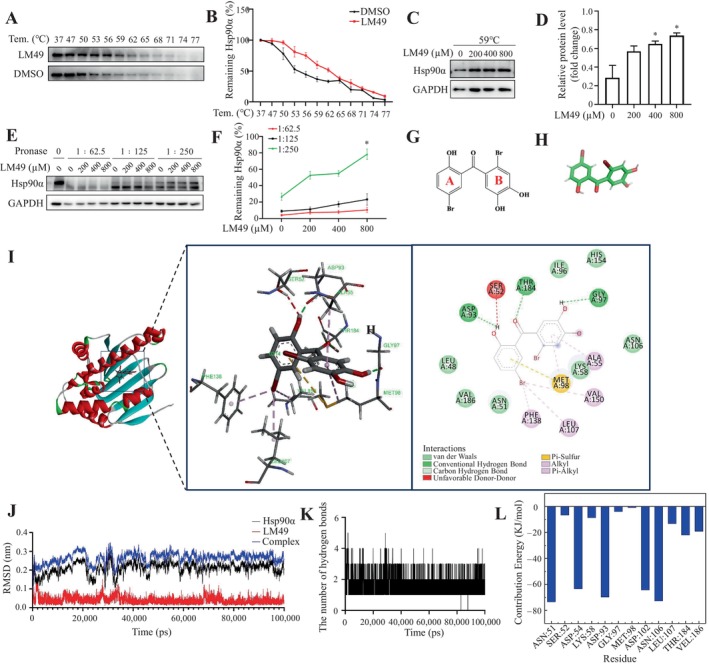
5,2′‐Dibromo‐2,4′,5′‐trihydroxydiphenylmethanone (LM49) bound directly to heat shock protein 90 alpha family class A member 1 (Hsp90α). (A) Cellular thermal shift (CETSA) confirms the direct binding of LM49 to Hsp90α in various temperatures. (B) Quantification of Hsp90α intensity in (A). Each group was normalized as a percentage of that at 37°C. (*n* = 3). (C) CETSA confirms the direct binding of LM49 to Hsp90α in the presence of increasing concentrations of LM49. (D) Quantification of Hsp90α intensity in (C). *p < 0.05 versus 0 μM group (*n* = 3). (E) Drug affinity responsive target stability confirms the direct binding of LM49 to Hsp90α. (F) Quantification of Hsp90α intensity in (E). *p < 0.05 versus 0 μM group (*n* = 3). (G, H) 2d chemical structure and 3d interactive chemical structure of LM49. (I) Molecular docking binding mode and residues detail of LM49 and Hsp90α analysed with Autodock4.2. (J) Root mean square deviation (RMSD) of LM49, Hsp90α and LM49–Hsp90α complex obtained during the 100 ns MD simulation. (K) Hydrogen bond map of LM49 and Hsp90α during the 100 ns molecular dynamics simulation. (L) Energy decomposition of binding free energy of LM49–Hsp90α complex.

Cellular thermal shift and drug affinity responsive target stability assays are label‐free techniques applied to assess the interaction of small molecules with potential protein targets. The principle is that the protein after binding the ligand shows enhanced stability and protection during protease hydrolysis and heat treatments. Therefore, to additionally validate the interaction between LM49 and Hsp90α, we conducted drug affinity responsive target stability and cellular thermal shift experiments.^53^ As the temperature increased, particularly within the range of 53–59°C, the rate of Hsp90α disappearance was observed to be slower in the LM49‐treated group compared to the DMSO‐treated group (Figure 7A,B). Hsp90α degradation decreased at 59°C, as LM49 concentration increased (Figure 7C,D). Moreover, we observed that Hsp90α was nearly completely shielded from degradation when using a protease‐to‐cell lysate ratio of 1:250 in the presence of 800 μM LM49 (Figure 7E,F).

To gain further insight into the molecular‐level binding mode between LM49 and Hsp90α, we used SYBYL‐X 2.0 (Tripos, St. Louis, USA) (PDB ID: 3BMY) to dock LM49 into the N‐terminal ATP pocket of Hsp90α (Figure 7G–I). As shown in Figure 7I, the H atom on the hydroxyl group of ‘B’ ring, the H atom on the hydroxyl group of ‘A’ ring, and the carbonyl groups on the skeleton of benzophenone in the structure of benzophenone of LM49 formed hydrogen bonds with Gly97, Asp93 and Thr184, respectively. The ‘B’ ring and the ‘A’ ring of LM49 formed pi‐sulphur interaction and pi‐alkyl interactions, respectively, with Met98. The bromine atoms on the ‘A’ ring of LM49 formed pi‐alkyl interactions with Phe138, Leu107 and Val150 and those on the ‘B’ ring formed pi‐alkyl interactions with Ala55. The other residues in the binding pocket showed van der Waals interactions with the hydrophobic alkyl chain of LM49. Taken together, these findings indicate that LM49 occupied the hydrophobic pocket responsible for ATP binding, with hydrogen bonds and van der Waals forces serving as the primary binding forces in the LM49‐Hsp90α complex.

Moreover, molecular dynamics (MD) simulation was further carried out to assess the stability of the structure and the hydrogen bond interactions between LM49 and Hsp90α. The Hsp90α‐LM49 complex was equilibrated after 100 ns MD simulations to obtain the plot of root mean square deviation (in ångstrom) (Figure 7J). root mean square deviation provides information on protein backbone stability. The data indicated that the systems were basically stable without significant variations throughout the entire 100 ns simulation run (Figure 7J). Next, we conducted hydrogen bonding analysis to investigate the interaction between Hsp90α and LM49. As shown in Figure 7K, the distribution of the average number of hydrogen bonds between Hsp90α and LM49 was recorded at 1.5, indicating a strong hydrogen bond interaction between them. Moreover, the gmx_Mmpbsa script (https://jerkwin.github.io/gmxtool/) was utilized to calculate the binding energies of protein‐ligand complexes at equilibrium. The binding energy results between Hsp90α and LM49, calculated using MM/PBSA methods, are presented in Table 1. In the Hsp90α‐LM49 complex system, the binding free energy between them was −30.77 kcal/mol, indicating a stable binding between them. Moreover, we conducted an energy decomposition of the binding free energy. As shown in Figure 7L, ASN51, ASP54, ASP93, ASP102, ASP102 and ASN106 were the main amino acids involved in the interaction between LM49 and Hsp90α. Taken together, these results indicated that LM49 can be combined with Hsp90α through direct interactions, forming a stable complex.

**TABLE 1:** Protein ligand MMPBSA analysis.

Energy	Complex
ΔVDWAALS (Kcal/mol)	−29.30
ΔEEL (Kcal/mol)	−34.68
ΔEGB (Kcal/mol)	37.51
ΔESURF (Kcal/mol)	−4.31
ΔGGAS (Kcal/mol)	−63.98
ΔGSOLV (Kcal/mol)	33.21
ΔTOTAL (Kcal/mol)	−30.77

Abbreviations: ΔEEL, electrostatic energy; ΔEGB, polar solvation energy; ΔESURF, non‐polar solvation energy; ΔGGAS, molecular mechanics energy (meteorological energy); ΔGSOLV, solvation energy; ΔVDWAALS, van der Waals energy.

## 4 DISCUSSION

CKD is marked by the gradual advancement of renal fibrosis and a gradual decline in glomerular filtration rate, ultimately resulting in end‐stage renal disease. The global prevalence and incidence of CKD are increasing rapidly.^54^ Renal fibrosis, marked by TEC death, ECM accumulation, and inflammatory response, is a common sign of progressive CKD.^55^ Thus, targeting these pathophysiological mechanisms may mitigate and prevent renal fibrosis and CKD progression. LM49, a polyphenol derivative synthesized by our group with excellent anti‐inflammatory properties, has been exhibited to mitigate excessive ECM deposition in the kidneys of rats with diabetic nephropathy.^31^, ^32^, ^33^, ^34^ However, the LM49 protective activity against renal fibrosis and the exact molecular mechanisms remain largely unknown. In this study, we presented the first evidence that LM49 administration effectively decreased UUO‐ and FA‐induced renal fibrosis and improved filtration dysfunction and renal pathological injuries. Importantly, we found that LM49 remarkably inhibited HMGB1 nuclear–cytoplasmic translocation and release and alleviated the inflammatory response and cell necrosis, both in vivo and in vitro. Mechanistically, we proved that the loss of HMGB1 substantially impaired the protective effects of LM49 against renal necroinflammation and fibrosis, indicating that HMGB1 is a pivotal mediator in LM49 therapy against renal injury. Moreover, we found that LM49 directly bound to Hsp90α and effectively reduced the interaction of Hsp90α with HMGB1, thereby further blocked the translocation and activation of HMGB1.

Non‐microbial (sterile) inflammation is widely considered a major factor in the CKD development.^56^ Prior research has suggested that renal fibrosis is distinguished by the recruitment of inflammatory cells, infiltration of macrophages and elevated levels of pro‐inflammatory cytokines.^56^ LM49 has shown excellent anti‐inflammatory activity in animal models of various inflammatory diseases, such as diabetic nephropathy, acute pyelonephritis and LPS‐induced acute liver injury in the previous studies.^31^, ^32^, ^34^, ^57^ In this study, our data demonstrated that LM49 alleviated inflammatory infiltration in fibrotic kidneys induced by UUO and FA, as demonstrated by a reduction in total leukocyte recruitment and macrophage infiltration. In addition, our study demonstrated that LM49 treatment decreased UUO‐ and FA‐induced increases in MCP‐1, a chemotactic cytokine released by TECs in response to renal damage, triggering the influx of inflammatory cells.^58^ In conjunction with the activation of immune cells within the kidney, these recruitments subsequently resulted in heightened production of pro‐inflammatory cytokines, including TNFα, IL‐6, IL‐1β and MCP‐1.^59^ Consistently, we observed that the fibrotic tissues and TNF‐α/OGSD‐induced HK‐2 cells both suffered raised pro‐inflammatory cytokine levels. However, LM49 administration significantly alleviated these effects. Moreover, RNA sequencing of the renal tissue indicated that the inflammatory response in fibrotic kidneys induced by UUO and FA played a very important driving role, and the administration of LM49 improved the excessive inflammatory response in fibrotic kidneys, which is consistent with our prior researches. Interestingly, recent studies have shown that resveratrol, curcumin and other polyphenol compounds exhibit renal protective effects by preventing the release of inflammatory molecules and reducing ECM deposition during the initiation and activation stages of renal fibrosis.^34^ This further supports the potential anti‐inflammatory and anti‐fibrotic properties of LM49 in fibrotic kidneys.

In fibrotic kidneys, tubular cell death is also believed to trigger tubular loss and tubulointerstitial atrophy, thereby promoting the advancement of interstitial inflammation and fibrosis.^60^, ^61^ In recent years, there has been a growing understanding of the molecular pathways linking cell death to inflammation and vice versa. These processes have been termed ‘necroinflammation’ and are mutually reinforced in an auto amplification loop. Accumulating evidence indicates that necroinflammation plays an essential role of extensive participate in CKD.^12^, ^42^, ^62^ We propose that LM49 mitigates renal fibrosis by inhibiting necroinflammation induced by UUO and FA. We found that UUO and FA increased TECs necrosis, which was accompanied by the upregulation of TUNEL‐positive TECs in fibrotic kidneys and visible necrotic morphology of renal TECs, as shown by TEM. However, these effects were all reversed by LM49 intervention. These findings are consistent with the results of prior studies, which suggested that the pharmacological blockade of necroinflammation attenuated UUO‐induced inflammation and fibrosis.^9^, ^10^, ^11^ In the current investigation, RNA sequencing results also revealed an enrichment of cell necrosis processes in fibrotic kidneys induced by UUO and FA, indicating that a reduction in necrosis and inflammation could ameliorate renal damage and fibrosis. Notably, RNA sequencing results revealed that DEGs were also enriched in cell necrosis processes after LM49 intervention. Further differential gene heatmaps related to inflammation and necrosis revealed that LM49 significantly improved UUO‐ and FA‐induced inflammation and necrosis in the fibrotic kidney, providing definite evidence that LM49 has protective effects against renal cell necrosis and is involved in the clearance of tissue inflammation.

The mechanism by which LM49 treatment abrogates renal necroinflammation and fibrosis in the UUO and FA models remains unclear. It is well accepted that modes of regulated necrosis can directly cause kidney injury or recruit immune cells and stimulate inflammatory responses through the release of cytokines and DAMPs.^55^ HMGB1, a significant DAMP, belongs to the high‐mobility group nuclear protein family and is among the most evolutionarily conserved proteins. Leelahavanichkul et al.^63^ demonstrated that HMGB1 is released from necrotic cells and that anti‐HMGB1 treatment could alleviate CKD‐sepsis in a model of progressive CKD mice with 5/6 nephrectomy. More importantly, a recent study showed that human rHMGB1 intervention induces alterations in epithelial morphology, consistent with EMT.^64^ In this research, we found a significant increase in total HMGB1 expression and nuclear–cytoplasmic translocation, as well as elevated levels of HMGB1 in the circulatory system of fibrotic kidneys, all of which were inhibited by LM49 treatment. This phenomenon potentially elucidates the consistent presence of HMGB1 within the nucleus of mammalian cells, followed by its subsequent release into the extracellular environment upon exposure to diverse stimuli. This process is believed to be influenced by necroinflammation, which plays a critical role.^55^ Furthermore, upon release from activated or necrotic cells, HMGB1 acts as a powerful pro‐inflammatory cytokine that exerts its effects through various cell‐surface receptors, including receptor for advanced glycation end products, TLR2, TLR4 and TLR9.^65^ Similarly, our data showed that after LM49 administration, these receptors downregulated, consistent with the recognition that DAMPs trigger sterile inflammation, which makes them promising targets for developing and improving diagnostic and therapeutic approaches for renal fibrosis.^15^ RNA sequencing analysis of DEGs related to inflammation and necrosis further confirmed that HMGB1 was significantly elevated in fibrotic kidneys induced by UUO and FA treatment, whereas LM49 treatment significantly reduced HMGB1 expression levels. In addition, we investigated whether stimulated HMGB1 is critical for the anti‐necroinflammation and anti‐fibrotic activities of LM49. As expected, the protective effect of LM49 in the fibrotic model was consistent with those for HMGB1 inhibitor GA in TNF‐α/OGSD‐induced HK‐2 cells. In addition, the genetic overexpression of HMGB1 remarkably blocked the action of LM49 on TNF‐α/OGSD‐induced cell necrosis, inflammatory response and fibrosis.

Hsp90α, Hsp90β, the glucose regulated protein 94 and the tumour necrosis factor receptor‐associated protein‐1 are four isoforms of Hsp90 in cells involved in various cellular functions.^19^ Previous studies have found that activated Hsp90α was elevated in CKD human and animal kidneys, which is linked to the severity of renal fibrosis and ECM accumulation.^25^, ^50^, ^51^ Our data verified that the expression levels of Hsp90α in fibrotic kidneys and TNF‐α/OGSD‐induced HK‐2 cells were indeed elevated; however, LM49 does not affect the total expression levels of Hsp90α. To explore the potential evidence, it has been reported that Hsp90 possesses three conservative domains: a N‐terminal domain (NTD), middle domain (M‐domain) and C‐terminal domain which are highly conserved during evolution.^19^ The ATP‐binding site within the NTD plays a key role in facilitating the interaction between co‐chaperones and clients through ATPase activity, enabling their combination. The M‐domain contains a site for ATP hydrolysis and binding to the client, whereas the C‐terminal domain is responsible for dimerization. Dimerization takes place after Hsp90 binds to ATP and the client binds to the M‐domain, leading to a change in configuration of the NTD from open to closed.^19^ Furthermore, the interaction between the client and Hsp90, as a crucial mediator, is influenced by the modification of Hsp90 and its co‐chaperone.^19^ The Hsp90/co‐chaperone/client complexes are emerging targets for diseases treatment. Therefore, blocking the interactions between Hsp90 and its clients has been investigated, and inhibitors have proven to be the most effective approach.^66^ Importantly, we ascertained the LM49, synthesized by our research team, as a novel Hsp90α inhibitors in the present study. In this study, dual immunofluorescence staining and co‐IP first indicated that LM49 significantly inhibited the co‐localization and binding of Hsp90 and HMGB1. Next, molecular docking and MD simulation demonstrated that LM49 binds to the N‐terminal ATP pocket of Hsp90α, thereby inhibiting the role of Hsp90α in promoting HMGB1 nuclear export and alleviating HMGB1‐mediated inflammation and necrosis. The compared results using Hsp90α inhibitor geldanamycin also supported this conclusion. Furthermore, the overexpression of Hsp90α antagonizes the inhibitory impact of LM49 on HMGB1 and the EMT process of TNF‐α/OGSD‐induced HK‐2 cells.

## 5 CONCLUSIONS

**FIGURE 8:**
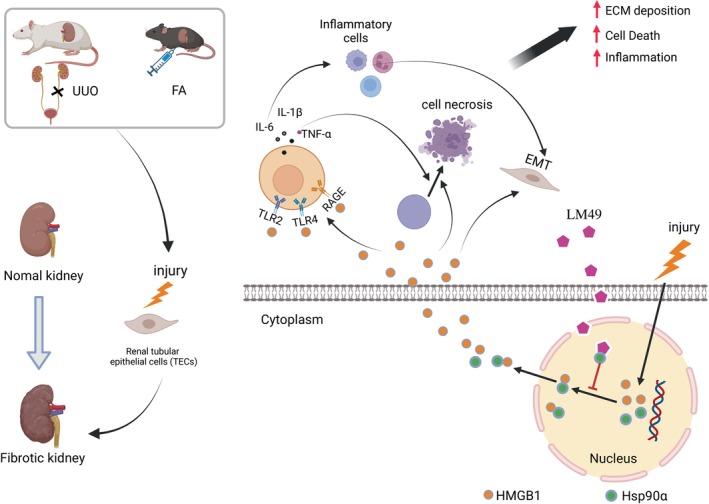
The mechanism of 5,2′‐Dibromo‐2,4′,5′‐trihydroxydiphenylmethanone (LM49) direct binding to heat shock protein 90 alpha family class A member 1 (Hsp90α) attenuates the high‐mobility group box 1 (HMGB1) nuclear translocation mediated renal inflammation and fibrosis. ECM, extracellular matrix; EMT, epithelial‐to‐mesenchymal transition; FA, folic acid; IL, interleukin; RAGE, receptor for advanced glycation end products; TLR, Toll‐like receptor; TNF, tumour necrosis factor; UUO, unilateral ureteral obstruction.

Our findings indicate that LM49 can effectively ameliorate UUO‐ and FA‐induced renal fibrosis, which is connected to the suppression of HMGB1‐induced inflammation and TEC necrosis (Figure 8). We found that LM49 targets the N‐terminal ATP pocket of Hsp90α and reduces the interaction of Hsp90α with HMGB1, thereby further blocking HMGB1 nuclear–cytoplasmic translocation and activation. These observations provide fresh perspectives on the novel pathogenic mechanisms underlying renal fibrosis and provide theoretical evidence supporting the application of LM49 in the prevention and treatment of CKD.

## AUTHOR CONTRIBUTIONS

Huizhi Wei performed the research, analysed the data and wrote the manuscript. Jinhong Ren and Fan Yang provided the support for the experimental design. Jinhong Ren and Chengxiao Zhao helped guide the experimental operation. Hongxia Yuan and Yuanlin Zhang helped visualize and analyse images. Xiue Feng synthesized LM49. Qingshan Li and Fan Yang designed the study, reviewed, edited and revised the manuscript.

## CONFLICT OF INTEREST STATEMENT

The authors declare no conflicts of interest.

## Supporting information


**Data S1.** Supporting information.

## Data Availability

The data that support the findings of this study are available on request from the corresponding author. The data are not publicly available due to privacy or ethical restrictions.
