# Supplementary material for: Targeting Hsp90α to inhibit HMGB1‐mediated renal inflammation and fibrosis
Source: Cell Prolif. 2024 Nov 20;58(3):e13774. doi: 10.1111/cpr.13774 (PMC11882747; doi:10.1111/cpr.13774)
Supplement: Supplementary file 1 — Data S1. Supporting information. [file CPR-58-e13774-s001.docx]

**
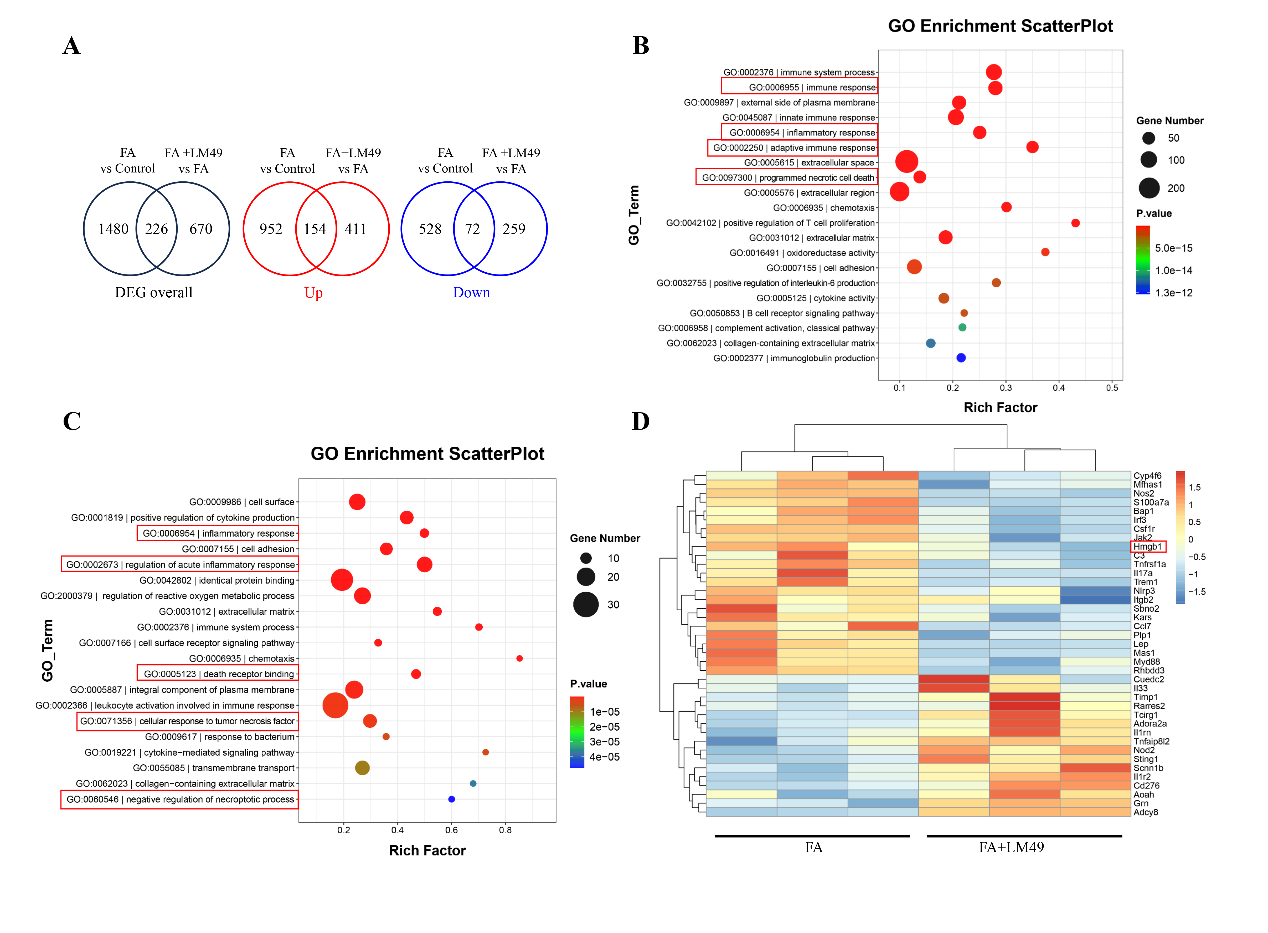
**

**Supplementary Fig. 1. Transcriptomic analysis of renal tissue in FA-induced mice. A** Venn diagram suggesting the number of differentially expressed genes, the number of upregulated genes and downregulated genes in kidneys from mice (control mice, LM49-treated FA mice) compared with FA mice. **B** Gene Ontology (GO) analysis of the differential genes in kidneys from FA mice versus control mice. **C** Gene Ontology (GO) analysis of the differential genes in kidneys from FA mice versus LM49-treated FA mice. **D** Heat map of differentially expressed genes related to inflammation and necrosis in FA mice versus LM49-treated FA mice.

**
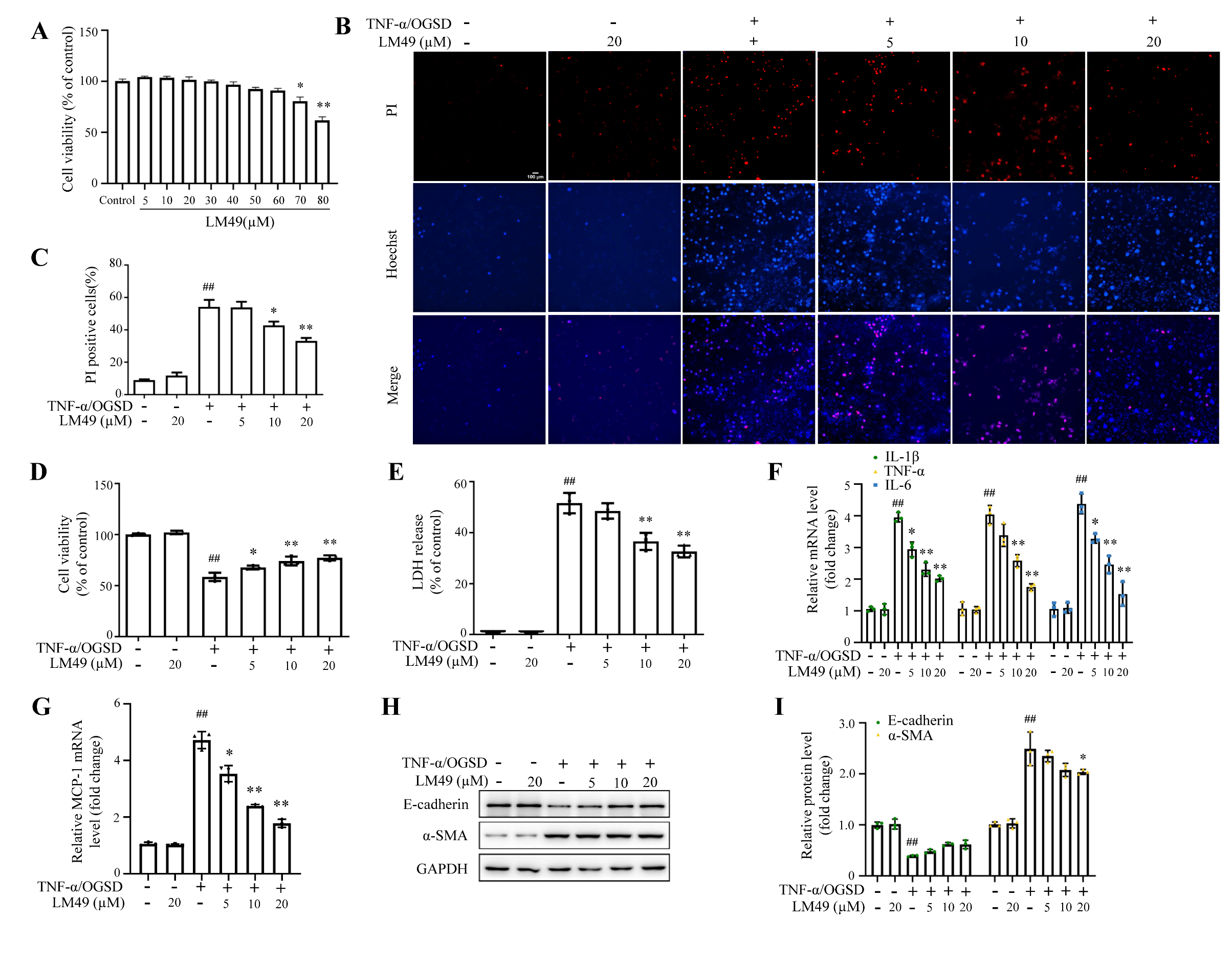
**

**Supplementary Fig. 2. *LM49 protects NRK-52E cells treated with TNF-α/OGSD against necroinflammation and EMT in vitro.* A** Effect of LM49 alone on NRK-52E cells viability. (mean ± SD, n=3, ^*^P<0.05, ^**^P<0.01 *vs*. control). **B, C** Representative images (**B**) and quantification (**C**) of Hoechst/PI staining, Scale bars, 100 μm, (mean ± SD, n=3, ^##^P<0.01 *vs*. control; ^**^P<0.01 *vs*. model). **D** NRK-52E cells viability treated with LM49 in the absence or presence of 20 ng/mL TNF-α for 12 h under TNF-α/OGSD (mean ± SD, n=3, ^##^P<0.01 *vs*. control; ^*^P<0.05, ^**^P<0.01 *vs*. model). **E** LDH release of NRK-52E cells treated with LM49 in the absence or presence of TNF-α/OGSD for 12 h (mean ± SD, n=3, ^##^P<0.01 *vs*. control; ^**^P<0.01 *vs*. model). **F, G** mRNA expression levels of IL-1β, TNF-α, and IL-6 **(F)** and MCP-1 **(G)** in NRK-52E cells treated with LM49 in the absence or presence of TNF-α/OGSD for 12 h (mean ± SD, n=3, ^##^P<0.01 *vs*. control; ^*^P<0.05, ^**^P<0.01 *vs*. model). **H, I** Western blotting (**H**) and quantitative results (**I**) of E-cadherin and α-SMA in the NRK-52E cells treated with LM49 in the absence or presence of TNF-α/OGSD for 12 h (mean ± SD, n=3, ^##^P<0.01 *vs*. control; ^*^P<0.05 *vs*. model).


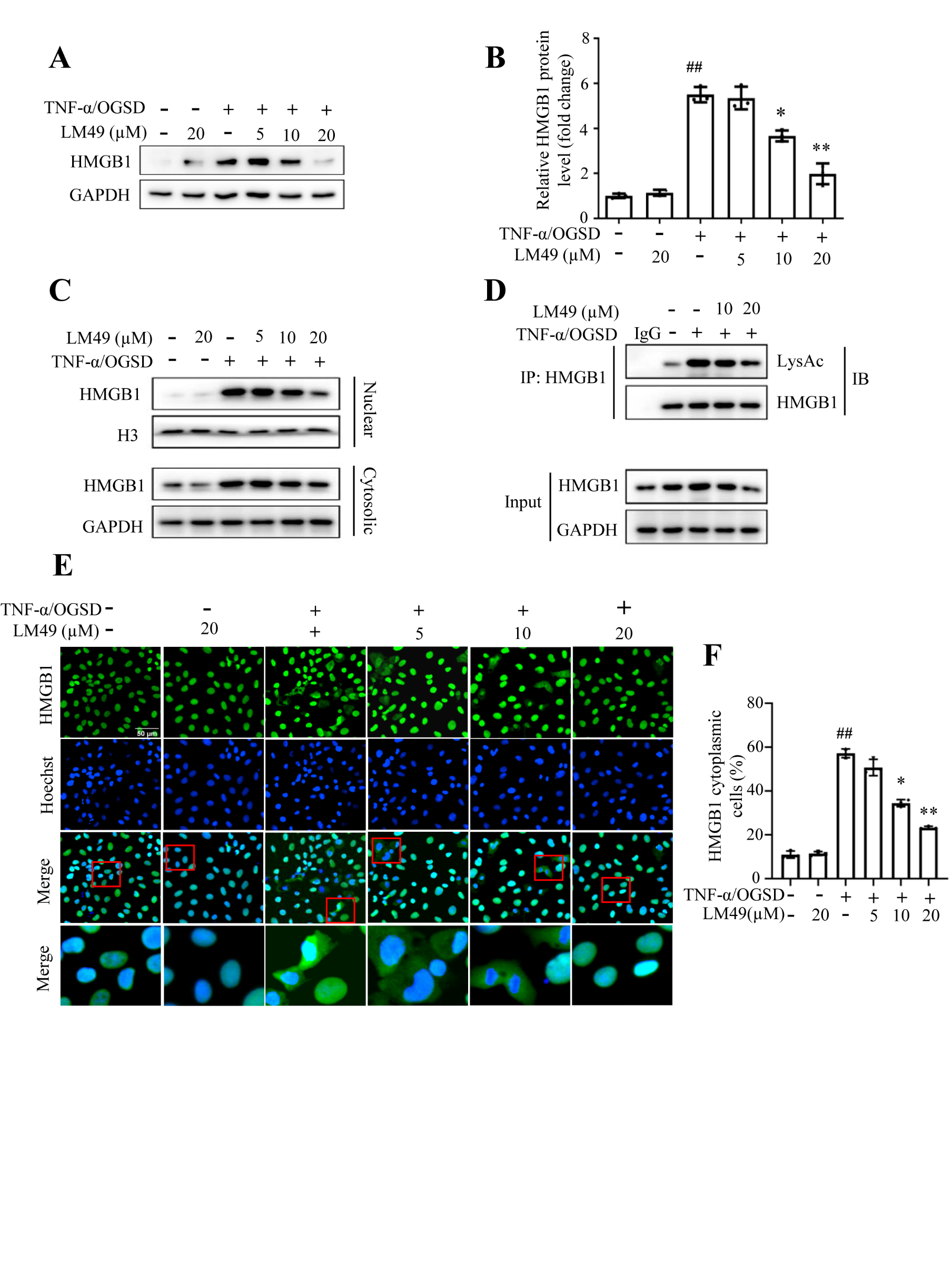


**Supplementary Fig. 3. *LM49*** ***inhibits the expression levels and nuclear cytoplasmic translation of HMGB1 in NRK-52E cells.* A, B** Western blotting (**A**) and quantitative results (**B**) of HMGB1 in NRK-52E cells treated with LM49 in the absence or presence of TNF-α/OGSD for 12 h (mean ± SD, n=3, ^##^P<0.01 *vs*. control; ^*^P<0.05, ^**^P<0.01 *vs*. model). **C** Western blotting results of the nuclear and cytoplasmic HMGB1 levels in NRK-52E cells treated with LM49 in the absence or presence of TNF-α/OGSD for 12 h, n=4. **D** Co-immunoprecipitation analysis of the acetylated HMGB1 levels in NRK-52E cells treated with LM49 in the absence or presence of TNF-α/OGSD for 12 h. **E, F** Representative fluorescence images (**E**) and quantitative results (**F**) of HMGB1 in NRK-52E cells treated with LM49 in the absence or presence of TNF-α/OGSD for 12 h. Scale bar, 20 μm, ×200.

**Supplementary Table 1 The primers of target genes**

| Gene |  | Primers |
| --- | --- | --- |
| Rat MCP-1 | Forward | CTCACCTGCTGCTACTCATTCACTG |
|  | Reverse | CTTCTTTGGGACACCTGCTGCTG |
| Rat IL-1β | Forward | CCTTGTGCAAGTGTCTGAAGC |
|  | Reverse | CCCAAGTCAAGGGCTTGGAA |
| Rat IL-6 | Forward | ACTTCCAGCCAGTTGCCTTCTTG |
|  | Reverse | TGGTCTGTTGTGGGTGGTATCCTC |
| Rat TNF-α | Forward | AAAGGACACCATGAGCACGGAAAG |
|  | Reverse | CGCCACGAGCAGGAATGAGAAG |
| Rat TLR2 | Forward | TCACTGTTCTCCAATCTCACAA |
|  | Reverse | CAGCCCAGCAAAATCTATTCTC |
| Rat TLR4 | Forward | TATCGGTGGTCAGTGTGCTT |
|  | Reverse | CTCGTTTCTCACCCAGTCCT |
| Rat RAGE | Forward | AGAAACCGGTGATGAAGGAC |
|  | Reverse | TCGAGTCTGGGTTGTCGTTT |
| mouse MCP-1 | Forward | CTGTTCACAGTTGCCGGCTG |
|  | Reverse | AGCTTCTTTGGGACACCTGCT |
| mouse IL-1β | Forward | AAATCTCGCAGCAGCACATCAA |
|  | Reverse | CCACGGGAAAGACACAGGTAGC |
| mouse IL-6 | Forward | ATCCAGTTGCCTTCTTGGGACTGA |
|  | Reverse | TAAGCCTCCGACTTGTGAAGTGGT |
| mouse TNF-α | Forward | GACAAGGCTGCCCCGACTACG |
|  | Reverse | CTTGGGGCAGGGGCTCTTGAC |
| mouse TLR2 | Forward | CTCCTGAAGCTGTTGCGTTAC |
|  | Reverse | TACTTTACCCAGCTCGCTCACTAC |
| mouse TLR4 | Forward | CGCTTTCACCTCTGCCTTCACTACAG |
|  | Reverse | ACACTACCACAATAACCTTCCGGCTC |
| mouse RAGE | Forward | GCT GTA GCT GGT GGT CAG AAC A |
|  | Reverse | CCC CTT ACA GCT TAG CAC AAG TG |
| Human MCP-1 | Forward | CAAACTGAAGCTCGCACTCTCGCC |
|  | Reverse | ATTCTTGGGTTGTGGAGTGAGTGTTCA |
| Human IL-1β | Forward | ATGGCAGAAGTACCTAAGCTCGC |
|  | Reverse | ACACAAATTGCATGGTGAAGTCAGTT |
| Human IL-6 | Forward | ATGAACTCCTTCTCCACAAGCGC |
|  | Reverse | GAAGAGCCCTCAGGCTGGACTG |
| Human TNF-α | Forward | CCACGCTCTTCTGTCTACTGAACTTC |
|  | Reverse | TGGGCTACGGGCTTGTCACTC |

**Supplementary Table 2 Pharmmapper website filter results**

| Pharma Model | Num Feature | Fit | Norm Fit | zscore | Num Hydrophobic | Num HB Acceptor | Num HB Donor | Num Positive | Num Negative | Num Aromatic | Name | Class | Uniplot | Function |
| --- | --- | --- | --- | --- | --- | --- | --- | --- | --- | --- | --- | --- | --- | --- |
| 2pg2_v | 3 | 2.993 | 0.9977 | 0.482984 | 3 | 0 | 0 | 0 | 0 | 0 | Kinesin-like protein KIF11 | MOTOR PROTEIN, CELL CYCLE | KIF11_HUMAN | Motor protein required for establishing a bipolar spindle. Blocking of KIF11 prevents centrosome migration and arrest cells in mitosis with monoastral microtubule arrays. |
| 1shj_v | 3 | 2.971 | 0.9902 | 0.30049 | 3 | 0 | 0 | 0 | 0 | 0 | Caspase-7 | HYDROLASE | CASP7_HUMAN | Involved in the activation cascade of caspases responsible for apoptosis execution. Cleaves and activates sterol regulatory element binding proteins (SREBPs). Proteolytically cleaves poly(ADP-ribose) polymerase (PARP) at a 216-Asp- |
| 1w7h_v | 3 | 2.961 | 0.9871 | 0.564863 | 2 | 0 | 1 | 0 | 0 | 0 | Mitogen-activated protein kinase 14 | NONE | Q16539 | Involved in MAP kinase activity |
| 2oji_v | 3 | 2.957 | 0.9855 | 0.541878 | 2 | 1 | 0 | 0 | 0 | 0 | Mitogen-activated protein kinase 1 | TRANSFERASE | MK01_HUMAN | Involved in both the initiation and regulation of meiosis, mitosis, and postmitotic functions in differentiated cells by phosphorylating a number of transcription factors such as ELK1. Phosphorylates EIF4EBP1; required for initiation of translation. Phosp |
| 1w8l_v | 3 | 2.944 | 0.9812 | 0.303493 | 2 | 1 | 0 | 0 | 0 | 0 | Peptidyl-prolyl cis-trans isomerase A | NONE | P62937 | Posttranslational modification, protein turnover, chaperones |
| 3dej_v | 3 | 2.942 | 0.9807 | 0.482702 | 2 | 1 | 0 | 0 | 0 | 0 | Caspase-3 | HYDROLASE, APOPTOSIS | CASP3_HUMAN | Involved in the activation cascade of caspases responsible for apoptosis execution. At the onset of apoptosis it proteolytically cleaves poly(ADP-ribose) polymerase (PARP) at a 216-Asp- |
| 3exo_v | 3 | 2.936 | 0.9788 | 0.455438 | 2 | 0 | 1 | 0 | 0 | 0 | Beta-secretase 1 | NONE | P56817 | Involved in aspartic-type signal peptidase activity |
| 3bmy_v | 4 | 3.902 | 0.9755 | 2.95633 | 2 | 1 | 1 | 0 | 0 | 0 | Heat shock protein HSP 90-alpha | CHAPERONE | HS90A_HUMAN | Molecular chaperone. Has ATPase activity (By similarity). |
| 2brb_v | 3 | 2.919 | 0.9729 | 0.424462 | 2 | 1 | 0 | 0 | 0 | 0 | Serine/threonine-protein kinase Chk1 | TRANSFERASE | CHK1_HUMAN | Required for checkpoint mediated cell cycle arrest in response to DNA damage or the presence of unreplicated DNA. May also negatively regulate cell cycle progression during unperturbed cell cycles. Recognizes the substrate consensus sequence [R-X-X- S/T]. |
| 1md3_v | 3 | 2.916 | 0.9722 | 0.289531 | 1 | 2 | 0 | 0 | 0 | 0 | Glutathione S-transferase P | TRANSFERASE | GSTP1_HUMAN | Conjugation of reduced glutathione to a wide number of exogenous and endogenous hydrophobic electrophiles. |
| 3hvc_v | 3 | 2.904 | 0.9679 | 0.339402 | 3 | 0 | 0 | 0 | 0 | 0 | NONE | NONE | NONE | NONE |
| 3dbu_v | 3 | 2.896 | 0.9652 | 0.550526 | 1 | 2 | 0 | 0 | 0 | 0 | Carbonic anhydrase 2 | NONE | P00918 | Inorganic ion transport and metabolism |
| 1rs0_v | 3 | 2.895 | 0.965 | 0.094721 | 2 | 1 | 0 | 0 | 0 | 0 | Complement factor B | HYDROLASE | CFAB_HUMAN | Factor B which is part of the alternate pathway of the complement system is cleaved by factor D into 2 fragments: Ba and Bb. Bb, a serine protease, then combines with complement factor 3b to generate the C3 or C5 convertase. It has also been implicated in |
| 1bzm_v | 3 | 2.893 | 0.9644 | 0.49006 | 0 | 1 | 2 | 0 | 0 | 0 | Carbonic anhydrase 1 | LYASE(OXO-ACID) | CAH1_HUMAN | Reversible hydration of carbon dioxide. |
| 3cgf_v | 3 | 2.892 | 0.9641 | 0.269923 | 2 | 0 | 1 | 0 | 0 | 0 | Mitogen-activated protein kinase 10 | TRANSFERASE | MK10_HUMAN | Responds to activation by environmental stress and pro- inflammatory cytokines by phosphorylating a number of transcription factors, primarily components of AP-1 such as c-Jun and ATF2 and thus regulates AP-1 transcriptional activity. Required for stress- |
| 3cjf_v | 3 | 2.892 | 0.9641 | 0.28794 | 2 | 0 | 1 | 0 | 0 | 0 | Vascular endothelial growth factor receptor 2 | NONE | P35968 | Tyrosine-protein kinase that acts as a cell-surface receptor for VEGFA, VEGFC and VEGFD. Plays an essential role in the regulation of angiogenesis, vascular development, vascular permeability, and embryonic hematopoiesis. Promotes proliferation, survival, migration and differentiation of endothelial cells. Promotes reorganization of the actin cytoskeleton. Isoforms lacking a transmembrane domain, such as isoform 2 and isoform 3, may function as decoy receptors for VEGFA, VEGFC and/or VEGFD. Isoform 2 plays an important role as negative regulator of VEGFA- and VEGFC-mediated lymphangiogenesis by limiting the amount of free VEGFA and/or VEGFC and preventing their binding to FLT4. Modulates FLT1 and FLT4 signaling by forming heterodimers. Binding of vascular growth factors to isoform 1 leads to the activation of several signaling cascades. Activation of PLCG1 leads to the production of the cellular signaling molecules diacylglycerol and inositol 1,4,5-trisphosphate and the activation of protein kinase C. Mediates activation of MAPK1/ERK2, MAPK3/ERK1 and the MAP kinase signaling pathway, as well as of the AKT1 signaling pathway. Mediates phosphorylation of PIK3R1, the regulatory subunit of phosphatidylinositol 3-kinase, reorganization of the actin cytoskeleton and activation of PTK2/FAK1. Required for VEGFA-mediated induction of NOS2 and NOS3, leading to the production of the signaling molecule nitric oxide (NO) by endothelial cells. Phosphorylates PLCG1. Promotes phosphorylation of FYN, NCK1, NOS3, PIK3R1, PTK2/FAK1 and SRC. |
| 2agt_v | 3 | 2.89 | 0.9634 | 0.457111 | 2 | 1 | 0 | 0 | 0 | 0 | Aldose reductase | OXIDOREDUCTASE | ALDR_HUMAN | Catalyzes the NADPH-dependent reduction of a wide variety of carbonyl-containing compounds to their corresponding alcohols with a broad range of catalytic efficiencies. |
| 2p3g_v | 3 | 2.889 | 0.9629 | 0.056297 | 3 | 0 | 0 | 0 | 0 | 0 | MAP kinase-activated protein kinase 2 | NONE | P49137 | Involved in protein kinase activity |
| 2vwu_v | 3 | 2.883 | 0.961 | 0.371915 | 2 | 1 | 0 | 0 | 0 | 0 | Ephrin type-B receptor 4 | TRANSFERASE | EPHB4_HUMAN | Receptor for members of the ephrin-B family. Binds to ephrin-B2. May have a role in events mediating differentiation and development. |
| 2pio_v | 3 | 2.882 | 0.9607 | 0.34602 | 2 | 0 | 1 | 0 | 0 | 0 | Androgen receptor | HORMONE RECEPTOR | ANDR_HUMAN | Steroid hormone receptors are ligand-activated transcription factors that regulate eukaryotic gene expression and affect cellular proliferation and differentiation in target tissues. Transcription factor activity is modulated by bound coactivator and core |
| 1y7v_v | 3 | 2.878 | 0.9592 | 0.53263 | 0 | 1 | 2 | 0 | 0 | 0 | Glucosylceramidase | HYDROLASE | GLCM_HUMAN | NONE |
| 1p0i_v | 3 | 2.875 | 0.9582 | 0.1449 | 1 | 2 | 0 | 0 | 0 | 0 | Cholinesterase | HYDROLASE | CHLE_HUMAN | NONE |
| 2o65_v | 3 | 2.874 | 0.958 | 0.064673 | 3 | 0 | 0 | 0 | 0 | 0 | Proto-oncogene serine/threonine-protein kinase Pim-1 | TRANSFERASE | PIM1_HUMAN | Plays a role in signal transduction in blood cells. Contributes to both cell proliferation and survival and thus provide a selective advantage in tumorigenesis. May affect the structure or silencing of chromatin by phosphorylating HP1 gamma/CBX3. |
| 1e1x_v | 3 | 2.866 | 0.9553 | 0.349316 | 1 | 1 | 1 | 0 | 0 | 0 | Cell division protein kinase 2 | NONE | P24941 | Involved in protein kinase activity |
| 1h1p_v | 3 | 2.865 | 0.955 | 0.355387 | 1 | 1 | 1 | 0 | 0 | 0 | Cyclin-A2 | TRANSFERASE | CCNA2_HUMAN | Essential for the control of the cell cycle at the G1/S (start) and the G2/M (mitosis) transitions. |
| 1ya8_v | 3 | 2.859 | 0.9532 | 0.255876 | 2 | 1 | 0 | 0 | 0 | 0 | Liver carboxylesterase 1 | HYDROLASE | EST1_HUMAN | Involved in the detoxification of xenobiotics and in the activation of ester and amide prodrugs. Hydrolyzes aromatic and aliphatic esters, but has no catalytic activity toward amides or a fatty acyl CoA ester. |
| 2i0j_v | 3 | 2.858 | 0.9527 | 0.360295 | 2 | 1 | 0 | 0 | 0 | 0 | Estrogen receptor | TRANSCRIPTION | ESR1_HUMAN | Nuclear hormone receptor. The steroid hormones and their receptors are involved in the regulation of eukaryotic gene expression and affect cellular proliferation and differentiation in target tissues. |
| 1e7a_v | 3 | 2.84 | 0.9468 | 0.19686 | 3 | 0 | 0 | 0 | 0 | 0 | Serum albumin | CARRIER PROTEIN | ALBU_HUMAN | Serum albumin, the main protein of plasma, has a good binding capacity for water, Ca(2+), Na(+), K(+), fatty acids, hormones, bilirubin and drugs. Its main function is the regulation of the colloidal osmotic pressure of blood. |
| 2ovm_v | 3 | 2.84 | 0.9467 | 0.316821 | 2 | 1 | 0 | 0 | 0 | 0 | Progesterone receptor | TRANSCRIPTION | PRGR_HUMAN | The steroid hormones and their receptors are involved in the regulation of eukaryotic gene expression and affect cellular proliferation and differentiation in target tissues. |
| 1dvz_v | 3 | 2.79 | 0.9301 | -0.22527 | 2 | 1 | 0 | 0 | 0 | 0 | Transthyretin | HORMONE/GROWTH FACTOR | TTHY_HUMAN | Thyroid hormone-binding protein. Probably transports thyroxine from the bloodstream to the brain. |
| 1no9_v | 3 | 2.782 | 0.9273 | -0.16233 | 2 | 1 | 0 | 0 | 0 | 0 | Prothrombin | HYDROLASE | THRB_HUMAN | Thrombin, which cleaves bonds after Arg and Lys, converts fibrinogen to fibrin and activates factors V, VII, VIII, XIII, and, in complex with thrombomodulin, protein C. Functions in blood homeostasis, inflammation and wound healing. |
| 1v3q_v | 3 | 2.782 | 0.9272 | 0.056308 | 1 | 2 | 0 | 0 | 0 | 0 | Purine nucleoside phosphorylase | TRANSFERASE | PNPH_HUMAN | NONE |
| 1fh0_v | 3 | 2.779 | 0.9264 | 0.191868 | 1 | 1 | 1 | 0 | 0 | 0 | Cathepsin L2 | HYDROLASE | CATL2_HUMAN | Cysteine protease. May have an important role in corneal physiology. |
| 2zas_v | 4 | 3.705 | 0.9262 | 2.29626 | 3 | 1 | 0 | 0 | 0 | 0 | Estrogen-related receptor gamma | NONE | P62508 | Orphan receptor that acts as transcription activator in the absence of bound ligand. Binds specifically to an estrogen response element and activates reporter genes controlled by estrogen response elements (By similarity). Induces the expression of PERM1 in the skeletal muscle. |
| 2itx_v | 3 | 2.764 | 0.9212 | 0.119449 | 0 | 2 | 1 | 0 | 0 | 0 | Epidermal growth factor receptor | TRANSFERASE | EGFR_HUMAN | Receptor for EGF, but also for other members of the EGF family, as TGF-alpha, amphiregulin, betacellulin, heparin-binding EGF-like growth factor, GP30 and vaccinia virus growth factor. Is involved in the control of cell growth and differentiation. Phospho;Isoform 2/truncated isoform may act as an antagonist. |
| 1xoz_v | 3 | 2.75 | 0.9168 | 0.08639 | 2 | 0 | 1 | 0 | 0 | 0 | cGMP-specific 3,5-cyclic phosphodiesterase | HYDROLASE | PDE5A_HUMAN | Plays a role in signal transduction by regulating the intracellular concentration of cyclic nucleotides. This phosphodiesterase catalyzes the specific hydrolysis of cGMP to 5- GMP. |
| 1unh_v | 3 | 2.747 | 0.9158 | 0.128187 | 2 | 1 | 0 | 0 | 0 | 0 | Cyclin-dependent kinase 5 activator 1 | COMPLEX(KINASE/ACTIVATOR) | CD5R1_HUMAN | p35 is a neuron specific activator of CDK5. The complex p35/CDK5 is required for neurite outgrowth and cortical lamination. Activator of TPKII. |
| 3fzh_v | 3 | 2.731 | 0.9104 | 0.167663 | 0 | 2 | 1 | 0 | 0 | 0 | Heat shock cognate 71 kDa protein | NONE | P11142 | Molecular chaperone implicated in a wide variety of cellular processes, including protection of the proteome from stress, folding and transport of newly synthesized polypeptides, activation of proteolysis of misfolded proteins and the formation and dissociation of protein complexes. Plays a pivotal role in the protein quality control system, ensuring the correct folding of proteins, the re-folding of misfolded proteins and controlling the targeting of proteins for subsequent degradation (PubMed:21150129, PubMed:21148293, PubMed:24732912, PubMed:27916661, PubMed:23018488). This is achieved through cycles of ATP binding, ATP hydrolysis and ADP release, mediated by co-chaperones (PubMed:21150129, PubMed:21148293, PubMed:24732912, PubMed:27916661, PubMed:23018488). The co-chaperones have been shown to not only regulate different steps of the ATPase cycle of HSP70, but they also have an individual specificity such that one co-chaperone may promote folding of a substrate while another may promote degradation (PubMed:21150129, PubMed:21148293, PubMed:24732912, PubMed:27916661, PubMed:23018488). The affinity of HSP70 for polypeptides is regulated by its nucleotide bound state. In the ATP-bound form, it has a low affinity for substrate proteins. However, upon hydrolysis of the ATP to ADP, it undergoes a conformational change that increases its affinity for substrate proteins. HSP70 goes through repeated cycles of ATP hydrolysis and nucleotide exchange, which permits cycles of substrate binding and release. The HSP70-associated co-chaperones are of three types: J-domain co-chaperones HSP40s (stimulate ATPase hydrolysis by HSP70), the nucleotide exchange factors (NEF) such as BAG1/2/3 (facilitate conversion of HSP70 from the ADP-bound to the ATP-bound state thereby promoting substrate release), and the TPR domain chaperones such as HOPX and STUB1 (PubMed:24318877, PubMed:27474739, PubMed:24121476, PubMed:26865365). Acts as a repressor of transcriptional activation. Inhibits the transcriptional coactivator activity of CITED1 on Smad-mediated transcription. Component of the PRP19-CDC5L complex that forms an integral part of the spliceosome and is required for activating pre-mRNA splicing. May have a scaffolding role in the spliceosome assembly as it contacts all other components of the core complex. Binds bacterial lipopolysaccharide (LPS) and mediates LPS-induced inflammatory response, including TNF secretion by monocytes (PubMed:10722728, PubMed:11276205). Participates in the ER-associated degradation (ERAD) quality control pathway in conjunction with J domain-containing co-chaperones and the E3 ligase STUB1 (PubMed:23990462). |
| 3eqm_v | 3 | 2.711 | 0.9037 | -0.11635 | 2 | 1 | 0 | 0 | 0 | 0 | Cytochrome P450 19A1 | NONE | P11511 | Catalyzes the formation of aromatic C18 estrogens from C19 androgens. |
| 1vec_v | 3 | 2.71 | 0.9032 | -0.12885 | 0 | 3 | 0 | 0 | 0 | 0 | Probable ATP-dependent RNA helicase DDX6 | RNA BINDING PROTEIN | DDX6_HUMAN | In the process of mRNA degradation, may play a role in mRNA decapping. |
| 1yb1_v | 3 | 2.709 | 0.9031 | -0.24109 | 2 | 0 | 1 | 0 | 0 | 0 | Estradiol 17-beta-dehydrogenase 11 | OXIDOREDUCTASE | DHB11_HUMAN | Can convert androstan-3-alpha,17-beta-diol (3-alpha- diol) to androsterone in vitro, suggesting that it may participate in androgen metabolism during steroidogenesis. May act by metabolizing compounds that stimulate steroid synthesis and/or by generating |
| 1uki_v | 3 | 2.704 | 0.9014 | -0.46888 | 2 | 1 | 0 | 0 | 0 | 0 | Mitogen-activated protein kinase 8 | TRANSFERASE | MK08_HUMAN | Responds to activation by environmental stress and pro- inflammatory cytokines by phosphorylating a number of transcription factors, primarily components of AP-1 such as JUN, JDP2 and ATF2 and thus regulates AP-1 transcriptional activity. In T-cells, JNK1;JNK1 isoforms display different binding patterns: beta-1 preferentially binds to c-Jun, whereas alpha-1, alpha-2, and beta- 2 have a similar low level of binding to both c-Jun or ATF2. However, there is no correlation between binding and phosphorylation, |
| 1pq9_v | 3 | 2.672 | 0.8907 | -0.25667 | 1 | 2 | 0 | 0 | 0 | 0 | Oxysterols receptor LXR-beta | TRANSCRIPTION REGULATION | NR1H2_HUMAN | Orphan receptor. Binds preferentially to double-stranded oligonucleotide direct repeats having the consensus half-site sequence 5-AGGTCA-3 and 4-nt spacing (DR-4). |
| 1xlz_v | 4 | 3.543 | 0.8857 | 2.018 | 3 | 1 | 0 | 0 | 0 | 0 | cAMP-specific 3,5-cyclic phosphodiesterase 4B | HYDROLASE | PDE4B_HUMAN | May be involved in mediating central nervous system effects of therapeutic agents ranging from antidepressants to antiasthmatic and anti-inflammatory agents. |
| 1klt_v | 3 | 2.656 | 0.8852 | -0.15536 | 1 | 2 | 0 | 0 | 0 | 0 | Chymase | SERINE PROTEASE | CMA1_HUMAN | Major secreted protease of mast cells with suspected roles in vasoactive peptide generation, extracellular matrix degradation, and regulation of gland secretion. |
| 1lyw_v | 3 | 2.637 | 0.8791 | -0.21515 | 1 | 2 | 0 | 0 | 0 | 0 | Cathepsin D | ASPARTIC PROTEASE | CATD_HUMAN | Acid protease active in intracellular protein breakdown. Involved in the pathogenesis of several diseases such as breast cancer and possibly Alzheimer disease. |
| 1m9z_v | 3 | 2.576 | 0.8587 | -0.47427 | 0 | 1 | 2 | 0 | 0 | 0 | TGF-beta receptor type-2 | HORMONE/GROWTH FACTOR | TGFR2_HUMAN | On ligand binding, forms a receptor complex consisting of two type II and two type I transmembrane serine/threonine kinases. Type II receptors phosphorylate and activate type I receptors which autophosphorylate, then bind and activate SMAD transcriptional |
| 1nub_v | 3 | 2.553 | 0.8508 | -0.52535 | 1 | 1 | 1 | 0 | 0 | 0 | SPARC | EXTRACELLULAR MODULE | SPRC_HUMAN | Appears to regulate cell growth through interactions with the extracellular matrix and cytokines. Binds calcium and copper, several types of collagen, albumin, thrombospondin, PDGF and cell membranes. There are two calcium binding sites; an acidic domain |
| 2pin_v | 3 | 2.529 | 0.843 | -1.27771 | 3 | 0 | 0 | 0 | 0 | 0 | Thyroid hormone receptor beta | NONE | P10828 | Nuclear hormone receptor that can act as a repressor or activator of transcription. High affinity receptor for thyroid hormones, including triiodothyronine and thyroxine. |
| 1muo_v | 3 | 2.525 | 0.8416 | -0.52771 | 0 | 2 | 1 | 0 | 0 | 0 | Serine/threonine-protein kinase 6 | TRANSFERASE | STK6_HUMAN | May play a role in cell cycle regulation during anaphase and/or telophase, in relation to the function of the centrosome/spindle pole region during chromosome segregation. May be involved in microtubule formation and/or stabilization. May play a key role |
| 1g1q_v | 3 | 2.522 | 0.8407 | -0.61566 | 1 | 1 | 1 | 0 | 0 | 0 | P-selectin | IMMUNE SYSTEM, MEMBRANE PROTEIN | LYAM3_HUMAN | Ca(2+)-dependent receptor for myeloid cells that binds to carbohydrates on neutrophils and monocytes. Mediates the interaction of activated endothelial cells or platelets with leukocytes. The ligand recognized is sialyl-Lewis X. Mediates rapid rolling of |
| 1zxq_v | 3 | 2.505 | 0.8349 | -0.29566 | 0 | 0 | 3 | 0 | 0 | 0 | Intercellular adhesion molecule 2 | CELL ADHESION | ICAM2_HUMAN | ICAM proteins are ligands for the leukocyte adhesion protein LFA-1 (integrin alpha-L/beta-2). ICAM2 may play a role in lymphocyte recirculation by blocking LFA-1-dependent cell adhesion. It mediates adhesive interactions important for antigen- specific im |
| 1oj9_v | 3 | 2.456 | 0.8185 | -0.80648 | 3 | 0 | 0 | 0 | 0 | 0 | Amine oxidase [flavin-containing] B | OXIDOREDUCTASE | AOFB_HUMAN | Catalyzes the oxidative deamination of biogenic and xenobiotic amines and has important functions in the metabolism of neuroactive and vasoactive amines in the central nervous system and peripheral tissues. MAOB preferentially degrades benzylamine and phe |
| 1zxc_v | 4 | 3.224 | 0.8059 | 0.789997 | 3 | 1 | 0 | 0 | 0 | 0 | ADAM 17 | HYDROLASE | ADA17_HUMAN | Cleaves the membrane-bound precursor of TNF-alpha to its mature soluble form. Responsible for the proteolytic release of several other cell-surface proteins, including p75 TNF-receptor, interleukin 1 receptor type II, p55 TNF-receptor, transforming growth |
| 1ovz_v | 3 | 2.405 | 0.8016 | -0.52434 | 0 | 1 | 2 | 0 | 0 | 0 | Immunoglobulin alpha Fc receptor | IMMUNE SYSTEM | FCAR_HUMAN | Binds to the Fc region of immunoglobulins alpha. Mediates several functions including cytokine production. |
| 1bm6_v | 3 | 2.356 | 0.7853 | -1.47141 | 2 | 1 | 0 | 0 | 0 | 0 | Stromelysin-1 | METALLOPROTEASE | MMP3_HUMAN | Can degrade fibronectin, laminin, gelatins of type I, III, IV, and V; collagens III, IV, X, and IX, and cartilage proteoglycans. Activates procollagenase. |
| 2g8n_v | 5 | 3.909 | 0.7818 | 2.87139 | 3 | 0 | 1 | 1 | 0 | 0 | Phenylethanolamine N-methyltransferase | TRANSFERASE | PNMT_HUMAN | Converts noradrenaline to adrenaline. |
| 1k27_v | 5 | 3.896 | 0.7791 | 2.09923 | 1 | 1 | 3 | 0 | 0 | 0 | S-methyl-5-thioadenosine phosphorylase | NONE | Q13126 | Nucleotide transport and metabolism |
| 2f1g_v | 3 | 2.336 | 0.7786 | -0.81141 | 1 | 1 | 1 | 0 | 0 | 0 | Cathepsin S | HYDROLASE | CATS_HUMAN | Thiol protease. Key protease responsible for the removal of the invariant chain from MHC class II molecules. The bond- specificity of this proteinase is in part similar to the specificities of cathepsin L and cathepsin N. |
| 1jd0_v | 3 | 2.328 | 0.7761 | -0.93477 | 0 | 2 | 1 | 0 | 0 | 0 | Carbonic anhydrase 12 | LYASE | CAH12_HUMAN | Reversible hydration of carbon dioxide. |
| 2pe2_v | 5 | 3.798 | 0.7597 | 1.53751 | 1 | 3 | 1 | 0 | 0 | 0 | 3-phosphoinositide-dependent protein kinase 1 | TRANSFERASE | PDPK1_HUMAN | Phosphorylates and activates not only PKB/AKT, but also PKA, PKC-zeta, RPS6KA1 and RPS6KB1. May play a general role in signaling processes and in development (By similarity). Isoform 3 is catalytically inactive. |
| 3ey4_v | 5 | 3.797 | 0.7593 | 2.61358 | 3 | 1 | 1 | 0 | 0 | 0 | Corticosteroid 11-beta-dehydrogenase isozyme 1 | NONE | P28845 | Lipid transport and metabolism |
| 1q41_v | 5 | 3.74 | 0.7481 | 2.67116 | 2 | 2 | 1 | 0 | 0 | 0 | Glycogen synthase kinase-3 beta | TRANSFERASE | GSK3B_HUMAN | Participates in the Wnt signaling pathway. Implicated in the hormonal control of several regulatory proteins including glycogen synthase, MYB and the transcription factor JUN. Phosphorylates JUN at sites proximal to its DNA-binding domain, thereby reducin |
| 1n83_v | 4 | 2.985 | 0.7463 | 0.093923 | 4 | 0 | 0 | 0 | 0 | 0 | Nuclear receptor ROR-alpha | LIPID BINDING PROTEIN | RORA_HUMAN | Orphan nuclear receptor. Binds DNA as a monomer to hormone response elements (HRE) containing a single core motif half-site preceded by a short A-T-rich sequence. This isomer binds to the consensus sequence 5-[AT][TA]A[AT][CGT]TAGGTCA-3. |
| 2h8h_v | 4 | 2.984 | 0.7459 | 0.285394 | 3 | 1 | 0 | 0 | 0 | 0 | Proto-oncogene tyrosine-protein kinase Src | TRANSFERASE | SRC_HUMAN | NONE |
| 1l6m_v | 4 | 2.983 | 0.7458 | 0.06365 | 1 | 1 | 1 | 0 | 1 | 0 | Neutrophil gelatinase-associated lipocalin | TRANSPORT PROTEIN | NGAL_HUMAN | Transport of small lipophilic substances (Potential). |
| 3c3u_v | 4 | 2.983 | 0.7457 | 0.551592 | 2 | 1 | 0 | 0 | 1 | 0 | Aldo-keto reductase family 1 member C1 | NONE | Q04828 | Involved in oxidoreductase activity |
| 3pah_v | 4 | 2.977 | 0.7444 | 0.447019 | 1 | 1 | 1 | 1 | 0 | 0 | Phenylalanine-4-hydroxylase | MONOOXGYGENASE | PH4H_HUMAN | NONE |
| 1n1m_v | 4 | 2.975 | 0.7437 | 0.38222 | 2 | 1 | 0 | 1 | 0 | 0 | Dipeptidyl peptidase 4 | HYDROLASE | DPP4_HUMAN | Removes N-terminal dipeptides sequentially from polypeptides having unsubstituted N-termini provided that the penultimate residue is proline. Plays a role in T-cell activation. |
| 1j78_v | 4 | 2.974 | 0.7435 | -0.60019 | 4 | 0 | 0 | 0 | 0 | 0 | Vitamin D-binding protein | TRANSPORT, LIGAND BINDING PROTEIN | VTDB_HUMAN | Multifunctional protein found in plasma, ascitic fluid, cerebrospinal fluid, and urine and on the surface of many cell types. In plasma, it carries the vitamin D sterols and prevents polymerization of actin by binding its monomers. DBP associates with mem |
| 1b6a_v | 4 | 2.97 | 0.7426 | 0.535325 | 3 | 0 | 1 | 0 | 0 | 0 | Methionine aminopeptidase 2 | ANGIOGENESIS INHIBITOR | AMPM2_HUMAN | Removes the amino-terminal methionine from nascent proteins. |
| 1j99_v | 4 | 2.966 | 0.7415 | 0.220705 | 3 | 0 | 1 | 0 | 0 | 0 | Bile salt sulfotransferase | NONE | Q06520 | Involved in sulfotransferase activity |
| 1kta_v | 4 | 2.966 | 0.7415 | -0.09287 | 1 | 2 | 0 | 0 | 1 | 0 | Branched-chain-amino-acid aminotransferase, mitochondrial | NONE | O15382 | Amino acid transport and metabolism |
| 2f6y_v | 4 | 2.96 | 0.7399 | 0.393581 | 2 | 1 | 0 | 0 | 1 | 0 | Tyrosine-protein phosphatase non-receptor type 1 | HYDROLASE | PTN1_HUMAN | May play an important role in CKII- and p60c-src-induced signal transduction cascades (By similarity). |
| 1mfu_v | 4 | 2.959 | 0.7398 | 0.347745 | 0 | 2 | 2 | 0 | 0 | 0 | Alpha-amylase 1 | NONE | P04745 | Carbohydrate transport and metabolism |
| 1hak_v | 4 | 2.958 | 0.7396 | -0.28434 | 3 | 1 | 0 | 0 | 0 | 0 | Annexin A5 | CALCIUM/PHOSPHOLIPID-BINDING | ANXA5_HUMAN | This protein is an anticoagulant protein that acts as an indirect inhibitor of the thromboplastin-specific complex, which is involved in the blood coagulation cascade. |
| 1kbo_v | 4 | 2.957 | 0.7391 | 0.316427 | 3 | 0 | 1 | 0 | 0 | 0 | NAD(P)H dehydrogenase [quinone] 1 | OXIDOREDUCTASE | NQO1_HUMAN | The enzyme apparently serves as a quinone reductase in connection with conjugation reactions of hydroquinons involved in detoxification pathways as well as in biosynthetic processes such as the vitamin K-dependent gamma-carboxylation of glutamate residues |
| 1mkd_v | 4 | 2.943 | 0.7357 | 0.260075 | 2 | 2 | 0 | 0 | 0 | 0 | cAMP-specific 3,5-cyclic phosphodiesterase 4D | HYDROLASE | PDE4D_HUMAN | Regulates the levels of cAMP in the cell. |
| 1xf0_v | 3 | 2.202 | 0.7341 | -1.15717 | 2 | 1 | 0 | 0 | 0 | 0 | Aldo-keto reductase family 1 member C3 | OXIDOREDUCTASE | AK1C3_HUMAN | Catalyzes the conversion of aldehydes and ketones to alcohols. Catalyzes the reduction of prostaglandin (PG) D2, PGH2 and phenanthrenequinone (PQ) and the oxidation of 9-alpha,11-beta- PGF2 to PGD2. Functions as a bi-directional 3-alpha-, 17-beta- and 20- |
| 1vjy_v | 4 | 2.93 | 0.7326 | -0.09864 | 3 | 1 | 0 | 0 | 0 | 0 | TGF-beta receptor type-1 | TRANSFERASE | TGFR1_HUMAN | On ligand binding, forms a receptor complex consisting of two type II and two type I transmembrane serine/threonine kinases. Type II receptors phosphorylate and activate type I receptors which autophosphorylate, then bind and activate SMAD transcriptional |
| 1ya3_v | 4 | 2.924 | 0.7309 | 0.209086 | 3 | 1 | 0 | 0 | 0 | 0 | Mineralocorticoid receptor | TRANSCRIPTION | MCR_HUMAN | Receptor for both mineralocorticoids (MC) such as aldosterone and glucocorticoids (GC) such as corticosterone or cortisol. Binds to mineralocorticoid response elements (MRE) and transactivates target genes. The effect of MC is to increase ion and water tr |
| 1q8m_v | 3 | 2.192 | 0.7307 | -1.13605 | 1 | 2 | 0 | 0 | 0 | 0 | Triggering receptor expressed on myeloid cells 1 | NONE | Q9NP99 | Involved in immune triggering activity |
| 1ca7_v | 4 | 2.922 | 0.7306 | 0.226874 | 1 | 2 | 0 | 0 | 1 | 0 | Macrophage migration inhibitory factor | CYTOKINE | MIF_HUMAN | The expression of MIF at sites of inflammation suggest a role for the mediator in regulating the function of macrophage in host defense. Also acts as a phenylpyruvate tautomerase. |
| 1a8j_v | 4 | 2.917 | 0.7291 | 0.453892 | 1 | 2 | 0 | 1 | 0 | 0 | Ig lambda chain V-II region MGC | NONE | P01709 | V region of the variable domain of immunoglobulin light chains that participates in the antigen recognition (PubMed:24600447). Immunoglobulins, also known as antibodies, are membrane-bound or secreted glycoproteins produced by B lymphocytes. In the recognition phase of humoral immunity, the membrane-bound immunoglobulins serve as receptors which, upon binding of a specific antigen, trigger the clonal expansion and differentiation of B lymphocytes into immunoglobulins-secreting plasma cells. Secreted immunoglobulins mediate the effector phase of humoral immunity, which results in the elimination of bound antigens (PubMed:20176268, PubMed:22158414). The antigen binding site is formed by the variable domain of one heavy chain, together with that of its associated light chain. Thus, each immunoglobulin has two antigen binding sites with remarkable affinity for a particular antigen. The variable domains are assembled by a process called V-(D)-J rearrangement and can then be subjected to somatic hypermutations which, after exposure to antigen and selection, allow affinity maturation for a particular antigen (PubMed:17576170, PubMed:20176268). |
| 1ld7_v | 4 | 2.916 | 0.7291 | 0.306688 | 2 | 1 | 0 | 1 | 0 | 0 | Protein farnesyltransferase/geranylgeranyltransferase type-1 subunit alpha | TRANSFERASE | FNTA_HUMAN | Catalyzes the transfer of a farnesyl or geranyl-geranyl moiety from farnesyl or geranyl-geranyl pyrophosphate to a cysteine at the fourth position from the C-terminus of several proteins having the C-terminal sequence Cys-aliphatic-aliphatic-X. The alpha |
| 1fm6_v | 4 | 2.916 | 0.729 | -0.07521 | 3 | 1 | 0 | 0 | 0 | 0 | Peroxisome proliferator-activated receptor gamma | TRANSCRIPTION | PPARG_HUMAN | Receptor that binds peroxisome proliferators such as hypolipidemic drugs and fatty acids. Once activated by a ligand, the receptor binds to a promoter element in the gene for acyl-CoA oxidase and activates its transcription. It therefore controls the pero |
| 3f5p_v | 4 | 2.916 | 0.7289 | 0.229337 | 3 | 1 | 0 | 0 | 0 | 0 | Insulin-like growth factor 1 receptor | NONE | P08069 | Involved in transmembrane receptor protein tyrosine kinase activity |
| 2j7x_v | 4 | 2.915 | 0.7288 | 0.293335 | 2 | 1 | 1 | 0 | 0 | 0 | Estrogen receptor beta | NONE | Q62986 | Involved in transcription factor activity |
| 1xur_v | 4 | 2.912 | 0.728 | 0.394737 | 2 | 2 | 0 | 0 | 0 | 0 | Collagenase 3 | HYDROLASE | MMP13_HUMAN | Degrades collagen type I. Does not act on gelatin or casein. Could have a role in tumoral process. |
| 1d3g_v | 4 | 2.9 | 0.7251 | 0.009705 | 3 | 0 | 1 | 0 | 0 | 0 | Dihydroorotate dehydrogenase, mitochondrial | OXIDOREDUCTASE | PYRD_HUMAN | NONE |
| 1fdu_v | 4 | 2.896 | 0.7239 | 0.027605 | 3 | 0 | 1 | 0 | 0 | 0 | Estradiol 17-beta-dehydrogenase 1 | NONE | P14061 | Lipid transport and metabolism |
| 1dic_v | 4 | 2.89 | 0.7224 | 0.156527 | 2 | 2 | 0 | 0 | 0 | 0 | Complement factor D | SERINE PROTEASE | CFAD_HUMAN | Factor D cleaves factor B when the latter is complexed with factor C3b, activating the C3bbb complex, which then becomes the C3 convertase of the alternate pathway. Its function is homologous to that of C1s in the classical pathway. |
| 1xo2_v | 5 | 3.605 | 0.7209 | 1.97573 | 2 | 3 | 0 | 0 | 0 | 0 | Cell division protein kinase 6 | CELL CYCLE/TRANSFERASE | CDK6_HUMAN | Probably involved in the control of the cell cycle. Interacts with D-type G1 cyclins. |
| 1xan_v | 4 | 2.881 | 0.7203 | 0.014824 | 2 | 1 | 1 | 0 | 0 | 0 | Glutathione reductase, mitochondrial | NONE | P00390 | RNA processing and modification |
| 1juj_v | 4 | 2.875 | 0.7188 | -0.36883 | 2 | 1 | 0 | 0 | 1 | 0 | Thymidylate synthase | TRANSFERASE | TYSY_HUMAN | NONE |
| 3dy6_v | 4 | 2.868 | 0.7169 | -0.098 | 3 | 1 | 0 | 0 | 0 | 0 | Peroxisome proliferator-activated receptor delta | NONE | Q03181 | Involved in DNA binding |
| 2of4_v | 4 | 2.861 | 0.7151 | 0.254479 | 2 | 1 | 0 | 1 | 0 | 0 | Proto-oncogene tyrosine-protein kinase LCK | TRANSFERASE | LCK_HUMAN | Tyrosine kinase that plays an essential role for the selection and maturation of developing T-cell in the thymus and in mature T-cell function. Is constitutively associated with the cytoplasmic portions of the CD4 and CD8 surface receptors and plays a key |
| 1uou_v | 4 | 2.86 | 0.7151 | 0.416245 | 1 | 0 | 3 | 0 | 0 | 0 | Thymidine phosphorylase | TRANSFERASE | TYPH_HUMAN | May have a role in maintaining the integrity of the blood vessels. Has growth promoting activity on endothelial cells, angiogenic activity in vivo and chemotactic activity on endothelial cells in vitro.;Catalyzes the reversible phosphorolysis of thymidine. The produced molecules are then utilized as carbon and energy sources or in the rescue of pyrimidine bases for nucleotide synthesis. |
| 1agw_v | 4 | 2.859 | 0.7148 | 0.072851 | 2 | 1 | 1 | 0 | 0 | 0 | Basic fibroblast growth factor receptor 1 | PROTEIN KINASE | FGFR1_HUMAN | Receptor for basic fibroblast growth factor. A shorter form of the receptor could be a receptor for FGF1 (aFGF). |
| 3cqu_v | 4 | 2.854 | 0.7135 | 0.220657 | 2 | 1 | 1 | 0 | 0 | 0 | RAC-alpha serine/threonine-protein kinase | TRANSFERASE | AKT1_HUMAN | General protein kinase capable of phosphorylating several known proteins. Phosphorylates TBC1D4. Signals downstream of phosphatidylinositol 3-kinase (PI(3)K) to mediate the effects of various growth factors such as platelet-derived growth factor (PDGF), e |
| 1wlj_v | 5 | 3.556 | 0.7111 | 1.58431 | 0 | 4 | 1 | 0 | 0 | 0 | Interferon-stimulated gene 20 kDa protein | HYDROLASE | ISG20_HUMAN | Exonuclease with specificity for single-stranded RNA and, to a lesser extent for DNA. Degrades RNA at a rate that is approximately 35-fold higher than its rate for single-stranded DNA. Involved in the antiviral function of IFN against RNA viruses. |
| 1nfx_v | 4 | 2.843 | 0.7107 | 0.110952 | 2 | 1 | 0 | 0 | 0 | 1 | Coagulation factor X | HYDROLASE | FA10_HUMAN | Factor Xa is a vitamin K-dependent glycoprotein that converts prothrombin to thrombin in the presence of factor Va, calcium and phospholipid during blood clotting. |
| 2g1y_v | 4 | 2.836 | 0.709 | 0.243004 | 2 | 1 | 1 | 0 | 0 | 0 | Renin | HYDROLASE | RENI_HUMAN | Renin is a highly specific endopeptidase, whose only known function is to generate angiotensin I from angiotensinogen in the plasma, initiating a cascade of reactions that produce an elevation of blood pressure and increased sodium retention by the kidney |
| 1k59_v | 4 | 2.836 | 0.7089 | -0.43131 | 0 | 4 | 0 | 0 | 0 | 0 | Angiogenin | HYDROLASE | ANGI_HUMAN | May function as a tRNA-specific ribonuclease that binds to actin on the surface of endothelial cells; once bound, angiogenin is endocytosed and translocated to the nucleus, thereby promoting the endothelial invasiveness necessary for blood vessel formatio |
| 1gpz_v | 4 | 2.829 | 0.7073 | 0.332443 | 1 | 1 | 2 | 0 | 0 | 0 | Complement C1r subcomponent | NONE | P00736 | C1r B chain is a serine protease that combines with C1q and C1s to form C1, the first component of the classical pathway of the complement system |
| 1u71_v | 4 | 2.827 | 0.7068 | 0.070196 | 2 | 0 | 2 | 0 | 0 | 0 | Dihydrofolate reductase | OXIDOREDUCTASE | DYR_HUMAN | NONE |
| 1tow_v | 4 | 2.822 | 0.7055 | -0.68923 | 2 | 1 | 0 | 0 | 1 | 0 | Fatty acid-binding protein, adipocyte | LIPID TRANSPORT | FABP4_HUMAN | Lipid transport protein in adipocytes. Binds both long chain fatty acids and retinoic acid. Delivers long-chain fatty acids and retinoic acid to their cognate receptors in the nucleus (By similarity). |
| 1fa9_v | 4 | 2.821 | 0.7053 | 0.048282 | 0 | 2 | 2 | 0 | 0 | 0 | Glycogen phosphorylase, liver form | NONE | P06737 | Carbohydrate transport and metabolism |
| 1imb_v | 5 | 3.518 | 0.7035 | 0.491118 | 0 | 3 | 2 | 0 | 0 | 0 | Inositol monophosphatase | HYDROLASE | IMPA1_HUMAN | Responsible for the provision of inositol required for synthesis of phosphatidylinositol and polyphosphoinositides and has been implicated as the pharmacological target for lithium action in brain. |
| 1w6j_v | 5 | 3.517 | 0.7033 | 1.66631 | 2 | 2 | 1 | 0 | 0 | 0 | Lanosterol synthase | ISOMERASE | ERG7_HUMAN | Catalyzes the cyclization of (S)-2,3 oxidosqualene to lanosterol, a reaction that forms the sterol nucleus. |
| 1ctr_v | 5 | 3.478 | 0.6955 | 1.13551 | 5 | 0 | 0 | 0 | 0 | 0 | Calmodulin | CALCIUM-BINDING PROTEIN | CALM_HUMAN | Calmodulin mediates the control of a large number of enzymes and other proteins by Ca(2+). Among the enzymes to be stimulated by the calmodulin-Ca(2+) complex are a number of protein kinases and phosphatases. Together with CEP110 and centrin, is involved |
| 1i72_v | 4 | 2.775 | 0.6938 | 0.237864 | 0 | 3 | 1 | 0 | 0 | 0 | S-adenosylmethionine decarboxylase proenzyme | LYASE | DCAM_HUMAN | NONE |
| 2acl_v | 4 | 2.755 | 0.6887 | -0.27393 | 3 | 1 | 0 | 0 | 0 | 0 | Retinoic acid receptor RXR-alpha | TRANSCRIPTION | RXRA_HUMAN | Nuclear hormone receptor. Involved in the retinoic acid response pathway. Binds 9-cis retinoic acid (9C-RA). ARF6 acts as a key regulator of the tissue-specific adipocyte P2 (aP2) enhancer (By similarity). |
| 1m4u_v | 4 | 2.749 | 0.6872 | -0.14523 | 0 | 3 | 1 | 0 | 0 | 0 | Bone morphogenetic protein 7 | HORMONE/GROWTH FACTOR | BMP7_HUMAN | Induces cartilage and bone formation. May be the osteoinductive factor responsible for the phenomenon of epithelial osteogenesis. Plays a role in calcium regulation and bone homeostasis. |
| 1soj_v | 4 | 2.744 | 0.686 | 0.074135 | 1 | 2 | 0 | 0 | 0 | 1 | cGMP-inhibited 3,5-cyclic phosphodiesterase B | HYDROLASE | PDE3B_HUMAN | May play a role in fat metabolism. |
| 1m9r_v | 4 | 2.739 | 0.6848 | -0.37701 | 2 | 1 | 0 | 1 | 0 | 0 | Nitric oxide synthase, endothelial | OXIDOREDUCTASE | NOS3_HUMAN | Produces nitric oxide (NO) which is implicated in vascular smooth muscle relaxation through a cGMP-mediated signal transduction pathway. NO mediates vascular endothelial growth factor (VEGF)-induced angiogenesis in coronary vessels and promotes blood clot |
| 2rku_v | 4 | 2.727 | 0.6817 | -0.15502 | 2 | 1 | 1 | 0 | 0 | 0 | Serine/threonine-protein kinase PLK1 | NONE | P53350 | Serine/threonine-protein kinase that performs several important functions throughout M phase of the cell cycle, including the regulation of centrosome maturation and spindle assembly, the removal of cohesins from chromosome arms, the inactivation of anaphase-promoting complex/cyclosome (APC/C) inhibitors, and the regulation of mitotic exit and cytokinesis. Polo-like kinase proteins acts by binding and phosphorylating proteins are that already phosphorylated on a specific motif recognized by the POLO box domains. Phosphorylates BORA, BUB1B/BUBR1, CCNB1, CDC25C, CEP55, ECT2, ERCC6L, FBXO5/EMI1, FOXM1, KIF20A/MKLP2, CENPU, NEDD1, NINL, NPM1, NUDC, PKMYT1/MYT1, KIZ, PPP1R12A/MYPT1, PRC1, RACGAP1/CYK4, SGO1, STAG2/SA2, TEX14, TOPORS, p73/TP73, TPT1 and WEE1. Plays a key role in centrosome functions and the assembly of bipolar spindles by phosphorylating KIZ, NEDD1 and NINL. NEDD1 phosphorylation promotes subsequent targeting of the gamma-tubulin ring complex (gTuRC) to the centrosome, an important step for spindle formation. Phosphorylation of NINL component of the centrosome leads to NINL dissociation from other centrosomal proteins. Involved in mitosis exit and cytokinesis by phosphorylating CEP55, ECT2, KIF20A/MKLP2, CENPU, PRC1 and RACGAP1. Recruited at the central spindle by phosphorylating and docking PRC1 and KIF20A/MKLP2; creates its own docking sites on PRC1 and KIF20A/MKLP2 by mediating phosphorylation of sites subsequently recognized by the POLO box domains. Phosphorylates RACGAP1, thereby creating a docking site for the Rho GTP exchange factor ECT2 that is essential for the cleavage furrow formation. Promotes the central spindle recruitment of ECT2. Plays a central role in G2/M transition of mitotic cell cycle by phosphorylating CCNB1, CDC25C, FOXM1, CENPU, PKMYT1/MYT1, PPP1R12A/MYPT1 and WEE1. Part of a regulatory circuit that promotes the activation of CDK1 by phosphorylating the positive regulator CDC25C and inhibiting the negative regulators WEE1 and PKMYT1/MYT1. Also acts by mediating phosphorylation of cyclin-B1 (CCNB1) on centrosomes in prophase. Phosphorylates FOXM1, a key mitotic transcription regulator, leading to enhance FOXM1 transcriptional activity. Involved in kinetochore functions and sister chromatid cohesion by phosphorylating BUB1B/BUBR1, FBXO5/EMI1 and STAG2/SA2. PLK1 is high on non-attached kinetochores suggesting a role of PLK1 in kinetochore attachment or in spindle assembly checkpoint (SAC) regulation. Required for kinetochore localization of BUB1B. Regulates the dissociation of cohesin from chromosomes by phosphorylating cohesin subunits such as STAG2/SA2. Phosphorylates SGO1: required for spindle pole localization of isoform 3 of SGO1 and plays a role in regulating its centriole cohesion function. Mediates phosphorylation of FBXO5/EMI1, a negative regulator of the APC/C complex during prophase, leading to FBXO5/EMI1 ubiquitination and degradation by the proteasome. Acts as a negative regulator of p53 family members: phosphorylates TOPORS, leading to inhibit the sumoylation of p53/TP53 and simultaneously enhance the ubiquitination and subsequent degradation of p53/TP53. Phosphorylates the transactivation domain of the transcription factor p73/TP73, leading to inhibit p73/TP73-mediated transcriptional activation and pro-apoptotic functions. Phosphorylates BORA, and thereby promotes the degradation of BORA. Contributes to the regulation of AURKA function. Also required for recovery after DNA damage checkpoint and entry into mitosis. Phosphorylates MISP, leading to stabilization of cortical and astral microtubule attachments required for proper spindle positioning (PubMed:8991084, PubMed:11202906, PubMed:12207013, PubMed:12447691, PubMed:12524548, PubMed:12738781, PubMed:12852856, PubMed:12939256, PubMed:14532005, PubMed:14734534, PubMed:15070733, PubMed:15148369, PubMed:15469984, PubMed:16198290, PubMed:16247472, PubMed:16980960, PubMed:17081991, PubMed:17351640, PubMed:17376779, PubMed:17617734, PubMed:18174154, PubMed:18331714, PubMed:18418051, PubMed:18477460, PubMed:18521620, PubMed:18615013, PubMed:19160488, PubMed:19351716, PubMed:19468300, PubMed:19468302, PubMed:19473992, PubMed:19509060, PubMed:19597481, PubMed:23455478, PubMed:23509069). Together with MEIKIN, acts as a regulator of kinetochore function during meiosis I: required both for mono-orientation of kinetochores on sister chromosomes and protection of centromeric cohesin from separase-mediated cleavage (By similarity). Phosphorylates CEP68 and is required for its degradation (PubMed:25503564). Regulates nuclear envelope breakdown during prophase by phosphorylating DCTN1 resulting in its localization in the nuclear envelope (PubMed:20679239). Phosphorylates the heat shock transcription factor HSF1, promoting HSF1 nuclear translocation upon heat shock (PubMed:15661742). Phosphorylates HSF1 also in the early mitotic period; this phosphorylation regulates HSF1 localization to the spindle pole, the recruitment of the SCF(BTRC) ubiquitin ligase complex induicing HSF1 degradation, and hence mitotic progression (PubMed:18794143). |
| 1lhw_v | 4 | 2.724 | 0.6811 | -0.54027 | 2 | 2 | 0 | 0 | 0 | 0 | Sex hormone-binding globulin | TRANSPORT PROTEIN | SHBG_HUMAN | Functions as an androgen transport protein, but may also be involved in receptor mediated processes. Each dimer binds one molecule of steroid. Specific for 5-alpha-dihydrotestosterone, testosterone, and 17-beta-estradiol. Regulates the plasma metabolic cl |
| 1fv9_v | 3 | 2.017 | 0.6725 | -0.93811 | 0 | 0 | 3 | 0 | 0 | 0 | Urokinase-type plasminogen activator | BLOOD CLOTTING | UROK_HUMAN | Specifically cleave the zymogen plasminogen to form the active enzyme plasmin. |
| 1s95_v | 4 | 2.675 | 0.6688 | -0.57505 | 3 | 0 | 1 | 0 | 0 | 0 | Serine/threonine-protein phosphatase 5 | NONE | P53041 | Involved in hydrolase activity |
| 1jj9_v | 5 | 3.317 | 0.6635 | 1.2847 | 2 | 2 | 0 | 1 | 0 | 0 | Neutrophil collagenase | HYDROLASE | MMP8_HUMAN | Can degrade fibrillar type I, II, and III collagens. |
| 3h30_v | 4 | 2.638 | 0.6594 | -0.09594 | 2 | 0 | 2 | 0 | 0 | 0 | Casein kinase II subunit alpha | NONE | P68400 | Involved in protein kinase activity |
| 1csb_v | 4 | 2.59 | 0.6476 | -0.74383 | 1 | 3 | 0 | 0 | 0 | 0 | Cathepsin B | HYDROLASE (THIOL PROTEASE) | CATB_HUMAN | Thiol protease which is believed to participate in intracellular degradation and turnover of proteins. Has also been implicated in tumor invasion and metastasis. |
| 1pl6_v | 5 | 3.228 | 0.6455 | 0.430252 | 2 | 3 | 0 | 0 | 0 | 0 | Sorbitol dehydrogenase | OXIDOREDUCTASE | DHSO_HUMAN | NONE |
| 3cho_v | 4 | 2.575 | 0.6438 | -0.18211 | 2 | 1 | 0 | 1 | 0 | 0 | Leukotriene A-4 hydrolase | HYDROLASE | LKHA4_HUMAN | Hydrolyzes an epoxide moiety of leukotriene A4 (LTA-4) to form leukotriene B4 (LTB-4). The enzyme also has some peptidase activity. |
| 1le6_v | 4 | 2.485 | 0.6213 | -0.90672 | 2 | 1 | 1 | 0 | 0 | 0 | Group 10 secretory phospholipase A2 | NONE | O15496 | Involved in phospholipase A2 activity |
| 1b3o_v | 5 | 3.1 | 0.6201 | 0.288769 | 0 | 3 | 1 | 0 | 1 | 0 | Inosine-5-monophosphate dehydrogenase 2 | DEHYDROGENASE | IMDH2_HUMAN | Rate limiting enzyme in the de novo synthesis of guanine nucleotides and therefore is involved in the regulation of cell growth. It may also have a role in the development of malignancy and the growth progression of some tumors. |
| 1lzj_v | 6 | 3.698 | 0.6164 | 2.64944 | 2 | 2 | 2 | 0 | 0 | 0 | B transferase | NONE | Q9NY01 | Involved in transferase activity, transferring hexosyl groups |
| 3f0r_v | 5 | 3.078 | 0.6157 | 0.215499 | 4 | 0 | 1 | 0 | 0 | 0 | Histone deacetylase 8 | NONE | Q9BY41 | Chromatin structure and dynamics |
| 1m51_v | 6 | 3.668 | 0.6113 | 0.685644 | 3 | 3 | 0 | 0 | 0 | 0 | Phosphoenolpyruvate carboxykinase, cytosolic [GTP] | NONE | P35558 | Energy production and conversion |
| 1t7v_v | 4 | 2.433 | 0.6083 | -0.64852 | 0 | 3 | 1 | 0 | 0 | 0 | Zinc-alpha-2-glycoprotein | LIPID BINDING PROTEIN | ZA2G_HUMAN | Stimulates lipid degradation in adipocytes and causes the extensive fat losses associated with some advanced cancers. May bind polyunsaturated fatty acids. |
| 1r7t_v | 6 | 3.641 | 0.6068 | 2.12227 | 3 | 1 | 2 | 0 | 0 | 0 | Histo-blood group ABO system transferase | NONE | P16442 | Involved in transferase activity, transferring hexosyl groups |
| 1o1v_v | 7 | 4.241 | 0.6058 | 2.07617 | 5 | 1 | 1 | 0 | 0 | 0 | Gastrotropin | LIPID BINDING PROTEIN | FABP6_HUMAN | Ileal protein which stimulates gastric acid and pepsinogen secretion. Seems to be able to bind to bile salts and bilirubins. |
| 1xbb_v | 6 | 3.632 | 0.6053 | 1.28096 | 5 | 0 | 1 | 0 | 0 | 0 | Tyrosine-protein kinase SYK | TRANSFERASE | KSYK_HUMAN | Positive effector of BCR-stimulated responses. Couples the B-cell antigen receptor (BCR) to the mobilization of calcium ion either through a phosphoinositide 3-kinase-dependent pathway, when not phosphorylated on tyrosines of the linker region, or through |
| 1xh4_v | 8 | 4.828 | 0.6035 | 4.9064 | 4 | 1 | 2 | 1 | 0 | 0 | cAMP-dependent protein kinase catalytic subunit alpha | NONE | P00517 | Involved in protein serine/threonine kinase activity |
| 1fzv_v | 5 | 3.017 | 0.6034 | 0.27176 | 3 | 0 | 2 | 0 | 0 | 0 | Placenta growth factor | HORMONE/GROWTH FACTOR | PLGF_HUMAN | Growth factor active in angiogenesis, and endothelial cell growth, stimulating their proliferation and migration. It binds to receptor VEGFR-1/FLT1. PLGF-2 binds neuropilin-1 and 2 in a heparin-dependent manner. |
| 1wma_v | 5 | 3.014 | 0.6028 | 0.344384 | 2 | 2 | 1 | 0 | 0 | 0 | Carbonyl reductase [NADPH] 1 | OXIDOREDUCTASE | CBR1_HUMAN | Catalyzes the reduction of a wide variety of carbonyl compounds including the antitumor anthracycline antibiotics. Can convert prostaglandin E2 to prostaglandin F2-alpha. |
| 2i6a_v | 5 | 3 | 0.5999 | 0.650164 | 2 | 1 | 1 | 0 | 0 | 1 | Adenosine kinase | TRANSFERASE | ADK_HUMAN | ATP dependent phosphorylation of adenosine and other related nucleoside analogs to monophosphate derivatives. Serves as a potential regulator of concentrations of extracellular adenosine and intracellular adenine nucleotides. |
| 1u3v_v | 4 | 2.397 | 0.5993 | -1.57593 | 2 | 0 | 1 | 1 | 0 | 0 | Alcohol dehydrogenase 1B | OXIDOREDUCTASE | ADH1B_HUMAN | NONE |
| 1t84_v | 5 | 2.986 | 0.5972 | -0.43107 | 5 | 0 | 0 | 0 | 0 | 0 | Wiskott-Aldrich syndrome protein | SIGNALING PROTEIN | WASP_HUMAN | Effector protein for Rho-type GTPases, providing a link with the Arp2/3 complex that regulates the structure and dynamics of the actin cytoskeleton. Important for efficient actin polymerization. Possible regulator of lymphocyte and platelet function. |
| 1xdc_v | 5 | 2.981 | 0.5961 | 0.232402 | 2 | 1 | 2 | 0 | 0 | 0 | Superoxide dismutase [Mn], mitochondrial | NONE | P04179 | Destroys superoxide anion radicals which are normally produced within the cells and which are toxic to biological systems. |
| 1sg0_v | 5 | 2.977 | 0.5955 | 0.373899 | 2 | 2 | 0 | 0 | 0 | 1 | Ribosyldihydronicotinamide dehydrogenase [quinone] | OXIDOREDUCTASE | NQO2_HUMAN | The enzyme apparently serves as a quinone reductase in connection with conjugation reactions of hydroquinones involved in detoxification pathways as well as in biosynthetic processes such as the vitamin K-dependent gamma-carboxylation of glutamate residue |
| 1mq0_v | 5 | 2.97 | 0.594 | 0.223583 | 1 | 3 | 1 | 0 | 0 | 0 | Cytidine deaminase | HYDROLASE | CDD_HUMAN | This enzyme scavenge exogenous and endogenous cytidine and 2-deoxycytidine for UMP synthesis. |
| 3cpu_v | 5 | 2.968 | 0.5937 | 0.361147 | 0 | 2 | 3 | 0 | 0 | 0 | Pancreatic alpha-amylase | HYDROLASE | AMYP_HUMAN | NONE |
| 1p5z_v | 5 | 2.965 | 0.593 | 0.431417 | 1 | 1 | 3 | 0 | 0 | 0 | Deoxycytidine kinase | TRANSFERASE | DCK_HUMAN | Required for the phosphorylation of the deoxyribonucleosides deoxycytidine (dC), deoxyguanosine (dG) and deoxyadenosine (dA). It is also an essential enzyme for the phosphorylation of numerous nucleoside analogs widely employed as antiviral and chemothera |
| 1oec_v | 5 | 2.96 | 0.5919 | -0.21282 | 3 | 1 | 0 | 1 | 0 | 0 | Fibroblast growth factor receptor 2 | NONE | P21802 | Involved in protein kinase activity |
| 1rv1_v | 5 | 2.955 | 0.591 | -0.10151 | 4 | 0 | 1 | 0 | 0 | 0 | E3 ubiquitin-protein ligase Mdm2 | LIGASE | MDM2_HUMAN | Inhibits TP53/p53- and TP73/p73-mediated cell cycle arrest and apoptosis by binding its transcriptional activation domain. Functions as a ubiquitin ligase E3, in the presence of E1 and E2, toward p53 and itself. Permits the nuclear export of p53 and targe |
| 1m75_v | 4 | 2.359 | 0.5898 | -0.32779 | 1 | 1 | 2 | 0 | 0 | 0 | Hydroxyacyl-coenzyme A dehydrogenase, mitochondrial | NONE | Q16836 | Lipid transport and metabolism |
| 1p4f_v | 4 | 2.352 | 0.588 | -0.94817 | 1 | 2 | 1 | 0 | 0 | 0 | Death-associated protein kinase 1 | TRANSFERASE | DAPK1_HUMAN | Calcium/calmodulin-dependent serine/threonine kinase which acts as a positive regulator of apoptosis. |
| 1q22_v | 7 | 4.107 | 0.5867 | 1.7396 | 6 | 1 | 0 | 0 | 0 | 0 | Sulfotransferase family cytosolic 2B member 1 | NONE | O00204 | Involved in sulfotransferase activity |
| 1wok_v | 5 | 2.933 | 0.5866 | -0.18253 | 3 | 1 | 1 | 0 | 0 | 0 | Poly [ADP-ribose] polymerase 1 | NONE | P09874 | Involved in DNA binding |
| 1hkc_v | 5 | 2.924 | 0.5848 | 0.038731 | 0 | 3 | 2 | 0 | 0 | 0 | Hexokinase-1 | PHOSPHOTRANSFERASE | HXK1_HUMAN | NONE |
| 1q11_v | 5 | 2.923 | 0.5846 | 0.007189 | 2 | 1 | 1 | 1 | 0 | 0 | Tyrosyl-tRNA synthetase, cytoplasmic | NONE | P54577 | Translation, ribosomal structure and biogenesis |
| 1yk7_v | 5 | 2.923 | 0.5846 | 0.143231 | 2 | 3 | 0 | 0 | 0 | 0 | Cathepsin K | HYDROLASE | CATK_HUMAN | Closely involved in osteoclastic bone resorption and may participate partially in the disorder of bone remodeling. Displays potent endoprotease activity against fibrinogen at acid pH. May play an important role in extracellular matrix degradation. |
| 2rfn_v | 5 | 2.923 | 0.5845 | -0.07966 | 4 | 1 | 0 | 0 | 0 | 0 | Hepatocyte growth factor receptor | NONE | P08581 | Involved in hepatocyte growth factor activity |
| 1o6u_v | 5 | 2.922 | 0.5844 | -0.55979 | 5 | 0 | 0 | 0 | 0 | 0 | SEC14-like protein 2 | NONE | O76054 | Involved in transporter activity |
| 1dcy_v | 5 | 2.918 | 0.5836 | -0.52472 | 3 | 1 | 0 | 0 | 1 | 0 | Phospholipase A2, membrane associated | NONE | P14555 | Involved in phospholipase A2 activity |
| 2flr_v | 5 | 2.917 | 0.5834 | 0.045424 | 3 | 0 | 2 | 0 | 0 | 0 | Coagulation factor VII | HYDROLASE/BLOOD CLOTTING | FA7_HUMAN | Initiates the extrinsic pathway of blood coagulation. Serine protease that circulates in the blood in a zymogen form. Factor VII is converted to factor VIIa by factor Xa, factor XIIa, factor IXa, or thrombin by minor proteolysis. In the presence of tissue |
| 2opy_v | 5 | 2.911 | 0.5822 | 0.356956 | 1 | 2 | 0 | 1 | 0 | 1 | Baculoviral IAP repeat-containing protein 4 | APOPTOSIS ACTIVATOR | XIAP_HUMAN | Apoptotic suppressor. Has E3 ubiquitin-protein ligase activity. Mediates the proteasomal degradation of target proteins, such as caspase-3, SMAC or AIFM1. Inhibitor of caspase-3, -7 and -9. Mediates activation of MAP3K7/TAK1, leading to the activation of |
| 3blr_v | 5 | 2.911 | 0.5822 | 0.364934 | 2 | 2 | 0 | 1 | 0 | 0 | Cyclin-T1 | TRANSCRIPTION | CCNT1_HUMAN | Regulatory subunit of the cyclin-dependent kinase pair (CDK9/cyclin-T1) complex, also called positive transcription elongation factor B (P-TEFb), which is proposed to facilitate the transition from abortive to productive elongation by phosphorylating the |
| 2shp_v | 5 | 2.911 | 0.5821 | -0.63359 | 4 | 0 | 0 | 1 | 0 | 0 | Tyrosine-protein phosphatase non-receptor type 11 | TYROSINE PHOSPHATASE | PTN11_HUMAN | Acts downstream of various receptor and cytoplasmic protein tyrosine kinases to participate in the signal transduction from the cell surface to the nucleus. |
| 1fe0_v | 5 | 2.899 | 0.5798 | 0.345662 | 0 | 2 | 3 | 0 | 0 | 0 | Copper transport protein ATOX1 | NONE | O00244 | Involved in metal ion binding |
| 3bbt_v | 6 | 3.472 | 0.5786 | 1.20375 | 5 | 1 | 0 | 0 | 0 | 0 | Receptor tyrosine-protein kinase erbB-4 | TRANSFERASE | ERBB4_HUMAN | Specifically binds and is activated by neuregulins, NRG- 2, NRG-3, heparin-binding EGF-like growth factor, betacellulin and NTAK. Interaction with these factors induces cell differentiation. Not activated by EGF, TGF-A, and amphiregulin. |
| 1u3w_v | 5 | 2.892 | 0.5783 | -0.30981 | 3 | 1 | 0 | 1 | 0 | 0 | Alcohol dehydrogenase 1C | NONE | P00326 | Energy production and conversion |
| 1j4i_v | 7 | 4.044 | 0.5777 | 2.8614 | 3 | 4 | 0 | 0 | 0 | 0 | Peptidyl-prolyl cis-trans isomerase FKBP1A | ISOMERASE | FKB1A_HUMAN | May play a role in modulation of ryanodine receptor isoform-1 (RYR-1), a component of the calcium release channel of skeletal muscle sarcoplasmic reticulum. There are four molecules of FKBP12 per skeletal muscle RYR. PPIases accelerate the folding of prot |
| 1u4d_v | 5 | 2.876 | 0.5752 | -0.038 | 0 | 2 | 3 | 0 | 0 | 0 | Activated CDC42 kinase 1 | TRANSFERASE | ACK1_HUMAN | Downstream effector of CDC42 which mediates CDC42- dependent cell migration via phosphorylation of BCAR1. Binds to both poly- and mono-ubiquitin and regulates ligand-induced degradation of EGFR. Participates in clathrin-mediated endocytosis. May be involv |
| 1a7a_v | 5 | 2.874 | 0.5748 | -0.46169 | 0 | 3 | 2 | 0 | 0 | 0 | Adenosylhomocysteinase | HYDROLASE | SAHH_HUMAN | Adenosylhomocysteine is a competitive inhibitor of S- adenosyl-L-methionine-dependent methyl transferase reactions; therefore adenosylhomocysteinase may play a key role in the control of methylations via regulation of the intracellular concentration of ad |
| 2q6c_v | 5 | 2.866 | 0.5732 | -0.22591 | 2 | 2 | 0 | 0 | 1 | 0 | 3-hydroxy-3-methylglutaryl-coenzyme A reductase | OXIDOREDUCTASE | HMDH_HUMAN | This transmembrane glycoprotein is involved in the control of cholesterol biosynthesis. It is the rate-limiting enzyme of sterol biosynthesis. |
| 1qdd_v | 5 | 2.86 | 0.572 | 0.046101 | 0 | 4 | 1 | 0 | 0 | 0 | Lithostathine-1-alpha | NONE | P05451 | Involved in sugar binding |
| 1mmq_v | 5 | 2.857 | 0.5715 | -0.12301 | 1 | 2 | 2 | 0 | 0 | 0 | Matrilysin | METALLOPROTEASE | MMP7_HUMAN | Degrades casein, gelatins of types I, III, IV, and V, and fibronectin. Activates procollagenase. |
| 1vj5_v | 5 | 2.853 | 0.5706 | -0.40527 | 3 | 1 | 1 | 0 | 0 | 0 | Epoxide hydrolase 2 | HYDROLASE | HYES_HUMAN | Acts on epoxides (alkene oxides, oxiranes) and arene oxides. Plays a role in xenobiotic metabolism by degrading potentially toxic epoxides. Also determines steady-state levels of physiological mediators. Has low phosphatase activity. |
| 1gse_v | 6 | 3.417 | 0.5695 | 0.717115 | 5 | 1 | 0 | 0 | 0 | 0 | Glutathione S-transferase A1 | NONE | P08263 | Involved in glutathione transferase activity |
| 1he2_v | 6 | 3.395 | 0.5658 | 1.17762 | 4 | 1 | 0 | 0 | 1 | 0 | Flavin reductase | NONE | P30043 | Cell wall/membrane/envelope biogenesis |
| 1sm2_v | 6 | 3.383 | 0.5639 | 0.669031 | 4 | 2 | 0 | 0 | 0 | 0 | Tyrosine-protein kinase ITK/TSK | NONE | Q08881 | Involved in protein kinase activity |
| 1e8z_v | 5 | 2.819 | 0.5639 | -0.56075 | 3 | 1 | 0 | 1 | 0 | 0 | Phosphatidylinositol-4,5-bisphosphate 3-kinase catalytic subunit gamma isoform | PHOSPHOINOSITIDE 3-KINASE GAMMA | PK3CG_HUMAN | 3-phosphorylates the cellular phosphoinositide PtdIns- 4,5-biphosphate (PtdIns(4,5)P2) to produce PtdIns-3, 4,5- triiphosphate (PtdIns(3,4,5)P3). Links G-protein coupled receptor activation to the secondary messenger PtdIns(3,4,5)P3 production. |
| 2f57_v | 5 | 2.814 | 0.5629 | -0.12325 | 3 | 1 | 1 | 0 | 0 | 0 | Serine/threonine-protein kinase PAK 7 | TRANSFERASE | PAK7_HUMAN | The activated kinase acts on a variety of targets (By similarity). |
| 1h6g_v | 5 | 2.81 | 0.5619 | -0.33349 | 3 | 2 | 0 | 0 | 0 | 0 | Catenin alpha-1 | CYTOSKELETON | CTNA1_HUMAN | Associates with the cytoplasmic domain of a variety of cadherins. The association of catenins to cadherins produces a complex which is linked to the actin filament network, and which seems to be of primary importance for cadherins cell-adhesion properties |
| 3e8r_v | 5 | 2.809 | 0.5618 | 0.275323 | 2 | 1 | 1 | 0 | 0 | 1 | Disintegrin and metalloproteinase domain-containing protein 17 | NONE | P78536 | Cleaves the membrane-bound precursor of TNF-alpha to its mature soluble form. Responsible for the proteolytical release of soluble JAM3 from endothelial cells surface. Responsible for the proteolytic release of several other cell-surface proteins, including p75 TNF-receptor, interleukin 1 receptor type II, p55 TNF-receptor, transforming growth factor-alpha, L-selectin, growth hormone receptor, MUC1 and the amyloid precursor protein. Acts as an activator of Notch pathway by mediating cleavage of Notch, generating the membrane-associated intermediate fragment called Notch extracellular truncation (NEXT). Plays a role in the proteolytic processing of ACE2. |
| 1oix_v | 6 | 3.365 | 0.5608 | 1.24427 | 0 | 2 | 4 | 0 | 0 | 0 | Ras-related protein Rab-11A | PROTEIN TRANSPORT | RB11A_HUMAN | Modulates endosomal trafficking (By similarity). |
| 1qcf_v | 7 | 3.918 | 0.5597 | 1.6736 | 3 | 3 | 1 | 0 | 0 | 0 | Tyrosine-protein kinase HCK | TYROSINE KINASE | HCK_HUMAN | May serve as part of a signaling pathway coupling the Fc receptor to the activation of the respiratory burst. May also contribute to neutrophil migration and may regulate the degranulation process of neutrophils. |
| 1svh_v | 7 | 3.906 | 0.5581 | 2.5173 | 4 | 1 | 1 | 1 | 0 | 0 | cAMP-dependent protein kinase, alpha-catalytic subunit | NONE | P00517 | Involved in protein serine/threonine kinase activity |
| 1mkp_v | 4 | 2.23 | 0.5574 | -1.6731 | 3 | 0 | 1 | 0 | 0 | 0 | Dual specificity protein phosphatase 6 | HYDROLASE | DUS6_HUMAN | Inactivates MAP kinases. Has a specificity for the ERK family. |
| 2rnf_v | 5 | 2.773 | 0.5547 | -0.46235 | 0 | 3 | 1 | 0 | 1 | 0 | Ribonuclease 4 | NONE | P34096 | Involved in nucleic acid binding |
| 1utt_v | 5 | 2.748 | 0.5496 | -0.71462 | 2 | 3 | 0 | 0 | 0 | 0 | Macrophage metalloelastase | HYDROLASE | MMP12_HUMAN | May be involved in tissue injury and remodeling. Has significant elastolytic activity. Can accept large and small amino acids at the P1 site, but has a preference for leucine. Aromatic or hydrophobic residues are preferred at the P1 site, with small hydr |
| 3bej_v | 7 | 3.839 | 0.5485 | 2.14647 | 4 | 1 | 1 | 0 | 1 | 0 | Bile acid receptor | TRANSCRIPTION REGULATOR | NR1H4_HUMAN | Receptor for bile acids such as chenodeoxycholic acid, lithocholic acid and deoxycholic acid. Represses the transcription of the cholesterol 7-alpha-hydroxylase gene (CYP7A1) and activates the intestinal bile acid-binding protein (IBABP). Activates the tr |
| 1kmq_v | 5 | 2.741 | 0.5481 | -0.19348 | 0 | 3 | 2 | 0 | 0 | 0 | Transforming protein RhoA | NONE | P61586 | Involved in GTP binding |
| 1oiz_v | 5 | 2.725 | 0.545 | -0.87068 | 4 | 1 | 0 | 0 | 0 | 0 | Alpha-tocopherol transfer protein | NONE | P49638 | Involved in vitamin E transporter activity |
| 1ihi_v | 7 | 3.807 | 0.5439 | 0.570639 | 3 | 2 | 1 | 0 | 1 | 0 | Aldo-keto reductase family 1 member C2 | OXIDOREDUCTASE | AK1C2_HUMAN | Works in concert with the 5-alpha/5-beta-steroid reductases to convert steroid hormones into the 3-alpha/5-alpha and 3-alpha/5-beta-tetrahydrosteroids. Catalyzes the inactivation of the most potent androgen 5-alpha-dihydrotestosterone (5-alpha- DHT) to 5- |
| 1fe3_v | 7 | 3.804 | 0.5434 | 0.979681 | 6 | 0 | 0 | 0 | 1 | 0 | Fatty acid-binding protein, brain | LIPID BINDING PROTEIN | FABP7_HUMAN | B-FABP could be involved in the transport of a so far unknown hydrophobic ligand with potential morphogenic activity during CNS development. It is required for the establishment of the radial glial fiber system in developing brain, a system that is necess |
| 1fo2_v | 5 | 2.706 | 0.5413 | -0.30152 | 0 | 2 | 2 | 1 | 0 | 0 | Endoplasmic reticulum mannosyl-oligosaccharide 1,2-alpha-mannosidase | NONE | Q9UKM7 | Involved in mannosyl-oligosaccharide 1,2-alpha-mannosidase activity |
| 2vd1_v | 6 | 3.247 | 0.5411 | 0.238634 | 5 | 1 | 0 | 0 | 0 | 0 | Glutathione-requiring prostaglandin D synthase | ISOMERASE | PTGD2_HUMAN | Catalyzes the conversion of PGH2 to PGD2, a prostaglandin involved in smooth muscle contraction/relaxation and a potent inhibitor of platelet aggregation. |
| 2c47_v | 4 | 2.163 | 0.5407 | -1.64541 | 1 | 1 | 2 | 0 | 0 | 0 | Casein kinase I isoform gamma-2 | TRANSFERASE | KC1G2_HUMAN | Casein kinases are operationally defined by their preferential utilization of acidic proteins such as caseins as substrates. It can phosphorylate a large number of proteins. Participates in Wnt signaling (By similarity). |
| 2oo8_v | 8 | 4.303 | 0.5378 | 2.41347 | 5 | 1 | 1 | 1 | 0 | 0 | Angiopoietin-1 receptor | TRANSFERASE | TIE2_HUMAN | This protein is a protein tyrosine-kinase transmembrane receptor for angiopoietin 1. It may constitute the earliest mammalian endothelial cell lineage marker. Probably regulates endothelial cell proliferation, differentiation and guides the proper pattern |
| 1pkg_v | 7 | 3.756 | 0.5366 | 2.2206 | 0 | 2 | 3 | 0 | 2 | 0 | Mast/stem cell growth factor receptor | TRANSFERASE ACTIVATOR | KIT_HUMAN | This is the receptor for stem cell factor (mast cell growth factor). It has a tyrosine-protein kinase activity. Binding of the ligands leads to the autophosphorylation of KIT and its association with substrates such as phosphatidylinositol 3-kinase (Pi3K) |
| 1of7_v | 5 | 2.669 | 0.5338 | -0.56996 | 2 | 1 | 2 | 0 | 0 | 0 | Aldehyde dehydrogenase, mitochondrial | OXIDOREDUCTASE | ALDH2_HUMAN | NONE |
| 1hrk_v | 8 | 4.244 | 0.5305 | 2.23143 | 5 | 1 | 2 | 0 | 0 | 0 | Ferrochelatase, mitochondrial | NONE | P22830 | Coenzyme transport and metabolism |
| 1hmt_v | 7 | 3.7 | 0.5286 | 0.944218 | 5 | 1 | 0 | 0 | 1 | 0 | Fatty acid-binding protein, heart | LIPID-BINDING PROTEIN | FABPH_HUMAN | FABP are thought to play a role in the intracellular transport of long-chain fatty acids and their acyl-CoA esters. |
| 1m6w_v | 5 | 2.643 | 0.5285 | -0.94646 | 3 | 0 | 1 | 0 | 1 | 0 | Alcohol dehydrogenase class-3 | OXIDOREDUCTASE | ADHX_HUMAN | Class-III ADH is remarkably ineffective in oxidizing ethanol, but it readily catalyzes the oxidation of long-chain primary alcohols and the oxidation of S-(hydroxymethyl) glutathione. |
| 1jbq_v | 7 | 3.684 | 0.5263 | 0.871799 | 2 | 4 | 0 | 0 | 1 | 0 | Cystathionine beta-synthase | NONE | P35520 | Amino acid transport and metabolism |
| 1imx_v | 7 | 3.664 | 0.5235 | 1.38318 | 4 | 1 | 2 | 0 | 0 | 0 | Insulin-like growth factor IA | NONE | P01343 | NONE |
| 1isj_v | 7 | 3.63 | 0.5185 | 2.16839 | 1 | 5 | 1 | 0 | 0 | 0 | ADP-ribosyl cyclase 2 | HYDROLASE | BST1_HUMAN | Synthesizes cyclic ADP-ribose, a second messenger that elicits calcium release from intracellular stores. May be involved in pre-B-cell growth. |
| 1og5_v | 6 | 3.105 | 0.5175 | 0.20794 | 3 | 3 | 0 | 0 | 0 | 0 | Cytochrome P450 2C9 | ELECTRON TRANSPORT | CP2C9_HUMAN | Cytochromes P450 are a group of heme-thiolate monooxygenases. In liver microsomes, this enzyme is involved in an NADPH-dependent electron transport pathway. It oxidizes a variety of structurally unrelated compounds, including steroids, fatty acids, and xe |
| 1uej_v | 6 | 3.095 | 0.5158 | 0.439058 | 1 | 3 | 2 | 0 | 0 | 0 | Uridine-cytidine kinase 2 | NONE | Q9BZX2 | Nucleotide transport and metabolism |
| 1yvj_v | 6 | 3.086 | 0.5144 | -0.08222 | 4 | 1 | 1 | 0 | 0 | 0 | Tyrosine-protein kinase JAK3 | TRANSFERASE | JAK3_HUMAN | Tyrosine kinase of the non-receptor type, involved in the interleukin-2 and interleukin-4 signaling pathway. Phosphorylates STAT6, IRS1, IRS2 and PI3K. |
| 1xtq_v | 5 | 2.56 | 0.5121 | -0.5855 | 0 | 3 | 2 | 0 | 0 | 0 | GTP-binding protein Rheb | NONE | Q15382 | Activates the protein kinase activity of mTORC1, and thereby plays a role in the regulation of apoptosis. Stimulates the phosphorylation of S6K1 and EIF4EBP1 through activation of mTORC1 signaling. Has low intrinsic GTPase activity. |
| 1ljr_v | 7 | 3.543 | 0.5061 | 1.36601 | 1 | 3 | 1 | 1 | 1 | 0 | Glutathione S-transferase theta-2 | TRANSFERASE | GSTT2_HUMAN | Conjugation of reduced glutathione to a wide number of exogenous and endogenous hydrophobic electrophiles. Has a sulfatase activity. |
| 1irj_v | 7 | 3.543 | 0.5061 | 1.41779 | 4 | 0 | 3 | 0 | 0 | 0 | Protein S100-A9 | METAL BINDING PROTEIN | S10A9_HUMAN | Expressed by macrophages in acutely inflammated tissues and in chronic inflammations. Seem to be an inhibitor of protein kinases. Also expressed in epithelial cells constitutively or induced during dermatoses. May interact with components of the intermedi |
| 1ore_v | 7 | 3.523 | 0.5032 | 1.16357 | 0 | 4 | 3 | 0 | 0 | 0 | Adenine phosphoribosyltransferase | NONE | P07741 | Nucleotide transport and metabolism |
| 2bu5_v | 6 | 2.991 | 0.4986 | -0.50584 | 5 | 1 | 0 | 0 | 0 | 0 | [Pyruvate dehydrogenase [lipoamide]] kinase isozyme 2, mitochondrial | TRANSFERASE | PDK2_HUMAN | Inhibits the mitochondrial pyruvate dehydrogenase complex by phosphorylation of the E1 alpha subunit, thus contributing to the regulation of glucose metabolism. |
| 1uzf_v | 7 | 3.475 | 0.4964 | 0.374157 | 2 | 3 | 0 | 0 | 2 | 0 | Angiotensin-converting enzyme | METALLOPROTEASE | ACE_HUMAN | Converts angiotensin I to angiotensin II by release of the terminal His-Leu, this results in an increase of the vasoconstrictor activity of angiotensin. Also able to inactivate bradykinin, a potent vasodilator. Has also a glycosidase activity which releas |
| 1p2u_v | 7 | 3.456 | 0.4937 | 1.65556 | 0 | 3 | 3 | 0 | 1 | 0 | GTPase HRas | SIGNALING PROTEIN | RASH_HUMAN | Ras proteins bind GDP/GTP and possess intrinsic GTPase activity. |
| 1dia_v | 6 | 2.961 | 0.4934 | 0.027301 | 2 | 2 | 2 | 0 | 0 | 0 | C-1-tetrahydrofolate synthase, cytoplasmic | NONE | P11586 | Nucleotide transport and metabolism |
| 1xvp_v | 6 | 2.957 | 0.4928 | -0.44112 | 6 | 0 | 0 | 0 | 0 | 0 | Nuclear receptor subfamily 1 group I member 3 | DNA BINDING PROTEIN | NR1I3_HUMAN | Binds and transactivates the retinoic acid response elements that control expression of the retinoic acid receptor beta 2 and alcohol dehydrogenase 3 genes. Transactivates both the phenobarbital responsive element module of the human CYP2B6 gene and the C |
| 1m4b_v | 6 | 2.943 | 0.4904 | 0.066287 | 1 | 2 | 2 | 1 | 0 | 0 | Interleukin-2 | CYTOKINE | IL2_HUMAN | Produced by T-cells in response to antigenic or mitogenic stimulation, this protein is required for T-cell proliferation and other activities crucial to regulation of the immune response. Can stimulate B-cells, monocytes, lymphokine- activated killer cell |
| 1tjj_v | 6 | 2.942 | 0.4904 | -0.09042 | 4 | 1 | 0 | 1 | 0 | 0 | Ganglioside GM2 activator | NONE | P17900 | Involved in sphingolipid activator protein activity |
| 1i7g_v | 6 | 2.942 | 0.4904 | -0.42981 | 4 | 2 | 0 | 0 | 0 | 0 | Peroxisome proliferator-activated receptor alpha | TRANSCRIPTION | PPARA_HUMAN | Receptor that binds peroxisome proliferators such as hypolipidemic drugs and fatty acids. Once activated by a ligand, the receptor binds to a promoter element in the gene for acyl-CoA oxidase and activates its transcription. It therefore controls the pero |
| 6fit_v | 6 | 2.941 | 0.4902 | 0.285417 | 0 | 4 | 2 | 0 | 0 | 0 | Bis(5-adenosyl)-triphosphatase | NONE | P49789 | Nucleotide transport and metabolism |
| 1h1b_v | 6 | 2.939 | 0.4898 | -0.24116 | 3 | 3 | 0 | 0 | 0 | 0 | Leukocyte elastase | HYDROLASE(SERINE PROTEASE) | ELNE_HUMAN | Modifies the functions of natural killer cells, monocytes and granulocytes. Inhibits C5a-dependent neutrophil enzyme release and chemotaxis. |
| 4ald_v | 6 | 2.938 | 0.4897 | -0.25562 | 0 | 4 | 1 | 0 | 1 | 0 | Fructose-bisphosphate aldolase A | LYASE | ALDOA_HUMAN | NONE |
| 2hpa_v | 6 | 2.937 | 0.4896 | -0.19262 | 1 | 2 | 2 | 0 | 1 | 0 | Prostatic acid phosphatase | HYDROLASE | PPAP_HUMAN | NONE |
| 1c9y_v | 5 | 2.447 | 0.4894 | -1.6158 | 0 | 2 | 1 | 0 | 2 | 0 | Ornithine carbamoyltransferase, mitochondrial | NONE | P00480 | Amino acid transport and metabolism |
| 1qab_v | 6 | 2.935 | 0.4892 | -0.42196 | 5 | 0 | 1 | 0 | 0 | 0 | Retinol-binding protein 4 | TRANSPORT PROTEIN | RET4_HUMAN | Delivers retinol from the liver stores to the peripheral tissues. In plasma, the RBP-retinol complex interacts with transthyretin, this prevents its loss by filtration through the kidney glomeruli. |
| 1n6i_v | 6 | 2.93 | 0.4884 | 0.123107 | 0 | 2 | 4 | 0 | 0 | 0 | Ras-related protein Rab-5A | PROTEIN TRANSPORT | RAB5A_HUMAN | Required for the fusion of plasma membranes and early endosomes. |
| 2uw9_v | 6 | 2.929 | 0.4881 | 0.0905 | 3 | 1 | 1 | 1 | 0 | 0 | RAC-beta serine/threonine-protein kinase | TRANSFERASE/INHIBITOR COMPLEX | AKT2_HUMAN | General protein kinase capable of phosphorylating several known proteins. |
| 1hti_v | 6 | 2.928 | 0.488 | -0.42987 | 1 | 3 | 0 | 0 | 2 | 0 | Triosephosphate isomerase | ISOMERASE(INTRAMOLECULAR | TPIS_HUMAN | NONE |
| 1y0x_v | 6 | 2.922 | 0.487 | -0.01112 | 4 | 0 | 1 | 0 | 1 | 0 | Thyroid hormone receptor beta-2 | NONE | P37243 | Involved in transcription factor activity |
| 1xjd_v | 7 | 3.407 | 0.4868 | 0.60935 | 4 | 1 | 1 | 1 | 0 | 0 | Protein kinase C theta type | TRANSFERASE | KPCT_HUMAN | This is a calcium-independent, phospholipid-dependent, serine- and threonine-specific enzyme. Essential for T-cell receptor (TCR)-mediated T-cell activation, but is dispensable during TCR-dependent thymocyte development. Links the TCR signaling complex to;PKC is activated by diacylglycerol which in turn phosphorylates a range of cellular proteins. PKC also serves as the receptor for phorbol esters, a class of tumor promoters. |
| 3f7h_v | 6 | 2.921 | 0.4868 | 0.010881 | 2 | 2 | 1 | 1 | 0 | 0 | Baculoviral IAP repeat-containing protein 7 | NONE | Q96CA5 | Apoptotic regulator capable of exerting proapoptotic and anti-apoptotic activities and plays crucial roles in apoptosis, cell proliferation, and cell cycle control. Its anti-apoptotic activity is mediated through the inhibition of CASP3, CASP7 and CASP9, as well as by its E3 ubiquitin-protein ligase activity. As it is a weak caspase inhibitor, its anti-apoptotic activity is thought to be due to its ability to ubiquitinate DIABLO/SMAC targeting it for degradation thereby promoting cell survival. May contribute to caspase inhibition, by blocking the ability of DIABLO/SMAC to disrupt XIAP/BIRC4-caspase interactions. Protects against apoptosis induced by TNF or by chemical agents such as adriamycin, etoposide or staurosporine. Suppression of apoptosis is mediated by activation of MAPK8/JNK1, and possibly also of MAPK9/JNK2. This activation depends on TAB1 and NR2C2/TAK1. In vitro, inhibits CASP3 and proteolytic activation of pro-CASP9. Isoform 1 blocks staurosporine-induced apoptosis. Isoform 2 blocks etoposide-induced apoptosis. Isoform 2 protects against natural killer (NK) cell killing whereas isoform 1 augments killing. |
| 1itu_v | 8 | 3.891 | 0.4864 | 1.07516 | 4 | 2 | 1 | 0 | 1 | 0 | Dipeptidase 1 | HYDROLASE | DPEP1_HUMAN | Hydrolyzes a wide range of dipeptides. Implicated in the renal metabolism of glutathione and its conjugates. Converts leukotriene D4 to leukotriene E4; it may play an important role in the regulation of leukotriene activity. |
| 1uhl_v | 6 | 2.917 | 0.4861 | -0.55297 | 5 | 1 | 0 | 0 | 0 | 0 | Oxysterols receptor LXR-alpha | DNA BINDING PROTEIN | NR1H3_HUMAN | Orphan receptor. Interaction with RXR shifts RXR from its role as a silent DNA-binding partner to an active ligand- binding subunit in mediating retinoid responses through target genes defined by LXRES. LXRES are DR4-type response elements characterized b |
| 1dyt_v | 6 | 2.914 | 0.4857 | -0.45461 | 0 | 5 | 1 | 0 | 0 | 0 | Eosinophil cationic protein | NONE | P12724 | Involved in nucleic acid binding |
| 1mfv_v | 6 | 2.912 | 0.4853 | 0.056896 | 0 | 2 | 4 | 0 | 0 | 0 | Salivary alpha-amylase | NONE | P04745 | Carbohydrate transport and metabolism |
| 1sz7_v | 6 | 2.911 | 0.4851 | -0.56896 | 5 | 1 | 0 | 0 | 0 | 0 | Trafficking protein particle complex subunit 3 | NONE | O43617 | May play a role in vesicular transport from endoplasmic reticulum to Golgi |
| 1i3l_v | 6 | 2.909 | 0.4848 | -0.12186 | 0 | 3 | 1 | 0 | 1 | 1 | UDP-glucose 4-epimerase | ISOMERASE | GALE_HUMAN | Catalyzes two distinct but analogous reactions: the epimerization of UDP-glucose to UDP-galactose and the epimerization of UDP-N-acetylglucosamine to UDP-N- acetylgalactosamine. |
| 1iri_v | 7 | 3.389 | 0.4841 | 0.23438 | 0 | 6 | 1 | 0 | 0 | 0 | Glucose-6-phosphate isomerase | ISOMERASE | G6PI_HUMAN | Neurotrophic factor for spinal and sensory neurons. |
| 1o5f_v | 5 | 2.419 | 0.4837 | -1.09866 | 2 | 1 | 1 | 1 | 0 | 0 | Serine protease hepsin | NONE | P05981 | Plays an essential role in cell growth and maintenance of cell morphology |
| 1u59_v | 7 | 3.381 | 0.483 | 0.636276 | 4 | 1 | 2 | 0 | 0 | 0 | Tyrosine-protein kinase ZAP-70 | TRANSFERASE | ZAP70_HUMAN | Plays a role in T-cell development and lymphocyte activation. Essential for TCR-mediated IL-2 production. Isoform 1 induces TCR-mediated signal transduction, isoform 2 does not. |
| 1iz2_v | 6 | 2.897 | 0.4829 | -0.23856 | 3 | 1 | 2 | 0 | 0 | 0 | Alpha-1-antitrypsin | PROTEIN BINDING | A1AT_HUMAN | Inhibitor of serine proteases. Its primary target is elastase, but it also has a moderate affinity for plasmin and thrombin. Inhibits trypsin, chymotrypsin and plasminogen activator. The aberrant form inhibits insulin-induced NO synthesis in platelets, de |
| 1bj4_v | 6 | 2.891 | 0.4818 | -0.07499 | 2 | 4 | 0 | 0 | 0 | 0 | Serine hydroxymethyltransferase, cytosolic | TRANSFERASE | GLYC_HUMAN | Interconversion of serine and glycine. |
| 3d7t_v | 6 | 2.889 | 0.4815 | -0.19674 | 3 | 1 | 1 | 1 | 0 | 0 | Tyrosine-protein kinase CSK | TRANSFERASE | CSK_HUMAN | Specifically phosphorylates Tyr-504 on LCK, which acts as a negative regulatory site. Can also act on the LYN and FYN kinases. |
| 1v84_v | 6 | 2.887 | 0.4812 | -0.03161 | 0 | 3 | 3 | 0 | 0 | 0 | Galactosylgalactosylxylosylprotein 3-beta-glucuronosyltransferase 1 | TRANSFERASE | B3GA1_HUMAN | Involved in the biosynthesis of L2/HNK-1 carbohydrate epitope on glycoproteins. Can also play a role in glycosaminoglycan biosynthesis. Substrates include asialo- orosomucoid (ASOR), asialo-fetuin, and asialo-neural cell adhesion molecule. Requires sphing |
| 2nsi_v | 6 | 2.887 | 0.4811 | 0.091499 | 1 | 1 | 4 | 0 | 0 | 0 | Nitric oxide synthase, inducible | OXIDOREDUCTASE | NOS2_HUMAN | Produces nitric oxide (NO) which is a messenger molecule with diverse functions throughout the body. In macrophages, NO mediates tumoricidal and bactericidal actions. |
| 1w7n_v | 6 | 2.885 | 0.4809 | -0.54976 | 1 | 2 | 1 | 1 | 1 | 0 | Kynurenine--oxoglutarate transaminase 1 | TRANSFERASE | KAT1_HUMAN | Catalyzes the irreversible transamination of the L- tryptophan metabolite L-kynurenine to form kynurenic acid (KA). Metabolizes the cysteine conjugates of certain halogenated alkenes and alkanes to form reactive metabolites. Catalyzes the beta- eliminatio |
| 1s9j_v | 9 | 4.301 | 0.4779 | 1.38858 | 7 | 2 | 0 | 0 | 0 | 0 | Dual specificity mitogen-activated protein kinase kinase 1 | TRANSFERASE | MP2K1_HUMAN | Catalyzes the concomitant phosphorylation of a threonine and a tyrosine residue in a Thr-Glu-Tyr sequence located in MAP kinases. Activates ERK1 and ERK2 MAP kinases. |
| 2qcf_v | 6 | 2.861 | 0.4768 | -0.15929 | 0 | 3 | 2 | 0 | 1 | 0 | Uridine 5-monophosphate synthase | LYASE | PYR5_HUMAN | NONE |
| 1v4s_v | 8 | 3.811 | 0.4764 | 1.1588 | 3 | 3 | 2 | 0 | 0 | 0 | Glucokinase | TRANSFERASE | HXK4_HUMAN | Catalyzes the initial step in utilization of glucose by the beta-cell and liver at physiological glucose concentration. Glucokinase has a high Km for glucose, and so it is effective only when glucose is abundant. The role of GCK is to provide G6P for the |
| 2c30_v | 6 | 2.854 | 0.4757 | -0.62805 | 0 | 4 | 1 | 0 | 1 | 0 | Serine/threonine-protein kinase PAK 6 | TRANSFERASE | PAK6_HUMAN | The activated kinase acts on a variety of targets (By similarity). |
| 1onq_v | 6 | 2.847 | 0.4745 | 0.203607 | 3 | 2 | 0 | 0 | 1 | 0 | T-cell surface glycoprotein CD1a | IMMUNE SYSTEM | CD1A_HUMAN | Antigen-presenting protein that binds self and non-self lipid and glycolipid antigens and presents them to T-cell receptors on natural killer T-cells. |
| 1hlc_v | 6 | 2.839 | 0.4731 | -0.28287 | 0 | 3 | 3 | 0 | 0 | 0 | Galectin-2 | LECTIN | LEG2_HUMAN | This protein binds beta-galactoside. Its physiological function is not yet known. |
| 1elv_v | 6 | 2.836 | 0.4726 | -0.46733 | 0 | 3 | 1 | 1 | 1 | 0 | Complement C1s subcomponent | HYDROLASE | C1S_HUMAN | C1s B chain is a serine protease that combines with C1q and C1s to form C1, the first component of the classical pathway of the complement system. C1r activates C1s so that it can, in turn, activate C2 and C4. |
| 1b56_v | 5 | 2.363 | 0.4726 | -1.86449 | 3 | 1 | 0 | 0 | 1 | 0 | Fatty acid-binding protein, epidermal | LIPID-BINDING | FABP5_HUMAN | High specificity for fatty acids. Highest affinity for C18 chain length. Decreasing the chain length or introducing double bonds reduces the affinity. May be involved in keratinocyte differentiation. |
| 1np0_v | 6 | 2.812 | 0.4687 | -0.13847 | 1 | 1 | 3 | 1 | 0 | 0 | Beta-hexosaminidase subunit beta | NONE | P07686 | Carbohydrate transport and metabolism |
| 1e2s_v | 6 | 2.8 | 0.4667 | -0.33916 | 1 | 2 | 2 | 0 | 1 | 0 | Arylsulfatase A | HYDROLASE | ARSA_HUMAN | Hydrolyzes cerebroside sulfate. |
| 1uwj_v | 7 | 3.264 | 0.4662 | 0.260335 | 5 | 1 | 1 | 0 | 0 | 0 | B-Raf proto-oncogene serine/threonine-protein kinase | KINASE | BRAF1_HUMAN | Involved in the transduction of mitogenic signals from the cell membrane to the nucleus. May play a role in the postsynaptic responses of hippocampal neuron. |
| 2gqg_v | 8 | 3.708 | 0.4635 | 1.60114 | 5 | 1 | 2 | 0 | 0 | 0 | Proto-oncogene tyrosine-protein kinase ABL1 | NONE | P00519 | Involved in protein kinase activity |
| 1wda_v | 6 | 2.778 | 0.463 | -0.68197 | 2 | 2 | 1 | 1 | 0 | 0 | Protein-arginine deiminase type-4 | HYDROLASE | PADI4_HUMAN | Catalyzes the citrullination/deimination of arginine residues of proteins. Citrullinates histone H3 at Arg-8 and/or Arg-17 and histone H4 at Arg-3, which prevents their methylation by CARM1 and HRMT1L2/PRMT1 and represses transcription. Citrullinate |
| 1wuu_v | 5 | 2.299 | 0.4598 | -1.10602 | 0 | 2 | 3 | 0 | 0 | 0 | Galactokinase | TRANSFERASE | GALK1_HUMAN | Major enzyme for galactose metabolism. |
| 2gv7_v | 6 | 2.755 | 0.4592 | -0.02765 | 1 | 2 | 1 | 2 | 0 | 0 | Suppressor of tumorigenicity protein 14 | HYDROLASE | ST14_HUMAN | Degrades extracellular matrix. Proposed to play a role in breast cancer invasion and metastasis. Exhibits trypsin-like activity as defined by cleavage of synthetic substrates with Arg or Lys as the P1 site. |
| 1g3m_v | 7 | 3.208 | 0.4583 | -0.01782 | 6 | 1 | 0 | 0 | 0 | 0 | Estrogen sulfotransferase | TRANSFERASE | ST1E1_HUMAN | May control the level of the estrogen receptor by sulfurylating free estradiol. Maximally sulfates beta-estradiol and estrone at concentrations of 20 nM. Also sulfates dehydroepiandrosterone, pregnenolone, ethinylestradiol, equalenin, diethylstilbesterol |
| 1tx4_v | 6 | 2.75 | 0.4583 | -0.32216 | 0 | 4 | 2 | 0 | 0 | 0 | Rho GTPase-activating protein 1 | NONE | Q07960 | GTPase activator for the Rho, Rac and Cdc42 proteins, converting them to the putatively inactive GDP-bound state. Cdc42 seems to be the preferred substrate. |
| 1l9n_v | 8 | 3.663 | 0.4579 | 0.587773 | 3 | 4 | 1 | 0 | 0 | 0 | Protein-glutamine gamma-glutamyltransferase E | TRANSFERASE | TGM3_HUMAN | Catalyzes the cross-linking of proteins and the conjugation of polyamines to proteins. It is responsible for the later stages of cell envelope formation in the epidermis and the hair follicle. |
| 1nhz_v | 8 | 3.656 | 0.457 | 0.553369 | 7 | 1 | 0 | 0 | 0 | 0 | Glucocorticoid receptor | HORMONE RECEPTOR | GCR_HUMAN | Receptor for glucocorticoids (GC). Has a dual mode of action: as a transcription factor that binds to glucocorticoid response elements (GRE) and as a modulator of other transcription factors. Affects inflammatory responses, cellular proliferation and diff |
| 1p5j_v | 7 | 3.169 | 0.4527 | -0.04318 | 2 | 4 | 0 | 0 | 1 | 0 | L-serine dehydratase | NONE | P20132 | Amino acid transport and metabolism |
| 1rem_v | 6 | 2.706 | 0.451 | -0.55748 | 0 | 3 | 3 | 0 | 0 | 0 | Lysozyme C | NONE | P61626 | Lysozymes have primarily a bacteriolytic function; those in tissues and body fluids are associated with the monocyte-macrophage system and enhance the activity of immunoagents. |
| 1egc_v | 7 | 3.138 | 0.4483 | 0.73686 | 3 | 2 | 2 | 0 | 0 | 0 | Medium-chain specific acyl-CoA dehydrogenase, mitochondrial | ELECTRON TRANSFER | ACADM_HUMAN | This enzyme is specific for acyl chain lengths of 4 to 16. |
| 2b7a_v | 7 | 3.137 | 0.4481 | -0.09545 | 3 | 2 | 2 | 0 | 0 | 0 | Tyrosine-protein kinase JAK2 | TRANSFERASE | JAK2_HUMAN | Plays a role in leptin signaling and control of body weight (By similarity). Tyrosine kinase of the non-receptor type, involved in interleukin-3 and probably interleukin-23 signal transduction. |
| 1mqb_v | 7 | 3.136 | 0.448 | 0.219608 | 0 | 3 | 3 | 0 | 1 | 0 | Ephrin type-A receptor 2 | TRANSFERASE | EPHA2_HUMAN | Receptor for members of the ephrin-A family. Binds to ephrin-A1, -A3, -A4 and -A5. |
| 1r1j_v | 8 | 3.574 | 0.4468 | 1.52581 | 2 | 2 | 2 | 0 | 2 | 0 | Neprilysin | HYDROLASE | NEP_HUMAN | Thermolysin-like specificity, but is almost confined on acting on polypeptides of up to 30 amino acids. Biologically important in the destruction of opioid peptides such as Met- and Leu-enkephalins by cleavage of a Gly-Phe bond. Involved in the degradatio |
| 1cbs_v | 10 | 4.418 | 0.4418 | 0.436222 | 7 | 2 | 0 | 0 | 1 | 0 | Cellular retinoic acid-binding protein 2 | NONE | P29373 | Involved in retinoid binding |
| 1rd4_v | 6 | 2.639 | 0.4399 | -1.3028 | 5 | 0 | 0 | 1 | 0 | 0 | Integrin alpha-L | IMMUNE SYSTEM | ITAL_HUMAN | Integrin alpha-L/beta-2 is a receptor for ICAM1, ICAM2, ICAM3 and ICAM4. It is involved in a variety of immune phenomena including leukocyte-endothelial cell interaction, cytotoxic T-cell mediated killing, and antibody dependent killing by granulocytes an |
| 1u4l_v | 6 | 2.622 | 0.437 | -1.11238 | 0 | 5 | 1 | 0 | 0 | 0 | C-C motif chemokine 5 | ATTRACTANT | CCL5_HUMAN | Chemoattractant for blood monocytes, memory T-helper cells and eosinophils. Causes the release of histamine from basophils and activates eosinophils. Binds to CCR1, CCR3, CCR4 and CCR5. One of the major HIV-suppressive factors produced by CD8+ T- cells. R |
| 1uym_v | 8 | 3.48 | 0.435 | 1.2587 | 5 | 1 | 1 | 0 | 0 | 1 | Heat shock protein HSP 90-beta | NONE | P08238 | Posttranslational modification, protein turnover, chaperones |
| 2o9i_v | 7 | 3.034 | 0.4335 | -0.43839 | 5 | 2 | 0 | 0 | 0 | 0 | Nuclear receptor subfamily 1 group I member 2 | TRANSCRIPTION | NR1I2_HUMAN | Orphan receptor; its natural ligand is probably pregnane. Binds to a response element in the CYP3A4 and ABCB1/MDR1 genes promoter. Activates its expression in response to a wide variety of endobiotics and xenobiotics. |
| 3d2e_v | 6 | 2.592 | 0.4321 | -1.06564 | 0 | 4 | 2 | 0 | 0 | 0 | Heat shock protein homolog SSE1 | NONE | P32589 | Has a calcium-dependent calmodulin-binding activity. Required for normal growth at various temperatures. |
| 1x0n_v | 9 | 3.865 | 0.4295 | 1.61854 | 5 | 1 | 2 | 0 | 1 | 0 | Growth factor receptor-bound protein 2 | PEPTIDE BINDING PROTEIN | GRB2_HUMAN | Adapter protein that provides a critical link between cell surface growth factor receptors and the Ras signaling pathway.;Isoform GRB3-3 does not bind to phosphorylated epidermal growth factor receptor (EGFR) but inhibits EGF-induced transactivation of a RAS-responsive element. Isoform GRB3-3 acts as a dominant negative protein over GRB2 and by suppressing proliferative sign |
| 1kv3_v | 5 | 2.139 | 0.4278 | -1.32392 | 0 | 2 | 1 | 0 | 1 | 1 | Protein-glutamine gamma-glutamyltransferase 2 | TRANSFERASE | TGM2_HUMAN | Catalyzes the cross-linking of proteins and the conjugation of polyamines to proteins. |
| 1gkd_v | 7 | 2.989 | 0.4269 | -0.27543 | 1 | 4 | 2 | 0 | 0 | 0 | Matrix metalloproteinase-9 | HYDROLASE | MMP9_HUMAN | May play an essential role in local proteolysis of the extracellular matrix and in leukocyte migration. Could play a role in bone osteoclastic resorption. Cleaves KiSS1 at a Gly- |
| 1nav_v | 7 | 2.985 | 0.4265 | -0.63277 | 4 | 2 | 1 | 0 | 0 | 0 | Thyroid hormone receptor alpha | NONE | P10827 | Involved in transcription factor activity |
| 1gzu_v | 7 | 2.968 | 0.424 | -0.35438 | 1 | 4 | 1 | 0 | 1 | 0 | Nicotinamide mononucleotide adenylyltransferase 1 | NONE | Q9HAN9 | Coenzyme transport and metabolism |
| 1fcz_v | 7 | 2.966 | 0.4237 | -0.42898 | 6 | 0 | 0 | 0 | 1 | 0 | Retinoic acid receptor gamma | GENE REGULATION | RARG_HUMAN | This is a receptor for retinoic acid. This metabolite has profound effects on vertebrate development. Retinoic acid is a morphogen and is a powerful teratogen. This receptor controls cell function by directly regulating gene expression. |
| 1h0c_v | 7 | 2.959 | 0.4227 | -0.49162 | 2 | 2 | 1 | 0 | 1 | 1 | Serine--pyruvate aminotransferase | TRANSFERASE | SPYA_HUMAN | NONE |
| 1t32_v | 7 | 2.957 | 0.4224 | -0.50016 | 3 | 3 | 0 | 0 | 1 | 0 | Cathepsin G | HYDROLASE | CATG_HUMAN | Serine protease with trypsin- and chymotrypsin-like specificity. |
| 1s19_v | 8 | 3.378 | 0.4223 | 0.744716 | 5 | 2 | 1 | 0 | 0 | 0 | Vitamin D3 receptor | GENE REGULATION | VDR_HUMAN | Nuclear hormone receptor. Transcription factor that mediates the action of vitamin D3 by controlling the expression of hormone sensitive genes. Plays a central role in calcium homeostasis. |
| 1mlw_v | 7 | 2.956 | 0.4223 | -0.49298 | 1 | 4 | 2 | 0 | 0 | 0 | Tryptophan 5-hydroxylase 1 | OXIDOREDUCTASE | TPH1_HUMAN | NONE |
| 1mt6_v | 7 | 2.951 | 0.4215 | -0.14415 | 2 | 2 | 2 | 0 | 1 | 0 | Histone-lysine N-methyltransferase SETD7 | TRANSFERASE | SETD7_HUMAN | Histone methyltransferase that specifically monomethylates Lys-4 of histone H3. H3 Lys-4 methylation represents a specific tag for epigenetic transcriptional activation. Plays a central role in the transcriptional activation of genes such as collagena |
| 1m6d_v | 7 | 2.95 | 0.4215 | -0.0618 | 3 | 2 | 2 | 0 | 0 | 0 | Cathepsin F | NONE | Q9UBX1 | Involved in cysteine-type endopeptidase activity |
| 1mp8_v | 7 | 2.945 | 0.4207 | -0.53839 | 0 | 3 | 3 | 0 | 1 | 0 | Focal adhesion kinase 1 | TRANSFERASE | FAK1_HUMAN | Non-receptor protein-tyrosine kinase implicated in signaling pathways involved in cell motility, proliferation and apoptosis. Activated by tyrosine-phosphorylation in response to either integrin clustering induced by cell adhesion or antibody cross-linkin |
| 1lv2_v | 7 | 2.933 | 0.419 | -0.72126 | 5 | 1 | 0 | 0 | 1 | 0 | Hepatocyte nuclear factor 4-gamma | NONE | Q14541 | Involved in transcription factor activity |
| 1tfg_v | 9 | 3.759 | 0.4177 | 1.15695 | 4 | 2 | 2 | 1 | 0 | 0 | Transforming growth factor beta-2 | GROWTH FACTOR | TGFB2_HUMAN | TGF-beta 2 has suppressive effects on interleukin-2 dependent T-cell growth. |
| 1ne7_v | 7 | 2.92 | 0.4171 | -0.31508 | 0 | 4 | 3 | 0 | 0 | 0 | Glucosamine-6-phosphate isomerase | NONE | P46926 | Carbohydrate transport and metabolism |
| 1qzu_v | 7 | 2.91 | 0.4157 | -0.06655 | 1 | 3 | 2 | 0 | 1 | 0 | Phosphopantothenoylcysteine decarboxylase | NONE | Q96CD2 | Involved in catalytic activity |
| 1kjl_v | 7 | 2.908 | 0.4155 | -0.18999 | 0 | 3 | 4 | 0 | 0 | 0 | Galectin-3 | SUGAR BINDING PROTEIN | LEG3_HUMAN | Galactose-specific lectin which binds IgE. May mediate with the alpha-3, beta-1 integrin the stimulation by CSPG4 of endothelial cells migration. Together with DMBT1, required for terminal differentiation of columnar epithelial cells during early embryoge |
| 1gbn_v | 8 | 3.317 | 0.4146 | 0.393769 | 2 | 4 | 0 | 0 | 2 | 0 | Ornithine aminotransferase, mitochondrial | TRANSFERASE | OAT_HUMAN | NONE |
| 1qh5_v | 7 | 2.898 | 0.414 | -0.48542 | 0 | 4 | 1 | 0 | 2 | 0 | Hydroxyacylglutathione hydrolase, mitochondrial | NONE | Q16775 | Involved in hydrolase activity |
| 1nn0_v | 8 | 3.307 | 0.4134 | 0.207921 | 1 | 5 | 1 | 0 | 1 | 0 | Thymidylate kinase | TRANSFERASE | KTHY_HUMAN | Catalyzes the conversion of dTMP to dTDP. |
| 1yq7_v | 8 | 3.3 | 0.4125 | -0.14947 | 1 | 4 | 1 | 0 | 2 | 0 | Farnesyl pyrophosphate synthetase | TRANSFERASE | FPPS_HUMAN | Key enzyme in isoprenoid biosynthesis which catalyzes the formation of farnesyl diphosphate (FPP), a precursor for several classes of essential metabolites including sterols, dolichols, carotenoids, and ubiquinones. FPP also serves as substrate for protei |
| 1zdz_v | 7 | 2.871 | 0.4102 | -0.42112 | 0 | 3 | 2 | 2 | 0 | 0 | Spermidine synthase | NONE | P19623 | Amino acid transport and metabolism |
| 1z57_v | 7 | 2.86 | 0.4086 | -0.6702 | 0 | 5 | 1 | 1 | 0 | 0 | Dual specificity protein kinase CLK1 | TRANSFERASE | CLK1_HUMAN | Phosphorylates serine- and arginine-rich (SR) proteins of the spliceosomal complex may be a constituent of a network of regulatory mechanisms that enable SR proteins to control RNA splicing. Phosphorylates serines, threonines and tyrosines (By similarity) |
| 1jcn_v | 7 | 2.859 | 0.4085 | -0.72149 | 0 | 5 | 1 | 0 | 1 | 0 | Inosine-5-monophosphate dehydrogenase 1 | OXIDOREDUCTASE | IMDH1_HUMAN | Rate limiting enzyme in the de novo synthesis of guanine nucleotides and therefore is involved in the regulation of cell growth. It may also have a role in the development of malignancy and the growth progression of some tumors. |
